# *In vivo* delivery strategies for therapeutic CRISPR genome editing

**DOI:** 10.7150/ijbs.133162

**Published:** 2026-07-11

**Authors:** Leonardo Martin, Jure Bohinc, Alessandra Recchia, Stefano Gritti, Giorgia Santilli, Joanna Zeyland, Melita Vidaković, Nevena Grdović, Karim Benabdellah, María Ortiz-Bueno, Bilge Debelec Butuner, Luana Pisaniello, Roberta Stilhano, Daniela Benati, Fatma Zehra Hapil, Shariqa Khawaja, Rajeevkumar Raveendran Nair, Carla Giacomelli, Erden Atilla, Federica Zinghirino, Tommaso Ferrari, Federico Corradi, Tamas J. Laufer, Denis Khnykin, Gloria González Aseguinolaza, Mojca Skrbinek, Tjaša Mlakar, Tjaša Lapanja, Duško Lainšček

**Affiliations:** 1 Department of Pharmaceutical Sciences, Laboratory of Physiopharmacology, University of Antwerp, Belgium.; 2 Infla-Med Centre of Excellence, University of Antwerp, Belgium.; 3 Department of Synthetic Biology and Immunology, National Institute of Chemistry, Ljubljana, Slovenia.; 4 Graduate School of Biomedicine, University of Ljubljana, Ljubljana, Slovenia.; 5 Department of Life Sciences, University of Modena and Reggio Emilia, Modena and Reggio Emilia, Emilia-Romagna, Italy.; 6 Infection, Immunity and Inflammation Research and Teaching Department, University College London Great Ormond Street Institute of Child Health, London, United Kingdom.; 7 Department of Biochemistry and Biotechnology, University of Life Sciences in Poznań, Poland.; 8 Institute for Biological Research Sinisa Stankovic, National Institute of the Republic of Serbia, University of Belgrade, Belgrade, Serbia.; 9 Department of Genomic Medicine, Andalusian Regional Government Centre for Genomics and Oncological Research (GENYO), Pfizer-University of Granada, Granada, Spain.; 10 Dept. of Pharmaceutical Biotechnology, Faculty of Pharmacy, Ege University, Izmir, Turkey.; 11 Department of Physiological Sciences, Santa Casa de São Paulo School of Medical Sciences, São Paulo, Brazil.; 12 Department of Medical Biology and Genetics, Akdeniz University School of Medicine, Antalya, Turkey.; 13 Centre for Regenerative Medicine and Stem Cell Research, Aga Khan University, Karachi, Pakistan.; 14 Kavli Institute for Systems Neuroscience, Faculty of Medicine, NTNU, Trondheim, Norway.; 15 Universidad Nacional de Córdoba, Facultad de Ciencias Químicas, Consejo Nacional de Investigaciones Científicas y Técnicas, Instituto de Investigaciones en Fisicoquímica de Córdoba, Argentina.; 16 Division of Transplantation and Cellular Therapy, Sylvester Comprehensive Cancer Center, Department of Medicine, Miller School of Medicine, University of Miami, Miami, FL, USA.; 17 Department of Biochemical Engineering, University College London (UCL), London, United Kingdom.; 18 Department of Pathology, Oslo University Hospital, Oslo, Norway.; 19 DNA & RNA Medicine Division, CIMA, University of Navarra, IdisNA, Pamplona, Navarra, Spain.; 20 Centre for Technologies of Gene and Cell Therapy, National Institute of Chemistry, Ljubljana, Slovenia.; 21 EN-FIST Centre of Excellence, Ljubljana, Slovenia.; 22 Veterinary Faculty, University of Ljubljana, Ljubljana, Slovenia

**Keywords:** *in vivo* genome editing, CRISPR delivery systems, base editing, prime editing, organ-specific gene therapy, lipid nanoparticles, viral vectors

## Abstract

CRISPR-based genome and epigenome editing technologies have rapidly evolved from programmable nucleases into a diverse therapeutic toolbox encompassing conventional CRISPR systems, base editing, prime editing, RNA targeting, and epigenetic modulation. While early clinical successes relied on *ex vivo* manipulation of patient-derived cells, recent advances in delivery chemistry and vector engineering are enabling direct *in vivo* editing across multiple organs. Here, we provide a comprehensive review of delivery modalities of CRISPR systems solely *in vivo* that underpin their therapeutic translation. We examine how anatomical, cellular, and immunological constraints shape organ-specific editing strategies in different organ systems and we highlight key preclinical and clinical milestones that define the current translational landscape. Across indications, delivery remains a critical determinant of efficacy, safety, and scalability, governing editor exposure, tissue selectivity, and risk of unintended genomic or epigenomic perturbation. This review, authored by members of the COST Action Genome Editing to treat Human Diseases (GenE-HumDi) Network, delineates the principles guiding *in vivo* genome and epigenome editing and outlines the remaining barriers to durable, tissue-selective, and broadly deployable CRISPR therapeutics.

## Introduction

Genome editing enables the precise and efficient manipulation of DNA sequences within the genome and has demonstrated therapeutic potential across a broad spectrum of genetic diseases. Originally identified as part of the bacterial adaptive immune system, the CRISPR (Clustered Regularly Interspaced Short Palindromic Repeats) relies on RNA-guided endonucleases to recognize and cleave invading nucleic acids. In its engineered form, the system employs a single guide RNA (gRNA), formed by fusion of two native RNA components, to direct a CRISPR-associated (Cas) nuclease to a complementary genomic target adjacent to a protospacer-adjacent motif (PAM), where it introduces a site-specific double-strand break (DSB)^1^,^2^.

Since its adaptation as a genome engineering tool, multiple Cas nuclease families and orthologs have been identified and engineered, expanding the versatility of programmable genome editing. Among these, *Streptococcus pyogenes* Cas9 (SpCas9) remains the most widely used due to its robust nuclease activity and extensive biochemical characterization^3^,^4^. Additional Cas9 orthologs from *Staphylococcus aureus* (SaCas9), *Neisseria meningitidis* (NmeCas9), *Campylobacter jejuni* (CjCas9), and *Staphylococcus auricularis* (SauriCas9) differ in size, PAM recognition, gRNA scaffold requirements, optimal spacer length, and editing specificity. Smaller Cas9 variants offer advantages for *in vivo* applications, particularly when delivery is constrained by vector cargo limits, such as adeno-associated virus (AAV) systems, or when non-viral platforms favor compact payloads. However, compared to SpCas9, many smaller Cas9 orthologs generally exhibit reduced editing efficiency, necessitating careful trade-offs between size and activity^5^-^7^.

To expand the targetable genomic space while maintaining high nuclease activity, SpCas9 has been further engineered through rational design, directed evolution, and phage-assisted continuous evolution. These efforts have produced variants with relaxed or near-PAMless recognition, including SpCas9-NG, xCas9^8^, and SpRY^9^, substantially broadening the range of editable genomic loci (**Table 1**).

To diversify CRISPR applications beyond indel-based gene disruption and to enable precise correction of single-nucleotide variants, Cas9 has been engineered into a nickase by inactivating one of its nuclease domains. Furthermore, fusion of Cas9, containing the inactivated RuvC domain (D10A-nCas9) with cytidine or adenosine deaminases has enabled the development of base editors, which catalyze programmable base transitions within a defined editing window without inducing double-strand breaks^10^.

Cytosine base editors and adenine base editors have been widely applied to introduce point mutations, generate premature stop codons, disrupt start codons, and correct pathogenic single-nucleotide polymorphisms. To improve specificity and reduce unwanted DNA and RNA off-target activity, multiple engineered base editor variants have been developed. For cytosine base editors, these include SECURE-BE3, eA3ABE3, high-fidelity BE4max-derived editors, and more recently QBEmax, which demonstrates markedly improved product purity and reduced indel formation. For adenine base editors, optimized variants such as ABE7.10, ABEmax, ABE8e, ABE9, and SECURE-ABE have been developed to narrow editing windows, reduce RNA off-targeting, and broaden target scope^11^-^14^. Notably, many specificity-enhancing mutations can reduce deaminase activity, underscoring the need to balance efficiency and precision, particularly in therapeutic contexts.

Despite their precision, the relatively large size of base editors poses significant challenges for *in vivo* delivery. Conventional editors incorporating SpCas9 exceed the approximately 4.7 kilobase packaging limit of AAV vectors, often necessitating dual-vector delivery strategies such as fragmented, overlapping, or trans-splicing constructs. Among these approaches, split-intein-based systems have emerged as the most widely adopted solution for reconstituting full-length editors *in vivo*^15^.

In 2019, Anzalone and colleagues introduced prime editing, a versatile genome editing strategy capable of installing all twelve possible base substitutions as well as precise insertions and deletions without generating double-strand breaks or requiring an exogenous DNA donor. The prime editing (PE) system consists of a Cas9 nickase, in which the HNH domain is inactivated (H840A-nCas9), and fused to a reverse transcriptase derived from Moloney murine leukemia virus, together with a prime editing guide RNA that encodes both the targeting sequence and the desired edit^16^.

Upon binding to the target locus, the Cas9 nickase introduces a single-strand break in the non-target strand, exposing a 3′ hydroxyl group that anneals to the primer binding site within the prime editing guide RNA. The reverse transcriptase then synthesizes a new DNA strand encoding the intended edit, generating competing 3′ and 5′ DNA flaps. Although the unedited flap is thermodynamically favored, cellular nucleases preferentially degrade it, allowing incorporation of the edited strand. Subsequent DNA repair and mismatch resolution establish the modification on both strands^16^.

Successive generations of PE systems have been developed to enhance efficiency and fidelity. PE1 represents the original configuration, while PE2 incorporates an engineered reverse transcriptase with improved activity. PE3 further increases editing efficiency by introducing an additional nick on the non-edited strand, albeit at the cost of increased insertion and deletion formation. To mitigate this effect, PE3b employs an edit-dependent nicking strategy that preferentially targets the edited strand after flap resolution. Furthermore, the PE system was improved with generations PE4 and PE5 by transiently inhibiting mismatch repair through expression of a dominant-negative MLH1 variant, leading to substantial gains in editing efficiency^17^. The latest generation of prime editors, the PE6 family, introduces a suite of editors optimized for distinct parameters such as editor size, AAV compatibility and highly structured reverse transcription template (RTT)^18^. Additionally, PE7 emerged with the inclusion of the small RNA-binding exonuclease protection factor La^19^. Compared with base editing, prime editing offers broader sequence versatility but remains limited in the size of insertions or deletions that can be introduced.

Beyond permanent genome modification, CRISPR-based platforms have enabled precise and programmable modulation of gene expression without altering DNA sequence, establishing the field of epigenome editing. These systems typically consist of a catalytically inactive Cas9 fused to epigenetic effector domains, which are guided by a gRNA to specific genomic loci^20^.

A wide range of dCas9-effector fusions has been developed, including transcriptional activators such as VP64, histone acetyltransferases such as p300, DNA methyltransferases such as DNMT3A, demethylases such as TET1, and transcriptional repressors such as KRAB. Targeted recruitment of these effectors enables locus-specific modulation of chromatin state, resulting in gene activation or repression with high precision^21^,^22^. Signal amplification strategies, including engineered gRNA scaffolds containing RNA aptamers and multivalent effector recruitment systems, further enhance regulatory potency^23^.

Successive generations of epigenome editors have expanded functional versatility. These include first-generation direct fusion systems such as dCas9-VP64 and dCas9-p300, second-generation multimeric platforms such as SunTag and Synergistic Activation Mediator (SAM), and third-generation multiplexed editors enabling combinatorial regulation of multiple epigenetic marks. More recently, CRISPRoff and CRISPRon systems have enabled reversible and heritable gene silencing through programmable DNA methylation without double-strand break formation^24^.

Emerging *in vivo* applications of epigenome editing have demonstrated therapeutic potential in animal models, including targeted repression of the amyloid precursor protein gene in Alzheimer's disease models^25^, stable promoter hypermethylation in hematopoietic stem and progenitor cells, and tissue-specific activation or repression using conditional dCas9-based mouse lines^26^. Clinically oriented studies have also explored epigenetic correction of disease-associated regulatory states, such as reactivation of fetal hemoglobin in beta-thalassemia or repression of oncogenic transcriptional programs in cancer^27^. Despite these advances, off-target chromatin modulation and delivery efficiency remain major challenges. Ongoing efforts to improve specificity include engineering alternative dCas9 orthologs, introducing accuracy-enhancing mutations, and expanding the toolkit to include Cas12a- and Cas13-based systems for DNA- and RNA-level regulation^28^.

Collectively, the expanding repertoire of CRISPR-based genome and epigenome editing technologies provides unprecedented control over genetic and regulatory states. However, translating these tools into safe and effective *in vivo* therapies critically depends on the development of delivery strategies capable of achieving precise targeting, sufficient efficiency, and acceptable specificity at the organismal level.

A unifying principle emerging across *in vivo* genome editing studies is that delivery, rather than the editing modality itself, represents the primary determinant of therapeutic success. Delivery systems define the magnitude and duration of editor exposure, tissue specificity, safety profile, and feasibility of repeat dosing, thereby establishing the translational boundaries of CRISPR-based therapies.

## Overview of CRISPR Delivery Modalities and Cargo-Dependent Design Considerations

Efficient delivery of CRISPR components into target cells is a critical determinant of genome editing success. The CRISPR system can be delivered in multiple formats, including plasmid DNA, messenger RNA, or as a pre-assembled ribonucleoprotein (RNP) complex. Delivery strategies are broadly classified into viral and non-viral systems, each offering distinct advantages and limitations depending on the nature of the CRISPR cargo, the target tissue, and the desired duration of nuclease activity. Key considerations include editing efficiency, safety profile, immunogenicity, scalability, and regulatory compatibility^29^.

Viral vectors have historically been the most widely used delivery platforms due to their intrinsic ability to efficiently enter cells and mediate intracellular expression of genetic material. Commonly employed viral systems include lentiviruses, adenoviruses, and AAVs. Lentiviral vectors integrate into the host genome, enabling stable and long-term expression of Cas9 and sgRNA, which is advantageous for applications requiring sustained editing, such as in stem cells or proliferating cell populations^30^,^31^. In contrast, AAV vectors are largely non-integrating, exhibit relatively low immunogenicity, and are particularly suitable for *in vivo* applications. However, their limited packaging capacity constrains the size of Cas variants, regulatory elements, or multi-component editing systems that can be delivered^32^.

While viral delivery systems provide high transduction efficiency and robust expression, they are associated with important limitations, including immunogenicity, insertional mutagenesis in integrating vectors, and increased regulatory complexity for clinical translation^33^. These platforms are particularly well suited for DNA-based CRISPR constructs that rely on intracellular transcription and prolonged expression. However, they are less optimal for transient delivery strategies, such as those involving Cas9 RNPs, where short-lived nuclease activity is preferred to reduce off-target editing^34^.

Non-viral delivery approaches provide an alternative strategy that prioritizes safety, modularity, and flexibility^35^. These systems encompass physical methods, such as electroporation, microinjection, and hydrodynamic injection, as well as chemical carriers, including lipid nanoparticles (LNPs), polymeric nanoparticles, and cell-penetrating peptides^36^. Non-viral platforms are particularly attractive for delivering Cas9 mRNA or RNP complexes, enabling transient expression and rapid clearance of the editing machinery.

The primary advantages of non-viral delivery systems include the absence of insertional mutagenesis, reduced immunogenicity, and the capacity to transport a broad range of cargo types, including plasmid DNA, mRNA, small interfering RNA (siRNA), and CRISPR/Cas components^37^. These systems also offer precise control over cargo composition and surface functionalization for targeted delivery, and they are generally more compatible with large-scale manufacturing and regulatory approval processes compared with viral vectors^38^.

Despite these advantages, non-viral delivery approaches often suffer from lower transfection efficiency, shorter duration of gene expression, and challenges in achieving robust *in vivo* targeting, particularly in specific tissues or stem cell populations^39^. Additional barriers include endosomal entrapment, instability in circulation, and rapid clearance by the reticuloendothelial system, all of which can limit therapeutic efficacy^40^. Nevertheless, ongoing advances in nanocarrier engineering, biomimetic surface coatings, and ligand-based targeting strategies continue to improve the performance of non-viral vectors, enhancing their potential for clinical translation^41^,^42^.

Emerging hybrid delivery platforms, including virus-like particles, engineered extracellular vesicles (EVs), and functionalized nanoparticles, seek to combine the high efficiency of viral systems with the safety and adaptability of non-viral approaches. These systems are particularly promising for the delivery of CRISPR RNPs or mRNA, offering targeted delivery, improved biocompatibility, and reduced risk of long-term genomic integration, making them attractive candidates for *in vivo* genome editing applications.

A direct comparison of delivery platforms highlights fundamental translational trade-offs. Viral systems, particularly AAV, provide high delivery efficiency and well-defined tissue tropism, but are limited by restricted cargo capacity, immunogenicity, and the inability to support repeat dosing. In contrast, non-viral platforms such as LNPs enable transient editor expression, improved safety profiles, and scalable manufacturing, but generally exhibit lower delivery efficiency and less predictable tissue targeting beyond the liver. These complementary strengths and limitations emphasize that no single platform satisfies all therapeutic requirements. These platform-specific characteristics are summarized in **Table 2**, which provides a structured comparison of viral and non-viral delivery systems across key parameters relevant for clinical translation.

In summary, the choice of CRISPR delivery strategy must be guided by the specific editing modality and therapeutic objective. Viral vectors provide high efficiency and sustained expression, making them well suited for DNA-based CRISPR constructs, whereas non-viral platforms enable transient and potentially safer delivery of mRNA or RNP formats. Hybrid systems offer a promising intermediate solution. A clear understanding of the advantages and limitations of each delivery class is essential for the rational design and successful implementation of CRISPR-based genome editing in both research and therapeutic settings.

To facilitate cross-platform comparison and support rational selection of delivery strategies, the major CRISPR delivery modalities are summarized in a structured design matrix (**Table 3**), integrating key parameters including cargo format, expression duration, dosing considerations, mechanisms of tissue selectivity, safety profiles, and Chemistry, Manufacturing and Controls (CMC)-related challenges.

## Non-viral Delivery of CRISPR System

Non-viral delivery systems have gained increasing attention as they enable transient and potentially safer genome editing, but their performance remains strongly dependent on overcoming biological barriers to delivery, particularly efficient tissue targeting and endosomal escape.

**Figure 1:**
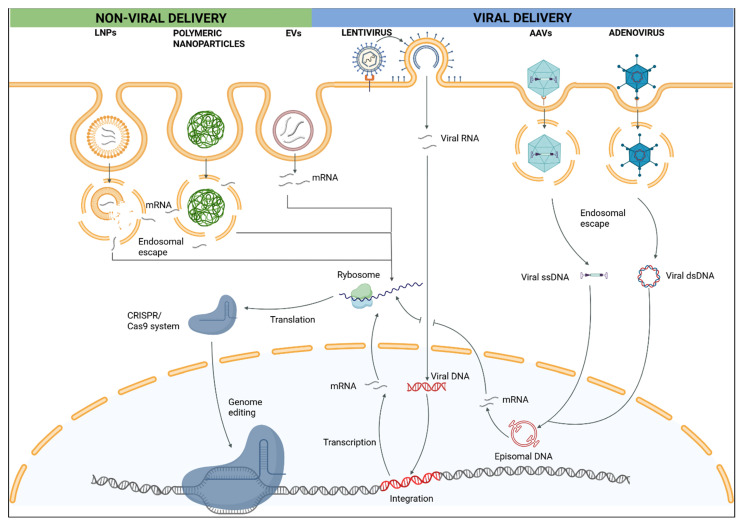
Comparison of non-viral and viral delivery platforms for CRISPR/Cas9 genome editing. Schematic overview of major non-viral and viral delivery strategies used to transport CRISPR/Cas components into target cells. Non-viral delivery systems include lipid nanoparticles (LNPs), polymeric nanoparticles, and extracellular vesicles (EVs), which primarily encapsulate CRISPR cargo in the form of mRNA or ribonucleoprotein (RNP) complexes. Following cellular uptake via endocytosis, these systems rely on endosomal escape to release mRNA into the cytosol, where it is translated into Cas9 protein, enabling subsequent genome editing upon nuclear entry. Viral delivery platforms, including lentivirus, adeno-associated virus (AAV), and adenovirus, enter cells through receptor-mediated endocytosis and deliver viral RNA or DNA genomes. Lentiviral vectors undergo reverse transcription and integration into the host genome, AAV vectors typically persist as episomal DNA and adenoviral vectors deliver double-stranded DNA without genomic integration. Transcription of viral genomes leads to sustained Cas9 expression, which can enhance editing efficiency but also prolong nuclease activity. The figure highlights key mechanistic differences between transient, non-integrating non-viral approaches and long-lasting viral systems, illustrating trade-offs in delivery efficiency, durability of expression, and safety considerations. Created in BioRender. Lainscek, D. (2026) https://BioRender.com/jbwftjc.

The advancement of non-viral delivery strategies (**Figure 1**) is largely driven by their ability to overcome key limitations of viral vectors, including immunogenicity, off-target effects, and restricted cargo capacity. Compared with viral approaches, non-viral platforms offer improved safety through reduced immune activation and greater flexibility due to tunable physicochemical properties and the ability to accommodate larger and more diverse payloads. However, non-viral delivery of gene editors still faces significant challenges, most notably lower delivery efficiency relative to viral systems. This reduced efficiency arises from multiple biological barriers, including biocompatibility issues, toxicity, immunogenicity, and intracellular cargo release. Importantly, these limitations can be addressed through rational design and fine-tuning of non-viral delivery systems, ultimately improving the safety and efficacy of gene-editing therapies^43^.

Non-viral delivery systems are roughly divided into two categories: physical delivery (electroporation, microinjection and hydrodynamic injections) and carrier-mediated delivery (LNPs, polymer-based nanoparticles, peptide-based particles, inorganic nanocarriers and extracellular vesicles). Physical delivery methods use different modes of action to facilitate penetration of cargo through the cell membrane. For example, microinjection, allows delivery of cargo directly into a cell using micromanipulator with a microneedle and a high-resolution inverted microscope. During hydrodynamic injection a large volume of solution containing the gene-editing cargo is rapidly pushed into the bloodstream, usually through a tail vein in mice. Finally, electroporation is a well-established technique for introducing gene editing tools into different cells *in vitro* and *in vivo*. This technique employs pulses of high voltage electrical currents to disrupt the lipid bilayer, creating pores through which components can enter the cells. It is one of the most popular physical delivery methods due to its ease of use and versatility across different cargo and cell types^44^,^45^.

Carrier-mediated delivery, on the other hand, enables simultaneous protection and delivery of the cargo. LNPs are probably one of the most popular non-viral delivery vehicles for introducing gene editing tools *in vivo*. They are produced through rapid mixing of lipid components dissolved in ethanol and nucleic acids dissolved in acidic solution using a microfluidic device. The positive charge of lipids naturally interacts with the negatively charged nucleic acids thus forming a stable structure that protects the cargo inside. Following *in vivo* administration via intravenous injection, LNPs are mostly taken up by the liver. Therefore, LNPs have mostly been used for liver delivery of gene editing tools, while non-hepatic and tissue-specific delivery remain a significant challenge^46^. Other forms of particles that can be used as gene editing delivery tools include polymer particles and inorganic nanoparticles, for example polyethyleneimine (PEI) and polyethylene glycol (PEG). Polymers are able to form highly versatile molecular complexes with gene editing tools that can be further engineered to enhance cell or tissue specific targeting. Moreover, inorganic nanoparticles, including metal nanoparticles, metal-organic frameworks, carbon nanotubes and mesoporous silica nanoparticles, have also been investigated for delivery of gene editing tools. Inorganic nanoparticles are attractive delivery vehicles because their size and structure can be precisely tuned for specific applications. Moreover, their biological applications are well established, making them a versatile platform for gene-editing delivery. However, their cellular biocompatibility must be experimentally validated before use. Their tunable physicochemical properties allow control over size, surface chemistry, and targeting, positioning nanoparticle-based delivery, and LNPs in particular, as leading non-viral platforms for *in vivo* CRISPR genome editing^47^.

Finally, extracellular vesicles, which are naturally secreted by different cell types into the extracellular space, are also promising vehicles for delivering CRISPR/Cas9 components into cells. Their natural role is intracellular communication through the transfer of different cargo (e.g. proteins) between cells. Due to their inherent properties, including evasion of phagocytosis, efficient crossing of biological barriers, biocompatibility, and strong *in vivo* stability, EVs have the potential to serve as highly efficient delivery vehicles. However, further optimization is required to enhance their efficacy, for example by improving encapsulation and promoting efficient endosomal escape of the cargo, in order to expand their practical application^48^.

### Physical Non-viral Delivery Methods for CRISPR-based Genome Editing

**Figure 2:**
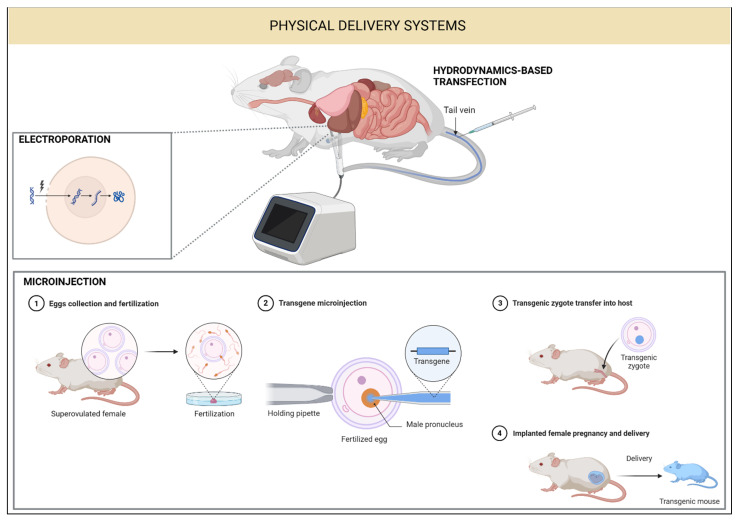
Physical delivery systems for genome editing *in vivo*. Schematic overview of physical approaches used to deliver genome-editing payloads into cells and tissues. Electroporation employs brief electrical pulses to transiently permeabilize cell membranes, enabling entry of plasmid DNA, mRNA, or ribonucleoprotein (RNP) complexes, and is primarily used in localized tissues or experimental models. Hydrodynamics-based transfection involves rapid, high-volume tail vein injection, generating transient intravascular pressure that promotes nucleic acid uptake, most commonly in hepatocytes. Microinjection consists of direct delivery of transgenes or genome-editing reagents into fertilized oocytes, followed by embryo transfer to generate transgenic animals. Although highly effective, these methods are invasive and largely restricted to preclinical applications. Created in BioRender. Lainscek, D. (2026) https://BioRender.com/h9ibvqp.

Physical non-viral delivery methods (**Figure 2**) represent direct and experimentally robust approaches for introducing CRISPR components into cells and tissues. Hydrodynamic injection, microinjection, and electroporation have been instrumental in establishing CRISPR-based genome editing, particularly in animal model generation, functional genomics, and *ex vivo* applications.

Hydrodynamic injection involves rapid intravenous administration of a nucleic acid solution into rodents at volumes corresponding to approximately 8-10% of body weight, most commonly via the tail vein. The resulting transient hydrodynamic pressure induces temporary membrane permeabilization in endothelial and parenchymal cells, enabling efficient uptake of nucleic acids, particularly by hepatocytes^48^. This method has been widely used to deliver plasmid-based CRISPR-Cas9 systems to the liver, enabling efficient gene disruption and correction *in vivo*^49^. Representative studies demonstrated correction of the Fah mutation in a mouse model of hereditary tyrosinemia and efficient somatic mutagenesis of *Pten*^50^,^51^ in hepatocytes following hydrodynamic delivery of pX330-based CRISPR constructs. Despite its effectiveness in small animals, hydrodynamic injection induces substantial hemodynamic stress and is not readily applicable to large animals or clinical settings, with hepatotoxicity observed in early clinical attempts^52^.

Microinjection is a precise mechanical technique involving direct delivery of CRISPR components into individual cells using fine glass micropipettes. In the context of genome editing, microinjection has been widely used to introduce plasmid-based CRISPR-Cas9 systems or Cas9 mRNA and sgRNA into fertilized zygotes, enabling rapid generation of genetically modified animal models^53^-^55^. This approach has been successfully applied across multiple species, including monkeys, zebrafish, invertebrates, and insects. To reduce risks associated with plasmid integration and prolonged Cas9 expression, microinjection of Cas9 mRNA and sgRNA has been shown to improve editing efficiency and safety compared with plasmid delivery. While highly accurate, microinjection is technically demanding, low throughput, and limited to applications involving small numbers of cells^56^.

Over the past decade, electroporation has become widely recognized as the gold standard for *ex vivo* delivery of CRISPR-based genome editors and remains central to clinical genome editing of hematopoietic stem cells and T cells, owing to its high efficiency and reproducibility across diverse cell types^57^. *In vivo*, the technique has been applied most extensively to tissues amenable to direct electrode placement, including embryonic and postnatal brain, skin, skeletal muscle, and liver following the intraparenchymal or intramuscular injection of CRISPR cargo. For example, local injection of CRISPR cargo, coupled with precisely positioned plate or needle electrodes, generates robust on-target mutagenesis and somatic mosaic models that faithfully recapitulate human pathology^58^. Subretinal electroporation exemplifies this approach by delivering CRISPR/Cas9 plasmids to correct mutant rhodopsin genes in the mouse retina, thereby modeling retinitis pigmentosa^59^. Transdermal methods using microneedle arrays enable deep plasmid penetration into dermal cells, allowing for the establishment of skin disease models. Intracranial and *in utero* electroporation extend these capabilities to deeper structures, providing precise spatial and temporal control over Cas9-mediated knockouts for studies of neurodevelopmental disorders and neural circuit dynamics^60^. In parallel, skeletal muscle electroporation has achieved therapeutic editing of the DMD gene, restoring partial dystrophin expression and yielding measurable functional improvements^61^. Despite these preclinical advances, *in vivo* electroporation remains restricted to small animal models and requires direct surgical or superficial electrode access, with no clinical applications in humans beyond *ex vivo* hematopoietic stem cell and T cell editing. Translational challenges arise primarily from the need for invasive electrode delivery to deep human organs such as the liver, heart, or brain. While this is feasible in rodents through externalization or surgery, it is impractical in patients without percutaneous arrays. These challenges are further compounded by local tissue trauma, inflammatory responses to electrode insertion, and the risk of current- or heat-induced damage at therapeutic scales in human tissues.

### Cell-penetrating Peptides

Cell-penetrating peptides (CPPs) are short peptides, typically 5-30 amino acids in length, enriched in cationic and/or amphipathic residues, that facilitate intracellular delivery of macromolecules via direct membrane translocation or endocytic uptake. CPPs can be classified according to origin, physicochemical properties, charge distribution, structure, and cellular entry mechanism, with extensive diversity and limited sequence homology across families^62^. Uptake efficiency and intracellular fate are strongly influenced by peptide sequence, cargo type, linkage strategy, cell lineage, and extracellular conditions^63^. In the context of CRISPR/Cas genome editing, CPPs have emerged as non-viral carriers for Cas9 RNP complexes and, in some cases, Cas9 mRNA, enabling transient genome modification without permanent vector integration. CPPs employ two principal cellular entry pathways: energy-independent direct translocation and energy-dependent endocytosis. Widely used CPPs such as TAT predominantly rely on endocytic uptake and therefore require efficient endosomal escape to release cargo into the cytosol. In contrast, endosomolytic peptides, such as HA2 derived from influenza virus, undergo pH-dependent conformational changes in acidic endosomes, promoting membrane destabilization and cargo release, albeit often at the cost of reduced solubility or increased hydrophobicity^64^. To overcome these limitations, chimeric CPPs combining cell-penetrating and endosomal escape domains (e.g., TAT-HA2 hybrids) have been developed, enabling efficient Cas9 RNP delivery through simple co-incubation without specialized equipment^65^. Zuris *et al*. demonstrated that self-delivering Cas9 constructs incorporating CPP-like elements enabled efficient genome editing in primary human cells with lower toxicity than electroporation-based delivery, the action of them was also determined *in vivo*^66^. CPP-mediated RNP delivery has also demonstrated robustness and practical advantages *in vitro*. Shalay *et al*. combined PepFect14 (PF14) with a rabies virus glycoprotein fragment to form neuron-targeting CPP nanocomplexes, enabling selective genome editing in neuronal cells with potential relevance for neurodegenerative diseases^62^.CPPs, by contrast, can be formulated as injectable nanocomplexes or conjugates and have demonstrated *in vivo* delivery capabilities, either alone or in combination with nanoparticle platforms. For example, CPP-decorated lipid nanoparticles have been used to enhance blood-brain barrier (BBB) penetration via transferrin receptor targeting, achieving measurable delivery without detectable tissue damage^67^. Editing efficiencies using CPP remain more variable and strongly dependent on peptide sequence, cargo stoichiometry, and endosomal escape efficiency. CPPs also lack intrinsic tissue- or cell-type specificity unless combined with targeting ligands, and systemic biodistribution remains difficult to control. Consequently, CPPs are increasingly explored within hybrid strategies, including CPP-lipid or CPP-polymer assemblies, designed to combine the high cell viability of peptide-mediated transport with the stability and targeting capabilities of nanoparticle platforms^68^.

### Lipid Nanoparticles

LNPs have emerged as one of the most effective non-viral platforms for the *in vivo* delivery of CRISPR-based genome editing systems, offering a favorable balance between efficiency, safety, and clinical translatability, with the majority of *in vivo* genome editing clinical trials relying on LNP^46^. These are chemically complex assemblies typically composed of ionizable lipids, phospholipids, cholesterol, and PEG lipids. LNPs function primarily by encapsulating and protecting CRISPR cargo through electrostatic interactions while facilitating their efficient cellular delivery. Negatively charged nucleic acids, including DNA, mRNA, or guide RNAs, are packaged via positively charged ionizable lipids, which shield the cargo from nuclease degradation and limit immune recognition during systemic circulation. Upon cellular uptake, ionizable lipids (e.g. ALC-0315 or SM-102) remain largely neutral at physiological pH, reducing systemic toxicity, but become protonated in the acidic endosomal environment, promoting membrane destabilization and endosomal escape, thereby releasing the cargo into the cytosol before lysosomal degradation. In addition to their favorable delivery properties, LNPs exhibit relatively high biocompatibility, low immunogenicity compared with viral vectors, and compatibility with scalable manufacturing processes. Importantly, LNP-mediated delivery of mRNA or ribonucleoprotein complexes enables transient genome editor expression (“hit-and-away” delivery), a feature that is particularly advantageous for genome editing applications as it minimizes prolonged nuclease activity and reduces the risk of off-target effects^69^-^71^.

LNPs have demonstrated effectiveness for *in vivo* editing in several tissues, but predominantly the liver, which is highly susceptible to LNP-mediated CRISPR delivery following intravenous administration^46^,^72^. Hepatic tropism is largely driven by adsorption of apolipoprotein E onto the LNP surface and subsequent uptake via low-density lipoprotein receptors. This property has enabled efficient disruption of genes such as *PCSK9* and *ANGPTL3*, leading to marked reductions in circulating lipid levels in animal models^73^,^74^.

To extend CRISPR delivery beyond the liver, significant effort has focused on engineering LNPs with altered biodistribution. Selective organ targeting (SORT) LNPs incorporate an additional lipid component to modulate surface charge and protein corona composition, thereby redirecting nanoparticles to extrahepatic tissues^38^,^72^. Cationic lipid-enriched SORT formulations preferentially target the lung, whereas anionic lipid-enriched formulations favor splenic delivery. Screening studies have identified lung-tropic LNP formulations capable of efficient endothelial and epithelial gene editing without detectable hepatic activity^75^. Conversely, transient macrophage depletion enhances liver-directed delivery while diminishing extrahepatic targeting, underscoring the role of immune clearance in shaping biodistribution^76^. Local administration strategies further expand the utility of LNPs by bypassing systemic barriers. Intramuscular and intratumoral injections have enabled efficient CRISPR-mediated editing in skeletal muscle (dystrophin restoration) and glioblastoma models, respectively^75^. Inhalation-based delivery through nebulization has shown promise for pulmonary genome editing^77^, while intracameral injection of LNPs enabled efficient and well-tolerated gene editing in ocular tissues^78^. Physicochemical tuning of LNPs, including particle size, lipid ratios, and apparent pKa, further influences organ specificity, with high pKa formulations favoring lung targeting and low pKa formulations accumulating in the spleen^46^.

Additional targeting specificity can be achieved through chemical modification of LNP surfaces, most commonly via functionalization of PEG lipids with antibodies, aptamers, or peptides^44^,^79^. Aptamer-conjugated LNPs have enabled tumor-selective CRISPR delivery^80^, while specialized ionizable lipids have facilitated targeting of immune cells and bone tissue^81^,^82^. Recent studies demonstrated improvements in selective organ targeting and efficient treatment results for various diseases. Tissue-selective LNP formulations encapsulating a thermostable Cas9 achieved genome-editing levels of 16‒37% in the liver and lungs in mouse model and revealed the therapeutic potential of thermostable Cas9 RNP-LNP complexes for genome editing^83^. LNP-mediated targeting of the *HAO1* gene, which encodes the liver-specific glycolate oxidase, has also been investigated in primary hyperoxaluria type 1, demonstrating successful delivery^84^. A personalized base-editing therapy has been shown to successfully treat severe carbamoyl-phosphate synthetase-1 deficiency by utilizing LNPs for safe delivery and enabling re-administration when necessary^85^. Similarly, a base editing (BE) strategy involving delivery of RNPs via LNPs formulated with the ionizable lipid SM102 has been shown to restore Rpe65 expression in hereditary retinal dystrophy^86^. Lu *et al*. developed a spleen-tropic LNP formulation engineered for preferential targeting of T cells in a mouse model, enabling efficient gene editing in mouse model supporting potential applications in HIV resistance and cancer immunotherapy^87^.

### Synthetic Nanomaterial-based Carriers

The use of synthetic nanomaterial-based delivery vehicles, including polymeric and inorganic nanoparticles, offers several advantages over viral vectors for gene editing, but also faces substantial challenges that limit clinical translation^88^. A primary advantage of these systems is their improved safety profile. In contrast to viral vectors, which frequently induce strong immune responses and neutralizing antibodies, non-viral and non-lipidic carriers generally exhibit lower immunogenicity and reduced toxicity. Many nanomaterials, such as poly(D,L-lactide-co-glycolide) (PLGA) and layered double hydroxides (LDHs), demonstrate favorable biocompatibility and biodegradability^89^. These carriers do not integrate into the host genome, they also avoid the risk of insertional mutagenesis associated with lentiviral vectors.

Synthetic nanomaterials provide considerable design flexibility, as their physicochemical properties, including size, shape, surface charge, and composition, can be precisely tuned to overcome biological barriers and enhance tissue targeting. These platforms can accommodate large CRISPR cargos in plasmid DNA, mRNA, or RNP formats, and enable co-delivery of multiple therapeutic agents. Their chemical synthesis supports scalable and cost-effective production, with some materials, such as LDHs, offering particular economic advantages. Importantly, delivery of mRNA or RNP allows transient genome-editing activity, reducing prolonged nuclease exposure, off-target effects, and genomic instability, while RNP delivery bypasses transcriptional and translational steps, accelerating the onset of editing^90^.

Despite these benefits, non-viral systems generally exhibit lower delivery efficiency than viral vectors and require extensive optimization. Dose-dependent cytotoxicity, long-term accumulation of inorganic nanoparticles, instability of CRISPR components in circulation, and limited reproducibility of complex formulations remain key challenges. Moreover, the lack of long-term biosafety studies in large-animal models underscores the need for comprehensive safety evaluations prior to clinical translation^91^,^92^.

Cationic polymers have been extensively employed as alternatives to lipids for *in vivo* transfection, forming polyplexes with nucleic acids via electrostatic interactions. Polymeric nanocarriers have been engineered to deliver genome editing components, showing efficacy in animal models^93^. PEI is among the most widely studied cationic polymers and is frequently regarded as the “gold standard” for small nucleic acid transfection owing to its high efficiency. It condenses large nucleic acid molecules into homogeneous spherical particles, facilitating cellular uptake. PEI-based nanoparticles modified with cholesterol and PEG were used to deliver a plasmid targeting vascular endothelial growth factor A (VEGFA) via intravenous injection in BALB/c nude mouse model of osteosarcoma. This treatment resulted in significant indel formation in osteosarcoma tissues and lung metastases, along with reduced VEGFA expression, leading to a significant reduction in primary tumor size and a decrease in the number of lung metastases^94^. Hyperbranched poly (β-amino esters) (HPAEs) and related poly(β-amino esters) (PBAEs) have been effectively utilized as polymeric non-viral vehicles in several *in vivo* genome editing procedures. A notable success was achieved with a novel HPAE-derived formulation for respiratory delivery. This system was engineered to deliver Cas13a mRNA and crRNA via nasal nebulization to treat SARS-CoV-2 infection in a hamster model^95^. This formulation was further highlighted as a species-agnostic delivery platform, with activity reported following nebulization in mice, hamsters, ferrets, cows, and non-human primates. Furthermore, PBAE nanoparticles were used to encapsulate plasmid Cas9 (pCas9) targeting the HPV16-E7 oncogene. Upon intratumoral injection, the PBAE nanoparticles efficiently delivered pCas9, resulting in the inhibition of tumor growth in models of HPV16-related cervical malignancy^96^. Furthermore, cholesterol (CHO)-terminated ethanolamine-aminated poly(glycidyl methacrylate) (CHO-PGEA) polymeric nanoparticle were designed to deliver *in vivo* a plasmid encoding Cas9 and gRNAs targeting the *Fbn1* gene, which is implicated in Marfan syndrome. To improve nanoparticle accumulation in the aorta, mice were pre-treated with Angiotensin II to increase vascular permeability. Subsequent *ex vivo* imaging confirmed enhanced nanoparticle delivery and efficient *Fbn1* knockout, with no observed toxicity^97^. Dendrimers are perfectly symmetric, spherical polymers possessing abundant secondary and tertiary amines that confer strong proton-buffering and nucleic acid-condensing abilities, leading to superior transfection efficiency. Boronic acid-rich dendrimers have been engineered for efficient cytosolic delivery of Cas9 RNPs. In addition, supramolecular nanoparticles self-assembled from β-cyclodextrin-grafted PEI, adamantane-grafted PAMAM, and adamantane-poly(ethylene glycol) have been employed for *in vivo* knock-in of the *Retinoschisis 1* gene^98^.

Inorganic nanomaterials provide multifunctional delivery platforms for genome editing, offering high stability, tunable surface chemistry, and versatile cargo loading. These systems, including two-dimensional materials and metal-based nanoparticles, have demonstrated therapeutic efficacy in multiple animal models. Black phosphorus nanosheets (BPs) are biodegradable inorganic carriers that enable enhanced cytosolic delivery of CRISPR/Cas9 payloads. In one approach, Cas9 RNP was engineered with three repeating nuclear localization signals (Cas9N3) to confer a positive charge, allowing efficient electrostatic loading onto negatively charged BPs. Following intratumoral injection into mice bearing A549/EGFP xenograft tumors, this formulation produced significant tumor regression over 20 days with no detectable toxicity, as confirmed by histological analysis. Efficient endosomal escape and nuclear delivery were observed *in vitro*, with a nuclear transport rate of 78% after 24 hours^99^.

Gold nanoparticles (AuNPs) are widely explored as inorganic carriers due to their tunable size and surface chemistry, enabling attachment of CRISPR components via ionic, covalent, or physical interactions. AuNPs are generally considered biocompatible and exhibit low cytotoxicity. However, their long-term fate remains a concern, as AuNPs accumulate primarily in clearance organs such as the liver and spleen following systemic administration. One prominent AuNP-based platform is CRISPR-Gold, which consists of a gold core functionalized with thiolated single-stranded DNA, followed by adsorption of Cas9 RNP and encapsulation within a cationic endosomal-disruptive polymer shell. This system has demonstrated therapeutic efficacy in mouse models of Duchenne muscular dystrophy and Fragile X syndrome^100^,^101^. In dystrophic mice, intramuscular injection achieved 5.4% homology-directed repair (HDR) of the dystrophin gene. In Fragile X models, intracranial injection reduced mGluR5 expression by 40-50% and rescued repetitive behaviors. Nevertheless, repeated dosing raises concerns about gold accumulation in neural tissue. AuNPs have also been applied in oncology. Hybrid constructs combining AuNPs with cationic peptides or lipid shells have delivered Cas9 protein and gRNA plasmids targeting polo-like kinase 1, leading to significant tumor growth suppression in melanoma models without overt toxicity. Arginine-functionalized AuNPs (ArgNPs) have further enabled systemic delivery of Cas9 RNP targeting *PTEN* by enhancing membrane fusion and bypassing endocytosis, although preferential accumulation in hepatic and splenic macrophages was observed^102^.

Silica nanoparticles, particularly mesoporous silica nanoparticles (MSNs), represent another major class of inorganic gene delivery vehicles due to their chemical versatility, tunable porosity, and general biocompatibility^103^. Large-pore MSNs can accommodate plasmid DNA, while lipid-coated MSNs (LC-MSNs) combine silica stability with membrane-mimetic properties for efficient CRISPR delivery^104^. Redox-sensitive silica nanocapsules incorporating disulfide linkages enable glutathione-triggered release of Cas9 RNPs and have achieved up to 38% gene editing in glioblastoma mouse models^105^. Despite their advantages, however, silica-based systems raise safety concerns due to limited biodegradability and tissue accumulation. High-dose exposure studies demonstrate dose- and organ-dependent toxicity, underscoring the need for careful design and long-term evaluation^106^. Iron oxide nanoparticles (IONs), including magnetite nanoparticles (MNPs), offer unique advantages due to their magnetic responsiveness, enabling spatial and temporal control of genome editing under external magnetic fields. Magnetofection enhances cellular uptake and localization, while magnetoelectric nanoparticles have been explored for non-invasive delivery across the BBB. However, as with other inorganic systems, MNPs are subject to sequestration by the reticuloendothelial system, resulting in accumulation in the liver and spleen^107^. Layered double hydroxides (LDHs) are emerging inorganic carriers characterized by low cost, good biocompatibility, and ease of functionalization^108^. Although primarily validated *in vitro*, limited *in vivo* studies demonstrate promising applications in cancer and neurological disease models. LDHs can facilitate endosomal escape through pH-buffering dissolution and can be engineered for redox-responsive or ligand-mediated targeting^109^.

Overall, inorganic nanomaterials offer powerful and tunable platforms for CRISPR delivery, particularly for enhancing endosomal escape and controlled cargo release. However, challenges related to biodegradability, long-term accumulation, and systemic safety remain key barriers to clinical translation, underscoring the importance of rational design and comprehensive *in vivo* evaluation.

### Extracellular vesicles

Extracellular vesicles (EVs) are nanosized, membrane-bound vesicles secreted by virtually all cell types across biological kingdoms and have emerged as promising non-viral carriers for therapeutic delivery, including CRISPR-based genome editing. They have emerged as promising non-viral carriers for therapeutic delivery, including CRISPR-based genome editing. EVs are commonly grouped into exosomes (approximately 30-150 nm), microvesicles or ectosomes (approximately 100-1,000 nm), and apoptotic bodies (approximately 1,000-5,000 nm). Exosomes are generated through the endosomal pathway via multivesicular bodies, microvesicles are produced by outward budding of the plasma membrane, and apoptotic bodies are released during programmed cell death. Beyond their structural diversity, EVs serve as biological mediators of intercellular communication by transporting proteins, nucleic acids, lipids, and metabolites that influence proliferation, apoptosis, immune signaling, and tissue homeostasis^110^,^111^.

Their intrinsic cargo capacity and membrane encapsulation make EVs attractive delivery vehicles for therapeutic macromolecules. EVs can package CRISPR cargos in multiple formats, including Cas9 RNPs, Cas9 mRNA with sgRNA, or plasmid DNA, while protecting these payloads from enzymatic degradation and improving systemic stability^112^. Cargo loading can be achieved through endogenous strategies, in which donor cells are engineered to express or recruit CRISPR components into EVs during vesicle biogenesis, or through exogenous strategies applied after vesicle isolation using membrane-permeabilization approaches such as electroporation, surfactants, or microfluidic loading. Endogenous approaches are generally more consistent and reproducible, and the EV lipid bilayer can extend functional half-life and bioavailability of CRISPR cargos *in vivo*^113^. Consistent with these mechanistic advantages, engineered EVs have increasingly been used as CRISPR delivery vehicles *in vivo*. RNP-loaded EVs have shown functional benefit in animal models, including restoration of hearing in a Shaker-1 model of progressive hearing loss and disruption of *PARP*-1 in ovarian tumor models, leading to apoptosis and enhanced chemosensitivity^114^.

Several modular EV systems illustrate the accelerating maturity of this delivery space. The safeEXO platform has been developed to deliver RNPs, mRNA, and other nucleic acids with dose-dependent editing, minimal immunogenicity, and no overt toxicity *in vivo*; in one study, safeEXO-CAS-ITGA6 vesicles increased uptake by lung epithelial cells and enabled lung-directed editing of *EMX1* in mice^115^. Other approaches have used chemically inducible heterodimerization combined with fusogenic components to package and deliver editing machinery, including the *ex vivo* disruption of *Pcsk9* in primary hepatocytes with downstream functional effects on LDL receptor activity^116^.

NanoMEDIC represents a distinct engineered EV-like strategy that integrates chemical dimerization and RNA packaging to deliver Cas9 and sgRNAs, supporting editing across diverse cell types including induced pluripotent stem cells, neurons, and myoblasts, and achieving durable exon skipping in Duchenne muscular dystrophy models following intramuscular administration^117^.

Beyond delivery efficiency, EVs offer favorable biocompatibility and opportunities for tissue targeting. EVs can be internalized via multiple endocytic routes and may traverse selective biological barriers, including passage across the BBB through transcytosis. Their targeting specificity can be further enhanced through surface engineering with ligands, antibodies, or peptides to promote cell-selective uptake. For example, engineered EVs incorporating active cargo-loading modules, T-cell-targeting domains, and fusogenic glycoproteins have enabled delivery of Cas9-sgRNA complexes to primary human CD4+ T cells for genome editing^118^.

Despite rapid progress, clinical translation remains constrained by variability in EV yield, heterogeneity in vesicle populations, and dependence on donor cell type and culture conditions. Standardized manufacturing workflows, improved control of cargo loading and release kinetics, and robust characterization of biodistribution and persistence will be essential to achieve reproducible, scalable, and safe EV-mediated genome editing for therapeutic use.

## Viral Delivery Systems for *In Vivo* Crispr Genome Editing

Viral vectors remain the most efficient delivery platforms for *in vivo* genome editing due to their evolved mechanisms of cellular entry and gene expression, but their clinical use is fundamentally constrained by payload size, immunogenicity, and limited redosing potential.

Viral delivery systems (**Figure 1**) exploit evolutionarily optimized mechanisms for cellular entry, intracellular trafficking, immune modulation, and gene expression, resulting in high transduction efficiency and robust *in vivo* gene delivery. Their capacity to be engineered for defined tissue tropism and sustained transgene expression has enabled widespread clinical adoption, supported by decades of preclinical development and regulatory experience in gene therapy^119^,^120^. In comparison with non-viral delivery approaches, viral vectors generally achieve superior delivery efficiency, more predictable biodistribution, and longer-lasting gene expression, albeit at the expense of increased immunogenicity, restricted payload capacity, and greater manufacturing complexity.

Different viral platforms provide complementary strengths that can be matched to specific genome-editing objectives. Lentiviral vectors enable stable genomic integration and long-term expression, making them particularly effective for stem cell-based and *ex vivo* genome-editing applications. AAVs support efficient *in vivo* transduction with minimal host genome disruption and have emerged as leading platforms for precision gene therapy. Adenoviral vectors offer high transduction efficiency and large payload capacity while remaining episomal, thereby reducing the risk of insertional mutagenesis. Substantial advances in vector engineering have improved safety profiles across these systems. Self-inactivating lentiviral vectors incorporate mutations in long terminal repeats that eliminate promoter activity and reduce genotoxic risk^121^, while non-integrating lentiviral vectors are generated through integrase inactivation or modification of integrase recognition sequences, preventing chromosomal insertion while preserving efficient delivery^122^.

Despite these refinements, viral delivery systems retain important limitations that are particularly relevant for CRISPR-based genome editing. Host immune responses can restrict redosing and reduce efficacy, especially following systemic administration. Integrating vectors carry a residual risk of insertional mutagenesis, while episomal vectors may still provoke inflammatory or cytotoxic responses. In addition, the production of clinical-grade viral vectors remains technically demanding and costly, posing scalability challenges. Capsid size constraints further limit the delivery of large or multi-component CRISPR cargos, such as Cas9 nucleases combined with regulatory elements or donor templates, an issue that is less pronounced in many non-viral nanoparticle-based systems^120^.

AAVs exemplifies the clinical maturity of viral delivery platforms. Originally identified as a contaminant in adenovirus preparations, AAV is a non-enveloped, non-pathogenic virus with a single-stranded DNA genome of approximately 4.7 kb flanked by inverted terminal repeats. Its favorable safety profile, capacity for long-term transgene expression without chromosomal integration, and the availability of naturally occurring and engineered serotypes with distinct tissue tropisms have established AAV as a dominant platform for *in vivo* gene delivery^123^. Multiple AAV-based therapies are now approved for clinical use, and more than 200 clinical trials are actively evaluating AAV-mediated interventions across a wide range of genetic disorders^124^. While systemic administration can be limited by pre-existing or therapy-induced neutralizing antibodies, localized delivery to immune-privileged tissues such as the eye or central nervous system can mitigate immune responses and enable redosing.

A central challenge for AAV-mediated CRISPR delivery is its limited packaging capacity, which complicates delivery of large nucleases such as *Streptococcus pyogenes* Cas9. Dual-vector strategies are therefore commonly employed, distributing Cas9 and guide RNA components across separate vectors. In addition to nuclease delivery, AAV vectors are widely used as donor templates for HDR and homology-independent targeted integration following CRISPR-induced DSBs, enabling precise genomic modifications including insertions, deletions, and point mutations^125^. Self-complementary AAV vectors further accelerate transgene expression by bypassing second-strand DNA synthesis, although this approach reduces effective packaging capacity by half^126^.

Lentiviral and adenoviral vectors further expand the viral delivery landscape. Lentiviral vectors, derived from human immunodeficiency virus type 1 (HIV-1), are enveloped RNA viruses capable of transducing both dividing and non-dividing cells and accommodating genetic payloads of approximately 8 kb. Adenoviral vectors, by contrast, are double-stranded DNA viruses characterized by broad tissue tropism, high transduction efficiency, and episomal persistence. Their large payload capacity and scalable manufacturing processes make them attractive for applications requiring delivery of complex genetic constructs, although high immunogenicity and pre-existing immunity to common serotypes limit their suitability for chronic gene therapy. The development of rare-serotype and helper-dependent adenoviral vectors, which lack viral coding sequences, has substantially reduced immune responses while enabling long-term expression of large transgenes^127^.

Collectively, viral delivery systems remain indispensable tools for *in vivo* CRISPR genome editing, offering unmatched efficiency and clinical maturity. However, their inherent trade-offs in immunogenicity, payload flexibility, and manufacturability underscore the growing interest in non-viral and hybrid delivery strategies.

### Adenoviruses (AdV)

Adenoviruses (AdVs) were first used as gene therapy vectors in the 1990s. Important for the development of gene therapies, they are scalable to produce and more than 50 times cheaper to produce in large quantities than AAVs^128^.The main advantage of AdVs compared to other viral vectors is their genome size, which allows for the delivery of large cargos. First-generation AdVs, with deletions in E1 and E3 (genes that are important for replication) have a packaging capacity of 8.5 kb, which is almost double that of AAVs. Development of third-generation, high-capacity AdVs (HCAdVs) further increased cargo capacity to 36 kb by removing all viral genes^129^. Large cargo capacity is crucial for the delivery of CRISPR/Cas payloads. Unlike AAVs, AdVs can easily package Cas together with its regulatory regions, multiple sgRNA cassettes, additional reporters, and more complex expression systems, such as inducible promoters. This enables a single viral vector to transduce a cell and achieve effective modification, increasing transduction efficiency compared to the two-virus systems often used with AAVs^130^.

A crucial aspect of gene therapy vectors is their tissue specificity. AdV tissue targeting and internalization depend on its capsid proteins—fiber, penton, and hexon. AdV5, the most commonly used naturally occurring serotype, exhibit strong liver tropism. The CAR receptor, which binds AdV5 fiber protein, and integrins, which bind its penton base, are differentially expressed across cell types, strongly in hepatocytes but weakly in many others, such as blood cells^131^. *In vivo*, liver targeting is further strengthened by hexon binding to coagulation factor X in the blood, which direct AdVs to the liver^132^. Together, this leads to a strong immune response against the vector, severe liver damage, and weak targeting of many other tissues. Designing and discovering AdVs with altered tropism and improved toxicity profiles is therefore critical for developing vectors suitable for *in vivo* applications. An important step toward safer adenoviral vectors was the adoption of HCAdVs. They contain no viral genes, resulting in lower immunogenicity and they were successfully used *in vivo* without triggering liver toxicity^133^. Furthermore, AdV serotypes other than AdV5 exhibit different tropisms and therefore show potential for targeting a variety of tissues. The use of chimeric serotypes and pseudotyped AdVs can further expand tissue targeting capabilities, potentially enabling delivery to the majority of tissues^134^. Another issue facing broader AdV adoption for gene therapy is preexisting immunity, as 90% of humans produce antibodies against AdV5, decreasing its effectiveness. Low-prevalence serotypes^135^ or pseudotypes that replace immunogenic capsid regions with less prevalent variants^136^ show promise in evading preexisting immunity.

Overall, as CRISPR-Cas systems become more complex, the ability of vectors to package large cargo and deliver it efficiently and cost-effectively to diverse tissues is becoming increasingly important. AdVs can carry large payloads but face significant drawbacks related to immunogenicity, toxicity, and tropism. However, the discovery and development of novel serotypes and capsid modifications show promise for improving safety and targeting capabilities, supporting their potential as gene therapy delivery platforms.

### Adeno-associated Viruses (AAV)

The precision of CRISPR-based genome editing has created major opportunities for the treatment of genetic diseases; however, efficient and safe *in vivo* delivery remains a central challenge. AAVs have emerged as the leading delivery platform due to their relatively low immunogenicity, capacity for long-term transgene expression, and availability of natural and engineered serotypes with defined tissue tropisms. These properties have enabled targeted genome editing across multiple organ systems and positioned AAVs at the forefront of *in vivo* therapeutic genome modification^32^.

Despite this promise, clinical translation of AAV-mediated CRISPR editing has progressed cautiously. To date, EDIT-101 represents the first and only *in vivo* AAV-CRISPR therapy to advance into clinical trials. EDIT-101 targets Leber congenital amaurosis type 10 (LCA10), caused by a pathogenic intronic mutation in *CEP290*, using AAV5-mediated delivery of SaCas9 and dual guide RNAs under the control of the photoreceptor-specific GRK1 promoter via subretinal injection^137^. Preclinical studies in rodents and non-human primates demonstrated efficient excision of the mutant intronic sequence, restoration of *CEP290* expression, and absence of detectable off-target editing or systemic immune toxicity. These findings supported initiation of the Phase I/II BRILLIANCE trial (NCT03872479), in which subretinal administration of EDIT-101 was well tolerated in 14 patients without dose-limiting toxicities or clinically significant immune responses, consistent with the immune-privileged nature of the eye^138^. Clinically meaningful improvements in cone-mediated vision were observed in a subset of participants, while others maintained stable retinal function, establishing proof of concept for safe *in vivo* genome editing in humans.

Parallel translational efforts in non-human primates have further illuminated both the potential and the limitations of AAV-CRISPR approaches. Sin *et al*. described AAV8-mediated delivery of SpCas9 and dual guide RNAs targeting *VEGFA* in rhesus macaque retina as a strategy for durable suppression of pathological angiogenesis^139^. Although efficient on-target editing was achieved, all treated eyes developed dose-dependent retinal abnormalities, including subfoveal deposits and outer retinal disruption. These effects occurred in the absence of strong systemic immune responses, suggesting that prolonged or high-level Cas9 expression itself can induce local tissue toxicity.

Although AAVs have a low tendency for genomic integration when used as a gene augmentation strategy, several groups have showed that significant portion of AAV sequences were integrated into the host genome due to the CRISPR induced DSBs. Hanlon *et al.*^140^ showed that high levels of AAV integration, reaching up to 47%, were observed at Cas9-induced DSBs within therapeutically relevant genes in cultured murine neurons, as well as in mouse brain, muscle, and cochlea. Genome-wide mapping of AAV sequences in mouse brain revealed no general increase in vector integration, with enrichment detected specifically at the CRISPR/Cas9 target site. A high AAV integration rate near the CRISPR target site was also demonstrated by Singh *et al.*^141^. Similarly, Bazick *et al*. reported AAV integration in up to 89% of all edits detected^142^. To fully assess the potential risk of this serious and unwanted side effect, a systematic evaluation should be performed. Methods such as targeted long-read sequencing, junction PCR, whole-genome sequencing, or ddPCR should be employed to detect and quantify AAV integration events^143^. This should be recognized as a possible, and in some settings common, outcome of applications that utilize AAV vectors for genome editing.

To address the strict AAV packaging limit of approximately 4.7 kb, which constrains delivery of large base editors, split-intein strategies have been developed. Müller *et al*. reported a dual-AAV5 system encoding an adenine base editor split into two halves that reassemble through protein trans-splicing in transduced cells to correct the *ABCA4* c.5882G>A mutation underlying Stargardt disease^144^. Subretinal delivery in cynomolgus macaques resulted in exceptionally high on-target editing efficiencies, reaching up to 75% in cones and 85% in retinal pigment epithelium, with minimal bystander or off-target edits. Mild, transient ocular inflammation was observed but this resolved without systemic immune activation, supporting the translational feasibility of dual-AAV split-intein base editing in large-animal models.

While effective, dual-AAV approaches increase vector dose requirements and may heighten immune risks. To overcome these limitations, Davis *et al*. engineered compact adenine base editors that fit within a single AAV vector by combining minimized regulatory elements with smaller Cas9 orthologs such as SaCas9, Nme2, and CjCas9^145^. Single-AAV delivery achieved therapeutically relevant editing levels in murine liver and heart with indel frequencies below 1%. In preliminary non-human primate studies, systemic administration produced substantial editing of *PCSK9* and *ANGPTL3* in hepatocytes^73^, leading to marked reductions in circulating cholesterol and triglycerides, without evidence of liver pathology or widespread off-target editing. These results highlight single-AAV base editing as a potentially safer and more scalable approach for systemic *in vivo* genome modification.

Beyond base editing, AAV vectors have also enabled systemic delivery of CRISPR nucleases for the treatment of infectious diseases. Burdo *et al*. demonstrated intravenous AAV9 delivery of an all-in-one SaCas9 dual-guide construct designed to excise integrated simian immunodeficiency virus proviral DNA in antiretroviral therapy-suppressed rhesus macaques^146^. Broad biodistribution to known viral reservoirs was achieved, accompanied by molecular evidence of proviral excision and favorable short-term safety outcomes, supporting the feasibility of systemic AAV-CRISPR strategies targeting latent viral infections.

Most recently, the identification of ultracompact CRISPR nucleases has further expanded the therapeutic scope of AAV delivery. Rauch *et al*. reported the discovery of NanoCas, an approximately 400-amino-acid nuclease that enables single-AAV delivery to tissues previously inaccessible to conventional systems^147^. Systemic AAV-NanoCas administration in non-human primates targeting dystrophin splice sites achieved robust editing in skeletal and cardiac muscle with low off-target activity in non-target organs. This work addresses a longstanding barrier in the development of genome-editing therapies for neuromuscular disorders, although comprehensive immunological and long-term safety studies remain essential.

Collectively, these studies highlight the central role of AAV vectors in advancing *in vivo* genome editing. Strategic selection of AAV serotypes has proven critical for tissue targeting, with AAV5 exhibiting strong performance in subretinal delivery, AAV8 in hepatic applications, and AAV9 and engineered capsids enabling systemic and central nervous system transduction. Innovations in compact editors, split-intein systems, and vector engineering have substantially mitigated payload constraints and expanded the therapeutic repertoire of AAV-based CRISPR technologies. Continued refinement of vector design, expression control, and immunomodulatory strategies is expected to further accelerate the clinical translation of durable and precise *in vivo* genome correction.

### Lentiviral Vectors

Over the past three decades, retroviral vectors derived from γ-retroviruses and lentiviruses have been predominantly employed for *ex vivo* gene addition therapies, in which target cells are genetically modified outside the body and subsequently reinfused. This strategy has achieved durable clinical success in hematopoietic stem and progenitor cells (HSPCs) and T lymphocytes, underpinning curative treatments for primary immunodeficiencies and hematological malignancies, including chimeric antigen receptor T-cell (CAR-T) therapies^148^,^149^. Following the demonstration that CRISPR-Cas9 could function efficiently in human cells^150^, lentiviral vectors were rapidly adapted for genome-editing applications due to their high transduction efficiency, broad tropism, and extensive clinical safety record.

A major limitation of integrating lentiviral vectors for CRISPR delivery is the constitutive expression of Cas9 and guide RNA, which can exacerbate off-target editing and prolong nuclease exposure^151^. In addition, insertion of the lentiviral genome into host chromatin carries a residual risk of insertional mutagenesis through activation of proto-oncogenes near integration sites. To mitigate these risks for *in vivo* genome editing, two principal strategies have emerged: transient expression of CRISPR components while retaining lentiviral infectivity, and reduction or elimination of vector integration.

In one approach, Ling and colleagues developed an adapted lentiviral system in which Cas9 is delivered as mRNA from an integrase-deficient lentiviral vector (IDLV), thereby limiting the duration of nuclease expression. Subretinal delivery of this vector encoding a guide RNA targeting murine *Vegfa* achieved up to 44% indel formation in a mouse model of wet age-related macular degeneration and reduced laser-induced choroidal neovascularization by 63%^152^. No detectable off-target editing or Cas9-specific immune responses were observed, likely reflecting transient Cas9 exposure and the immune-privileged nature of the ocular environment.

Complementary work by Ortinski and colleagues employed IDLVs expressing both Cas9 and guide RNA episomally to target the GABAA receptor α2 subunit in the nucleus accumbens of adult rats, a brain region implicated in major depressive disorders. Following intracranial delivery, the α2 protein became undetectable by western blot analysis 35 to 47 days after administration, accompanied by marked alterations in receptor subunit composition^153^. These studies represent the most compelling demonstrations of lentiviral vectors for *in vivo* genome editing, particularly in the eye and brain, where target cells are post-mitotic and episomal persistence is sufficient to sustain editing outcomes.

Notably, long-term consequences of persistent Cas9 and guide RNA expression in non-dividing cells remain incompletely understood. Tissue-specific adaptive immune responses to Cas9 have been reported, underscoring the need for careful immunological monitoring in such contexts^154^. In addition, both studies reported reduced viral titers relative to conventional lentiviral vectors, indicating that substantial optimization will be required to support therapeutic dosing for larger tissues or systemic administration. While lentiviral systems adapted for genome editing in actively dividing cells have been described, these efforts have largely focused on *ex vivo* applications and are reviewed elsewhere^155^.

More recently, *in vivo* targeting of bona fide HSPCs has been achieved using phagocytosis-shielded lentiviral vectors in mouse models of SCID-ADA, osteopetrosis, and Fanconi anemia, representing a major milestone toward systemic *in vivo* gene editing for hematological disorders^156^. This advance opens new avenues for lentiviral-based genome editing beyond localized or immune-privileged tissues.

### Virus-like Particles

In contrast to lentiviral vectors that encode genome-editing components within the viral genome, several groups have developed virus-derived particles that retain high infectivity while being entirely devoid of DNA cargo. These systems directly package Cas9 RNP complexes, enabling transient editing activity and minimizing risks associated with sustained transgene expression^157^.

The first such system, termed Nanoblades, was reported by Mangeot and colleagues in 2019. In this platform, Cas9 is fused to the C-terminus of the murine leukemia virus Gag protein, enabling RNP assembly within virus-like particles (VLPs) during production. Retro-orbital administration of Nanoblades targeting the murine *Hpd* gene yielded 7-13% indel formation in mouse liver at four weeks post-injection, without detectable toxicity or impact on survival^158^. This target had previously been validated for therapeutic rescue in hereditary tyrosinemia type I^159^.

Building on this concept, Banskota and colleagues developed engineered virus-like particles (eVLPs) capable of efficiently packaging base editors through systematic optimization of cargo incorporation, release, and intracellular localization^160^. eVLPs achieved therapeutically relevant levels of base editing in the liver and eye. In particular, disruption of a *Pcsk9* splice donor site via adenine base editing resulted in 63% editing in bulk liver tissue and a 78% reduction in serum PCSK9 levels, which is comparable to outcomes achieved with AAV- or LNP-based approaches^161^. Subsequent work extended this platform to prime editors, enabling correction of pathogenic mutations in mouse models of inherited blindness^162^.

Collectively, these studies establish VLPs as a powerful and flexible delivery modality for *in vivo* genome editing, capable of supporting precise nucleotide modification using base and prime editors and, in principle, addressing a broad spectrum of disease-causing mutations^16^. Nonetheless, further optimization is required. Use of broadly tropic envelopes such as VSV-G has resulted in measurable off-target editing in non-target tissues, highlighting the need for tissue-specific pseudotyping strategies^161^. Co-pseudotyping approaches have shown promise in *ex vivo* settings, and directed evolution strategies have recently been applied to identify eVLP variants with improved production characteristics and cell-type specificity^162^.

A remaining barrier to clinical translation is the limited availability of unbiased analytical tools for comprehensive characterization of increasingly complex virus-derived particles, particularly those carrying large effector fusions or multiple guide RNAs. As these platforms advance toward therapeutic application, robust orthogonal methods to assess critical quality attributes will be essential, as reviewed in detail by Dipalo and colleagues^163^.

## Organ-Specific Applications of* In Vivo* CRISPR Genome Editing

**Figure 3:**
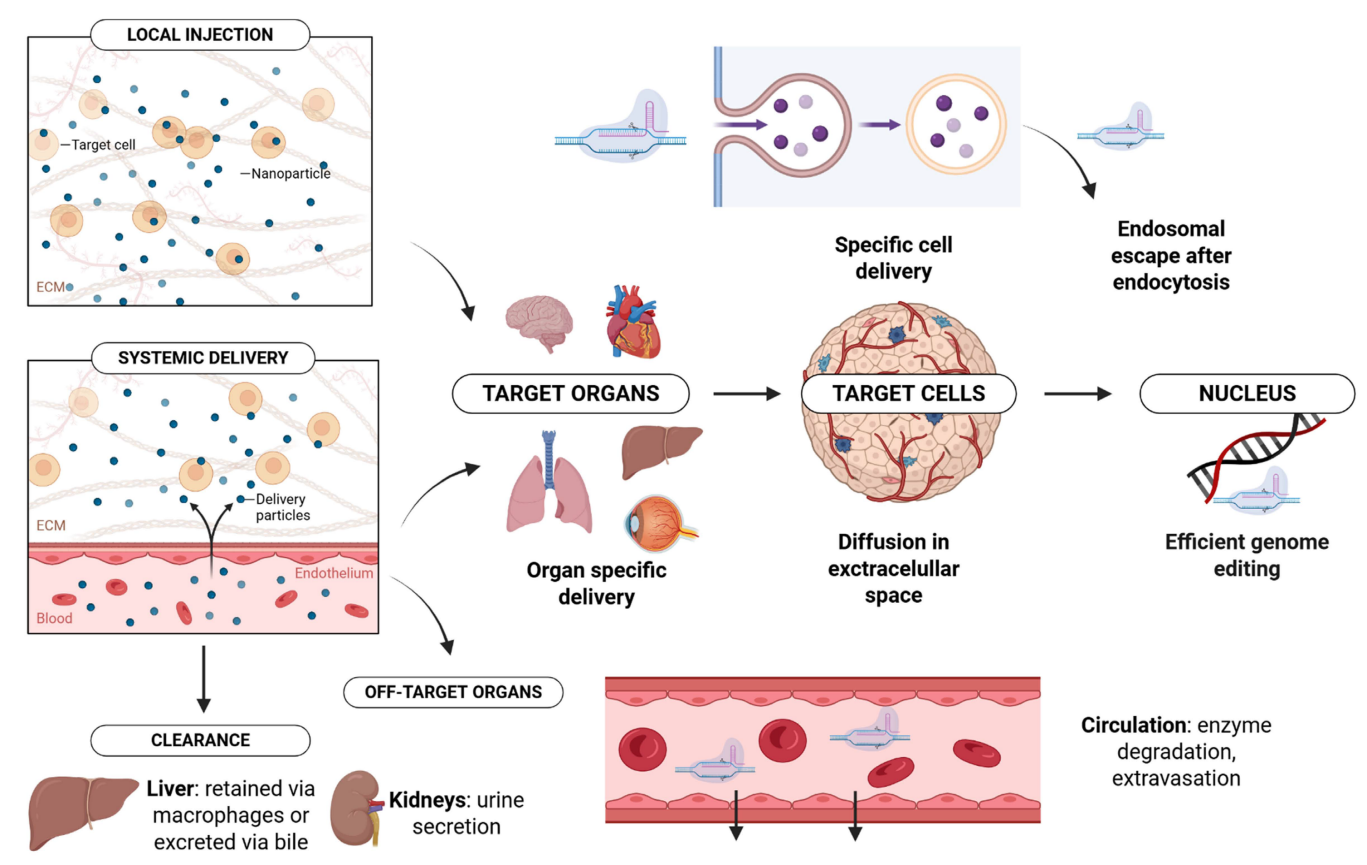
Delivery routes, biological barriers, and clearance pathways for *in vivo* genome editing. Schematic overview of local and systemic delivery strategies for genome-editing therapeutics and the key biological barriers influencing *in vivo* performance. Local injection enables direct deposition of delivery particles into the extracellular matrix (ECM) of target tissues, promoting uptake by nearby cells while limiting systemic exposure. In contrast, systemic delivery requires circulation through the bloodstream, extravasation across the vascular endothelium, and diffusion within target organs before cellular uptake. Targeting efficiency is shaped by organ-specific vascular permeability, extracellular transport, and cellular internalization mechanisms. Following endocytosis, efficient endosomal escape is essential to release the genome-editing cargo into the cytosol and allow subsequent nuclear entry for genome modification. During systemic administration, delivery vehicles may distribute to off-target organs and are subject to clearance, predominantly via hepatic sequestration by macrophages with biliary excretion or renal filtration. In addition, circulating genome-editing components can undergo enzymatic degradation and nonspecific interactions in the blood, further reducing bioavailability. Collectively, these delivery routes and biological barriers determine the biodistribution, editing efficiency, and safety of *in vivo* genome-editing therapies. Created in BioRender. Lainscek, D. (2026) https://BioRender.com/6mnqglf.

**Figure 4:**
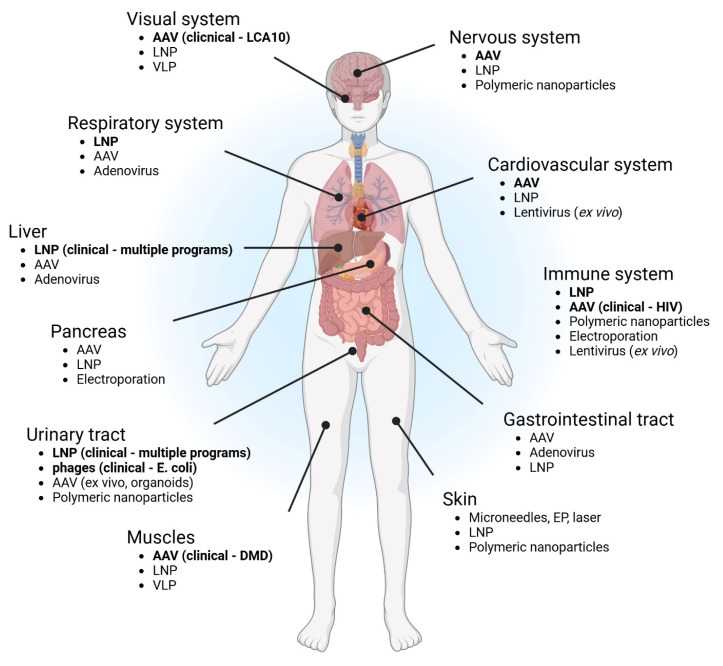
Delivery modalities for CRISPR/Cas-based genome editing across major organ systems. Schematic overview of the principal viral and non-viral delivery platforms currently explored for CRISPR/Cas genome-editing applications in different organ systems. The figure summarizes representative delivery modalities investigated in the visual, nervous, respiratory, cardiovascular, immune, gastrointestinal, hepatic, pancreatic, urinary, muscular, and cutaneous systems, including adeno-associated virus (AAV), lipid nanoparticles (LNPs), adenoviral vectors, lentiviral systems (primarily ex vivo), virus-like particles (VLPs), polymeric nanoparticles, electroporation, microneedle-based methods, and laser-assisted delivery approaches. The schematic highlights the organ-specific diversity of delivery strategies currently used in both experimental and clinically advancing genome-editing applications. Created in BioRender. Lainscek, D. (2026) https://BioRender.com/jme8tz7.

Because anatomical barriers (**Figure 3**), cellular turnover, immune surveillance, and delivery constraints vary substantially between organs, successful *in vivo* genome editing requires organ-tailored delivery strategies. Below, we provide an overview of the different delivery modalities used for CRISPR/Cas systems across selected organ systems (**Figure 4**). Unless otherwise stated, most examples discussed in this review have so far been demonstrated only in experimental laboratory settings, whereas clinically validated applications are explicitly indicated. The liver, retina, kidneys and muscles are the organ systems where *in vivo* delivery of CRISPR system has entered clinical trials. However, tremendous research into targeted delivery is still needed, and limitations in cargo capacity as well as potential immunotoxicities must also be addressed.

### Nervous System

The central nervous system, comprising the brain and spinal cord, together with the peripheral nervous system that enables peripheral innervation, is of exceptional interest for genome editing because of its unique biological constraints. Neurons are terminally differentiated and display minimal regenerative capacity; consequently, genetic alterations affecting the nervous system often result in lifelong and irreversible dysfunction.

More than one-sixth of monogenic diseases have a neurological origin, accounting for up to 40% of the clinical workload in pediatric hospital services^164^. To date, pathogenic variants in more than 1,700 genes have been linked to neurological disorders^165^, highlighting the substantial therapeutic potential of genome editing in this context. However, *in vivo* delivery to the nervous system is hindered by specialized anatomical barriers, most notably the BBB and the blood-cerebrospinal fluid (CSF) barrier. The blood-CSF barrier is formed by the epithelial cells of the choroid plexus and separates the CSF from systemic circulation. The BBB, in contrast, consists of tightly connected endothelial cells supported by astrocytic endfeet and leptomeningeal cells, creating a highly regulated interface that restricts molecular exchange with neural tissue. Although the blood-CSF barrier is relatively more permeable than the BBB, both structures significantly constrain access of genome-editing agents to the CNS^166^,^167^.

Beyond physical barriers, the nervous system presents molecular constraints that shape genome-editing strategies. The post-mitotic nature of neurons limits the applicability of HDR, while the immune-privileged yet inflammation-sensitive CNS microenvironment demands stringent control over editing precision and duration. Successful genome editing therefore requires careful selection of delivery vectors and administration routes that are sufficiently compact to cross the BBB or exploit CSF access, remain stable in circulation, and efficiently release cargo in target cells. Moreover, neurons rely on specialized, activity-dependent DNA repair pathways^168^,^169^, which should be considered when designing editing modalities.

AAV vectors have historically dominated CNS gene delivery because of their strong neuronal tropism and capacity for durable expression^170^. One of the earliest demonstrations of *in vivo* genome editing in the brain employed dual AAVs encoding SpCas9 and guide RNAs targeting *Mecp2*, achieving approximately 70% reduction in MeCP2 protein levels in the dentate gyrus following stereotaxic injection and establishing that precise gene disruption is feasible in mature neurons^171^. Subsequent engineering of capsids such as AAV-PHP.B and AAV-PHP.eB markedly enhanced neural transduction efficiency. Using these variants, near-complete biallelic editing was achieved in transduced neurons, resulting in widespread reductions in neuronal markers across the cortex, hippocampus, and spinal cord^170^. Comparative analyses of AAV9, AAV-PHP.B, and AAV-PHP.eB further demonstrated robust editing of neuronal and astrocytic markers, while oligodendrocytes remained comparatively resistant to editing^172^.

Base editing has added an additional layer of precision to CNS genome editing. Split-intein AAV systems delivering cytosine or adenine base editors enabled widespread editing following neonatal or systemic administration, with editing efficiencies strongly dependent on brain region and cell type^173^. In disease-relevant contexts, dual AAV9-mediated base editing of the *SMN2* gene achieved measurable editing in the brain and spinal cord in a spinal muscular atrophy model^174^. In contrast, homology-independent targeted integration strategies improved functional outcomes only when combined with gene supplementation, emphasizing that severe loss-of-function disorders may require combined editing and replacement approaches^175^. CRISPR-based transcriptional modulation has also been applied in the CNS, as demonstrated by inducible activation of *Kcna1* in epilepsy models, which ameliorated disease phenotypes following localized AAV delivery^176^.

Non-viral delivery systems, particularly nanoparticles, have gained increasing attention due to their reduced immunogenicity and chemical tunability. Strategies exploiting BBB transport mechanisms have enabled systemic delivery of genome-editing payloads to the brain, achieving modest but functionally relevant editing levels in neuronal targets^105^,^177^. Local delivery of biodegradable nanocapsules or polyplex micelles has further enabled efficient neuronal editing following stereotaxic injection, with cell-type specificity determined largely by injection site and nanoparticle design^178^,^179^. Amphiphilic and gold-based nanocomplexes have also demonstrated therapeutic efficacy in neurodegenerative disease models, reducing pathological protein accumulation and restoring behavioral phenotypes without detectable immune infiltration^25^.

Cell-penetrating peptides have emerged as an additional alternative to viral and nanoparticle-based delivery. Self-deliverable Cas9 RNPs fused to peptides derived from semaphorin-3A achieved low but measurable editing in striatal neurons following direct injection, accompanied by reductions in disease-relevant transcripts^180^. Although efficiencies remain modest, these systems highlight the potential of chemically defined delivery approaches in the CNS.

Developmental timing and route of administration remain decisive determinants of CNS transduction. *In utero* or neonatal delivery exploits the relative immaturity of the BBB and enables widespread neuronal access, as demonstrated by LNP-mediated editing in fetal brains and systemic AAV9 delivery in neonatal mice^181^,^182^. In contrast, equivalent systemic delivery in adults results in markedly restricted neuronal transduction, underscoring age as a biological barrier to CNS genome editing.

Beyond DNA-targeting nucleases, RNA-targeting CRISPR systems have enabled efficient and reversible modulation of gene expression in the nervous system. AAV-mediated delivery of Cas13 variants achieved robust silencing of disease-associated transcripts in spinal cord and brain, leading to functional improvements in neurodegenerative disease models^183^.

Although most successful *in vivo* editing studies in the adult nervous system rely on direct intracranial injection, this approach is highly invasive and limits transduction to localized regions^184^. Alternative, less invasive strategies are therefore under active investigation. Intranasal delivery of AAV-based CRISPR components has achieved substantial gene disruption and behavioral rescue in anxiety models^185^, while focused ultrasound-mediated opening of the BBB has been shown to enhance systemic AAV-mediated editing in neural tissue^186^.

Non-human primate studies have provided critical validation of translational relevance. AAV-mediated knockout of *MECP2* in adolescent rhesus monkeys reproducibly induced autism- and Rett-like phenotypes, demonstrating that *in vivo* genome editing can modulate complex neurological traits in large-brain primates^187^.

Collectively, these studies indicate that AAV vectors remain the most established platform for long-term and cell-type-specific genome editing in the nervous system, while non-viral systems are rapidly advancing and narrowing the efficiency gap. Reported neuronal editing efficiencies range from approximately 1% to 60%, depending on delivery route, developmental stage, and target cell population. Future progress will depend on the development of less invasive delivery strategies capable of achieving broad and uniform CNS distribution, thereby enabling clinically viable genome-editing therapies for neurological disease. Despite significant progress, considerable research is still needed to achieve efficient and safe delivery of genome editors to the CNS. In parallel, novel compact Cas9 variants are being engineered to facilitate packaging into AAV vectors, while potent brain-targeting motifs for non-viral delivery modalities are being actively investigated.

### Cardiovascular System

Cardiovascular diseases (CVDs) remain the leading cause of morbidity and mortality worldwide despite major advances in preventive pharmacotherapy and interventional cardiology. Even with intensive lipid lowering (statins and PCSK9 inhibitors), antithrombotic therapy, and guideline-directed heart failure regimens, substantial residual risk persists. In parallel, many inherited cardiomyopathies and arrhythmia syndromes still lack causal, mutation-directed treatments. These gaps have strengthened interest in gene-based interventions capable of producing durable, disease modification following a potentially one-time treatment^188^.

Genome editing technologies are particularly attractive in cardiometabolic medicine because they can either inactivate pathogenic pathways, such as hepatic regulators of atherogenic lipoproteins, or precisely correct disease-causing variants. In cardiovascular research, this framework has enabled disruption of genes involved in lipid metabolism (including *PCSK9*, *ANGPTL3*, and *APOB*), exploration of correction strategies for inherited cardiomyopathies, and modulation of inflammatory or fibrotic pathways after myocardial injury. Translation has accelerated through non-human primate studies demonstrating durable hepatic gene silencing and early-stage clinical programs pursuing “once-and-done” lipid lowering via gene disruption or base editing, underscoring the importance of safe, efficient, and tissue-selective *in vivo* delivery for therapeutic impact^73^,^74^.

Several leading cardiometabolic development programs, already in clinical trials, use adenine base editors to introduce protective loss-of-function variants in hepatic lipid genes, while prime editing provides a conceptual route to mutation-specific correction for monogenic cardiomyopathies and channelopathies^189^-^191^.

The clinical feasibility of cardiovascular genome editing is fundamentally constrained by delivery to relevant tissues. AAV is the most established platform for *in vivo* gene transfer to cardiac and hepatic tissues, with cardiomyotropic serotypes such as AAV9 and AAVrh74^192^ supporting cardiomyocyte transduction and AAV8 and engineered capsids showing strong hepatotropism. Lentiviral vectors are central for *ex vivo* modification of hematopoietic stem cells and engineered cell therapies, but their integration profile raises concerns for direct *in vivo* cardiovascular use, particularly in non-renewing myocardium^193^.

Non-viral delivery platforms, particularly LNPs, have rapidly become the leading clinical modality for *in vivo* genome editing. In animal studies and non-human primate studies, liver-directed editing of *PCSK9* has produced durable suppression and substantial LDL-cholesterol reductions after a single dose, supporting progression to human testing. Early clinical studies have also reported dose-dependent reductions in lipid targets after LNP-mediated editing of *ANGPTL3*. A related liver-directed strategy has demonstrated clinical translation for transthyretin (*TTR*) disruption in transthyretin amyloidosis, including patients with cardiomyopathy. At the same time, current LNP chemistries remain inefficient for direct myocardium or vasculature targeting and can trigger infusion reactions, complement activation, and transient hepatotoxicity, motivating efforts to engineer cardio-tropic nanoparticles through modified ionizable lipids, tissue-targeting ligands, and regional delivery approaches such as intracoronary administration^74^,^161^.

Clinically, the most advanced CRISPR programs relevant to cardiovascular medicine focus on liver-directed editing for lipid disorders and amyloidosis, representing a shift from chronic pharmacotherapy toward single-intervention genomic risk modification. Overall, while no CRISPR therapy is approved specifically for a cardiovascular indication, ongoing liver-directed programs and rapid progress in delivery engineering suggest that near-term cardiovascular impact will likely come from hepatocyte-targeted cardiometabolic risk reduction, followed by longer-term expansion into heart- and vessel-directed editing as cardio-selective delivery systems mature^194^.

### Immune System

The immune system presents a particularly demanding context for *in vivo* genome editing because it combines extreme cellular diversity with rapid turnover and broad anatomic distribution. While *ex vivo* editing has already produced major clinical impact, including engineered T cell therapies and hematopoietic stem and progenitor cell (HSPC) correction strategies, direct *in vivo* modification of immune cells relevant to clinics remains an emerging frontier with the potential to transform treatment paradigms for cancer, autoimmune disease, infectious diseases, and congenital immunodeficiencies. However, clinical translation has so far been limited to *ex vivo* genome modification approaches. Achieving this *in vivo* requires delivery systems that can reach mobile, short-lived cells, penetrate lymphoid tissues protected by structural and endothelial barriers (bone marrow, spleen, thymus, and lymph nodes), and avoid triggering innate nucleic acid sensing pathways that respond to exogenous RNA, DNA, or protein cargos. A further challenge is cell-type specificity, since selectively targeting T cells, B cells, or myeloid compartments within a dynamic immune environment demands precise tropism and tight temporal control. These constraints have driven the development of both viral and non-viral delivery strategies, each with distinct trade-offs in efficiency, payload capacity, immunogenicity, and redosing feasibility^33^,^195^. Among viral approaches, AAV vectors are frequently considered because of their favorable safety profile and extensive clinical experience. Beyond the CNS, AAV9 and related serotypes have also been evaluated for hematopoietic and immune cell transduction, including subsets of lymphoid and myeloid lineages, but reported efficiencies are variable and context-dependent, and immune recognition can constrain repeat dosing. These limitations, together with the need for transient editor exposure in many immunology applications, have accelerated interest in non-viral systems^195^.Non-viral platforms, especially LNPs, are increasingly prioritized for *in vivo* immune editing because they can deliver RNPs or mRNA without genome integration and can be engineered for transient expression kinetics. In addition to formulation-driven organ tropism, LNPs can be functionalized with antibodies or ligands to bias uptake toward defined immune subsets, including T cells (for example via CD3 or CD4) and B cells (for example via CD19), enabling selective editing in preclinical models. Engineered EVs and cell-derived nanoparticles add complementary options by leveraging endogenous biocompatibility and, in some contexts, improved immune evasion, although scalable manufacturing, cargo loading uniformity, and biodistribution control remain active areas of development. Collectively, these modalities have enabled early proof-of-concept studies in specific immune populations, most prominently T cells and HSCs, and more recently antigen-presenting cell compartments such as dendritic cells, thereby providing a foundation for translational immunotherapies^196^-^198^.

T lymphocytes are central effectors in antitumor immunity and antiviral responses and are also key drivers of autoimmune pathology, making them a high-value target for therapeutic genome editing. *In vivo* T cell editing could bypass the logistical complexity and manufacturing constraints of *ex vivo* workflows, but T cells are intrinsically difficult to transfect and are widely distributed, necessitating delivery systems that combine efficient uptake with acceptable safety profiles. Recent work has highlighted that LNP performance in T cells is highly formulation-dependent. Screening of synthetic lipidoid libraries identified imidazole-containing variants as particularly effective^81^. Building on these concepts, Lu *et al*. developed a multistep screening platform to identify a spleen-tropic LNP formulation that preferentially delivers CRISPR-Cas9 proteins to splenic T cells in Ai9 mice, enabling *in vivo* knockout of *CCR5* and *PD*-1 and establishing a route toward functional immune reprogramming for HIV resistance and cancer immunotherapy^199^. In parallel, localized physical delivery concepts continue to evolve as an alternative to systemic nanoparticles. Conductive hydrogel-based electroporation (hydro-EP) integrates a biocompatible hydrogel matrix with controlled electrical pulses to enable localized nucleic acid delivery into primary T lymphocytes, allowing spatial control over exposure and potentially reducing off-target distribution. As a proof of concept, hydro-EP-mediated delivery of CRISPR-Cas9 DNA reduced PD-1 expression and enhanced antitumor activity *in vivo*. However, the requirement for tissue access and local field control poses scalability challenges relative to systemically dosed LNPs, which offer more readily tunable pharmacokinetics and whole-body distribution profiles^200^. Recently Nyberg *et al.* published a study describing a major advance in *in vivo* T-cell engineering, demonstrating site-specific CRISPR/Cas9 mediated integration of large DNA payloads directly into primary human T cells *in vivo*. Using a dual-delivery system consisting of CD3-targeted enveloped delivery vehicles (EDVs) for Cas9 RNPs and an optimized AAV donor vector carrying the CAR template, the authors achieved precise integration of a promoterless CAR transgene into the TRAC locus in humanized mouse models. This resulted in therapeutic levels of *in vivo*-generated CAR-T cells capable of controlling hematological malignancies and solid tumors, representing a significant step toward off-the-shelf, manufacturing-free cell therapies^201^.Hematopoietic stem cells (HSCs) are uniquely valuable targets because durable editing at the stem cell level can repopulate the hematopoietic system and enable potentially curative therapies for monogenic blood disorders. Direct *in vivo* editing of HSCs could reduce dependence on *ex vivo* manipulation and cytotoxic conditioning regimens, but delivery into the bone marrow niche is constrained by biodistribution and local microenvironment barriers. Breda *et al*. demonstrated that CD117 (c-Kit)-targeted LNPs can deliver mRNA cargos to long-term HSCs *in vivo*, including mRNAs encoding base editors. In the context of sickle cell disease, this strategy achieved high rates of adenine base editing at the pathogenic beta-globin mutation in patient-derived HSCs, restored erythroid differentiation, and substantially reduced sickling, illustrating how receptor-targeted LNP delivery can enable functional, durable genome modification *in situ*^197^. Developmental delivery windows provide another route to overcome access barriers. Palanki *et al*. developed a CD45-targeted LNP platform designed to deliver CRISPR cargos to fetal HSCs via the liver, which functions as an accessible hematopoietic niche during development. Following a single *in utero* injection, the platform achieved potent and durable gene modulation, supporting proof of concept for prenatal intervention in monogenic hematologic disorders and highlighting how receptor-targeted nanoparticles can be optimized for challenging stem cell compartments^198^. Industry efforts also underscore the translational momentum of *in vivo* HSC editing. Tessera Therapeutics reported preclinical data describing LNP-mediated delivery of RNA Gene Writers™ to HSCs and T cells in humanized mouse models, with substantial and durable rewriting efficiencies in long-term HSCs after a single intravenous administration. While these findings require peer-reviewed publication and independent validation, they illustrate active platform development focused on scalable *in vivo* immune and stem cell targeting^202^. Complementing antibody-targeted strategies, antibody-free LNP approaches are also progressing toward robust HSC delivery. Optimized LNPs delivering ABE8e/sgRNA mRNA enabled efficient base editing of the HBG1/2 promoter in human HSCs and reactivation of fetal hemoglobin in erythroid progeny, including in transfusion-dependent beta-thalassemia patient-derived HSCs engrafted in immunodeficient mice. These results support the feasibility of single-dose, non-viral editor delivery to endogenous loci in human HSCs without *ex vivo* manipulation or mobilization, reinforcing a plausible path toward broadly scalable hemoglobinopathy therapies^203^.

Dendritic cells (DCs) coordinate adaptive immunity through antigen processing and presentation and are therefore attractive targets for immuno-oncology and immune tolerance engineering. Recent studies have demonstrated the feasibility of *in vivo* genome editing in DCs to modulate checkpoint pathways and enhance antitumor responses. A lipid-assisted nanoparticle platform (BLANs) was used to interrogate how cholesterol density influences uptake, endosomal escape, and cargo release in DCs, and low-cholesterol BLAN formulations improved delivery performance and enabled efficient PD-L1 knockout in conventional DCs. Functionally, PD-L1 disruption enhanced cDC1 activation, promoted T cell stimulation, and suppressed tumor growth *in vivo*, highlighting DC editing as a direct strategy to reshape tumor-immune interactions^204^. Tumor-microenvironment responsive materials provide an additional strategy by coupling delivery performance to local biochemical conditions. Hollow mesoporous manganese dioxide nanoparticles have been used to deliver CRISPR-Cas9 RNPs to DCs within tumors, targeting pathways such as MTH1 to amplify oxidative stress, promote DC maturation, and strengthen innate and adaptive antitumor immunity. Together, these approaches illustrate that *in vivo* DC engineering can be achieved through multiple material architectures, but continued work is needed to define optimal targeting, safety, and manufacturing profiles suitable for translation^205^.

Across T cells, HSCs, and DCs, the central translational priorities remain consistent: improving cell-type specificity in complex tissues, achieving therapeutically meaningful editing in relevant niches, minimizing innate immune activation, and establishing scalable manufacturing and quality control for increasingly sophisticated cargos^197^,^204^.

### Gastrointestinal System

The gastrointestinal (GI) tract represents one of the most complex environments for *in vivo* genome editing because it combines harsh physicochemical conditions with rapid cellular turnover, dense microbial ecosystems, and pronounced spatial compartmentalization. At the same time, it offers unique therapeutic opportunities, as the gut epithelium is continuously renewed from stem cells residing in the crypts of Lieberkühn, raising the possibility that successful editing of these progenitors could lead to durable tissue modification. Oral administration is particularly attractive due to its non-invasive nature, high patient compliance, and the large absorptive surface of the intestine. However, delivery of CRISPR-based systems to GI tissues must overcome multiple barriers, including acidic pH, digestive enzymes, thick mucus layers, rapid epithelial shedding, and competition from commensal microbes, while also achieving cell-type specificity among enterocytes, stem cells, immune cells, stromal cells, or distinct microbial strains. Collectively, these constraints make efficient intracellular delivery and nuclear localization of large Cas9 or dCas9 complexes especially challenging, and they place stringent demands on delivery vehicle stability, specificity, and scalability^206^,^207^. To address these challenges, a diverse set of delivery strategies has emerged, broadly divided between approaches targeting the gut microbiome and those aimed at host tissues. For microbial genome editing, engineered conjugative plasmids and bacteriophage-based vectors leverage natural bacterial gene-transfer and infection pathways to deliver CRISPR cargos with high strain specificity. These systems can selectively edit or eliminate defined bacterial populations while sparing the surrounding microbiota, offering a level of precision that is difficult to achieve with conventional antibiotics. In parallel, host-directed delivery approaches include viral vectors such as AAV or adenovirus optimized for epithelial or immune-cell tropism, non-viral LNPs engineered for mucosal stability or oral administration, and local delivery routes such as endoscopic administration. *Ex vivo* edited intestinal organoids or immune cells followed by transplantation represent an additional strategy to bypass luminal barriers altogether. Recent reviews emphasize rapid progress in non-viral carrier platforms, particularly lipid-based systems, that are expanding *in vivo* editing beyond the liver and toward mucosal tissues^208^-^210^.

Preclinical studies in GI-relevant contexts have so far advanced most rapidly in microbiome editing. Phage-delivered CRISPR systems have been successfully used to selectively eliminate multidrug-resistant bacteria in the gut of animal models, demonstrating that *in vivo* microbial editing can achieve both specificity and functional benefit without broadly disrupting commensal communities. Host-cell editing in the GI tract remains less mature but is progressing conceptually and technically. Intestinal organoid systems have become a robust platform for modeling disease and testing editing strategies, with reports of extremely high knockout efficiencies for tumor suppressor genes such as *PTEN*. Building on this foundation, colon-targeted LNPs with enhanced mRNA delivery to inflamed colonic epithelium have recently been described, suggesting a path toward direct *in vivo* epithelial editing in inflammatory bowel disease or colorectal cancer. In oncology, CRISPR-Cas9 is already widely used *in vitro* and *in vivo* to model driver mutations and validate therapeutic targets across GI malignancies, including colorectal, gastric, esophageal, pancreatic, and hepatocellular cancers^211^,^212^.

Despite these advances, significant translational and regulatory challenges remain. Off-target editing and genotoxicity are of particular concern in highly proliferative tissues such as intestinal crypts, where unintended mutations could clonally expand. Immune responses against Cas proteins or viral vectors may limit repeat dosing or provoke inflammation in an already immune-reactive mucosal environment. In the context of microbiome editing, additional ecological risks must be considered, including horizontal gene transfer and unintended dysbiosis that could have long-term consequences for host health. Finally, scalable manufacturing of delivery platforms that are robust, well-characterized, and compatible with oral or local GI administration remains an unresolved bottleneck. Addressing these issues will require systematic evaluation of delivery efficiency, immunogenicity, off-target effects, and ecological impact to enable safe translation of genome editing technologies to GI diseases^213^-^215^.

### Liver

Liver-directed gene editing has rapidly moved from promising preclinical models to generating significant positive data in already ongoing clinical trials. Across multiple industry-led programs, LNPs are being employed for the delivery of genome-editing systems targeting *TTR*, *KLKB1*, *ANGPTL3*, *LPA*, and *PCSK9*. The liver's high accessibility, large size, and role in numerous metabolic disorders make it a prime target for *in vivo* genetic therapies, primarily leveraging CRISPR-Cas technology and LNP delivery. However, viral vectors, such as AAV and AdV, have been also employed in versatile preclinical settings due to their high hepatic tropism and efficient gene expression capacity. In this section we will summarize the main preclinical and clinical data on gene editing for liver diseases^216^. Early studies employed nuclease-free HDR using AAV donor templates to integrate therapeutic cDNAs into highly expressed loci such as Alb (Albumin) and ApoA1 (Apolipoprotein A1). Although editing efficiencies were modest (1-3% in adult mice; up to ~10% in neonates), these strategies achieved therapeutic benefit in models of Hemophilia B^217^, AATD (Alpha-1 Antitrypsin Deficiency)^218^, CN (Crigler-Najjar) syndrome, MMA (Methylmalonic Acidemia)^219^, WD (Wilson Disease), and HT1 (Hereditary Tyrosinemia Type I)^220^. To overcome low HDR efficiency, investigators incorporated programmable nuclease, ZFNs and CRISPR/Cas9, alongside AAV donor templates, increasing editing to ~10% of hepatocytes and enabling durable protein replacement. This strategy demonstrated efficacy in Hemophilia A/B^217^, MPS I/II (Mucopolysaccharidoses Types I and II)^221^, Fabry disease^222^, OTCD (Ornithine Transcarbamylase Deficiency)^223^ and HT1^224^. Dual-AAV CRISPR platforms achieved high expression in neonates but showed reduced potency in adults due to still lower recombination efficiency^225^. Precise HDR-mediated correction remains limited by low efficiency and mutation-specific design requirements. Nonetheless, proof-of-concept success has been demonstrated in AATD^226^, familial hypercholesterolemia^227^, OTCD^228^, GSD Ia (Glycogen storage disease type Ia)^229^ and PKU (Phenylketonuria)^230^. Improved vectors such as all-in-one AAVs, self-linearizing donor templates, and AAV-LNP hybrid systems—are under active development to increase efficiency^231^. Targeting hypercholesterolemia via *PCSK9* or *ANGPTL3* knockout has demonstrated extraordinary efficiency in reducing cholesterol levels in both small and large animal models with significant therapeutic benefit^232^. Finally, PH1 (Primary Hyperoxaluria Type 1), a disease characterized by oxalate accumulation in the kidneys, was addressed by inactivating the genes *Hao1* and *Ldha*, which encode enzymes acting upstream of the mutated gene. Experimental data showed a significant reduction in oxalate accumulation, and clinical trials are scheduled to begin in 2026^233^^,^^234^. As described, CBEs and ABEs, enable efficient, DSB-free single-nucleotide conversion. BEs have corrected pathogenic alleles in AATD, PKU, PH1, HT1, and MPS I, using diverse delivery system such as AdV, dual AAV systems or VLPs^235^. For examples, ABE has been used to treat AATD correcting the disease-causing mutation p.Glu342Lys resulting in the elevation of serum AAT levels and the clearance of AAT aggregates in the liver, most likely due to the selective advantage of corrected hepatocytes^236^. In PKU mouse models, transient expression of CBE or ABE of LNPs led to efficient on-target editing and to the reversal of the disease phenotype^237^. Moreover, BEs have been effectively used to silence *PCSK9* and *ANGPTL3* in rodents and non-human primates (NHPs) for the treatment of hypercholesterolemia. Although prime editing has been used effectively to correct pathogenic mutations in both *in vitro* and *ex vivo* settings, its considerable molecular size poses obstacles for *in vivo* deployment. Several delivery approaches have been tested in mouse models, including dual AAVs, gutless AdV vectors, hydrodynamic tail-vein injection of plasmids, and LNPs^238^^,^^239^^,^^240^. For example, the Fah mutation responsible for HT1 was repaired using either dual AAV vectors or hydrodynamic injection of plasmids carrying the prime-editing machinery^241^. In addition, a dual-AAV platform adapted to SaCas9 was used to correct the AAT mutant allele in a model of AATD, allowing the entire prime-editing system to fit within two AAV vectors^242^. HDAd vectors, which can accommodate all required components in a single construct, have produced robust editing in neonatal PKU mice^243^ as have LNPs^244^. The application of epigenome editing to inherited liver disorders remains limited. Notably, recent *in vitro* screening demonstrated that zinc-finger proteins outperform other DNA-binding domains for silencing the mouse *Pcsk9* gene. *In vivo*, a single LNP dose delivering mRNAs encoding these editors reduced circulating PCSK9 levels by nearly 50% for up to one year^245^. Strikingly, this persistent Pcsk9 repression was maintained even after experimentally induced liver regeneration, offering compelling evidence for the durability and heritability of the epigenetic modifications. Thus, liver-directed gene editing is rapidly evolving from proof-of-concept studies to therapeutic reality. CRISPR knockout therapies currently lead the field, while base and prime editing continue to advance despite outstanding challenges related to delivery and safety. Expanding applications, including antiviral genome editing and bespoke genetic interventions, position the liver as the central testing ground for transformative *in vivo* genetic therapies. Continued progress in delivery technologies, editing precision, and long-term safety will define the next generation of liver-targeted gene-editing treatments.

### Pancreas

Over the past decade, CRISPR/Cas9 has been applied to the pancreas primarily as a tool for somatic gene editing to model disease and to test therapeutic strategies. Early laboratory studies established practical *in vivo* pancreas editing by direct intra-pancreatic delivery (electroporation, plasmid transfection, and AAV) to generate multiplexed tumor models and to knock out oncogenes or tumor suppressors in adult mice, demonstrating the feasibility of focal somatic editing of ductal and acinar compartments^246^. In summary, the mosaic, low-frequency transfection achieved with CRISPR/Cas9 reliably reflects the stochastic behavior of human tumorigenesis, underscoring its value in biological and preclinical research contexts. Furthermore, by performing somatic CRISPR/Cas9-based mutagenesis screens of 125 commonly mutated genes in pancreatic cancer, researchers identified *USP15* and *SCAF1* as key tumor suppressors. These results underscore how *in vivo* CRISPR screening can effectively integrate human cancer genomic data with mouse models to identify driver genes that may inform prognosis and therapy^247^. In parallel, preclinical efforts shifted towards clinically relevant delivery modalities: AAVs remain useful for persistent nuclease expression and CRISPR screens in pancreatic cancer^248^, while LNP formulations and ionizable lipids have recently demonstrated the ability to deliver mRNA/CRISPR cargo to pancreatic islets (especially β-cells) after intraperitoneal or targeted systemic dosing, improving transient delivery and reducing long-term nuclease exposure, which raises safety concerns^249^. These studies also highlighted recurring limitations: adult pancreatic β-cells are largely post-mitotic, so HDR is inefficient, off-target effects and immune responses to Cas9 or viral capsids remain barriers and achieving cell-type specificity (pancreatic β vs α vs pancreatic ductal cells) at a therapeutic scale is still unresolved^250^. Complementary work using dCas9 fused to transcriptional or epigenetic effectors (activators such as VP64/P300, KRAB repressor, TET1/DNMT3A for site-specific demethylation/methylation) has progressed from *in vitro* demonstrations (silencing lineage factors such as *Arx* in α to β trans differentiation protocols^251^ to *ex vivo* islet reprogramming and small *in vivo* proof-of-concepts aimed at restoring or reprogramming endocrine function. Multiplexed dCas9 epigenetic systems have been used to activate β-cell gene networks including the (human pancreatic beta-cell genes, *PDX1*, *NEUROG3*, *PAX4* and *INS*) and to modulate chromatin at regulatory elements without introducing DSBs, representing an attractive safety trade-off for diabetes applications^252^. Delivery for epigenetic editing has largely followed the same approach as nuclease methods (AAV/viral vectors for durable expression, LNPs and mRNA for transient, lower-risk dosing), with growing interest in modular, transient RNP or mRNA+dCas9-effector combinations to limit ectopic activity. Reviews and recent preclinical reports emphasise that the field's critical next steps are improving pancreas/islet tropism and cell-type specificity of non-viral carriers, quantifying the durability and reversibility of epigenetic edits and rigorous evaluation of immunogenicity and off-target chromatin effects before considering clinical translation.

### Muscles

Muscular dystrophies (MD) are the most common muscular genetic diseases with the global prevalence of 16.14 per 100,000 people. Current treatments only mitigate the symptoms and aim to reduce the progression of the disease rather than addressing the underlying cause. With the utilization of CRISPR editing systems the mutations could be corrected, restoring the muscles functionally^253^. The most prevalent MD, Duchenne muscular dystrophy (DMD) typically arises via mutations that disrupt the reading frame of the dystrophin encoding gene, which is the largest human gene, creating premature translation termination^254^. By converting the out-of-frame mutations into in-frame mutations through exon skipping, the severity of DMD can be reduced, resulting in a phenotype similar to the milder Becker muscular dystrophy (BMD). Although this can be achieved using antisense oligonucleotides (AO), their transient effects require repeated administration. Furthermore, the current *in vivo* efficiency of AO-mediated exon skipping is low due to delivery limitations^255^. In contrast to AOs, single administration of CRISPR systems could provide permanent genome editing and dystrophin gene restoration. The first proof-of-concept study was performed in 2014, which used the mdx mouse (C57BL/10ScSn-Dmdmdx/J) model system that bears a nonsense mutation in exon 23 of the DMD gene. Germline exon correction was performed using HDR. The sgRNA, SpCas9, and the ssODN HDR template were injected directly into mouse zygotes. A 41% gene correction was achieved, restoring dystrophin expression in the majority of muscle tissues^256^. In terms of *in vivo* delivery, the predominant approach involves systemic administration of AAV vectors due to their skeletomuscular tropism, low immunogenicity, and high transduction efficiency^257^. However, their application is constrained by their limited cargo packaging capacity. Consequently, smaller Cas enzymes or dual-AAV delivery are often utilized^258^. To date, the only clinically translated approach for muscle disorders is AAV-delivered Cas12-mediated exon reframing of DMD.

The latter is associated with a risk of immunogenicity due to higher viral doses required. To overcome the current limitations related to packaging capacity and immunogenicity, non-viral delivery system such as LNPs, extracellular vesicles, and VLPs have been explored^259^. However, their efficiency and targeting are much lower than viral vectors. Preclinical studies in mdx mice, showed restored dystrophin expression after the administration of rAAV8 encapsulating dual sgRNA and Cas9 enzyme. Following murine experiments, exon deletion was presented in larger animals: exon 52 deleted pigs and exon 50 deleted canine as well^260^. Novel alternative strategies include base= and prime editing. These provide precise, robust correction without any DSB. Base editors are capable of changing a single base pair to correct point mutations. In 2024, ABE was intramuscularly administered in a humanized mouse model, restoring dystrophin expression in 59% of the skeletal muscle fibers^261^. The same year, systematic delivery of AAV encoding ABE achieved 96% restoration of dystrophin expression in humanized ^∆mE5051, KIhE50/Y^ mice. However, the target specificity and the size of the ABE remain a challenge^262^. *In vivo* CRISPR therapies could potentially provide solutions for acquired skeletomuscular diseases as well. Sarcopenia, or muscle wasting, is characterized by progressive loss of muscle mass due to injury, chronic disease, or ageing. It affects 10-16% of the global elderly population^263^. Inhibition or disruption of the myostatin-encoding *MSTN* gene may help prevent further muscle loss. *In vivo* MSTN disruption was achieved using a AAV8 system delivering CRISPR/SaCas9 to mouse muscle cells. Although only 5% of editing was achieved, more than 25% of muscle function was preserved. Further advances in targeting and editing efficiency will be required before first-in-human application.

### Skin

The skin is the largest organ in the human body, covering approximately 1.5-2.0 m² in adults and accounting for ~15% of body weight. It serves as a dynamic barrier that protects against mechanical, chemical, and microbial insults while maintaining hydration, thermoregulation, and immune homeostasis^264^. Human skin is an attractive yet challenging target for genome editing. Its direct accessibility enables diverse delivery strategies, including topical, intradermal, microneedle-based, and surgical or *ex vivo* approaches. Importantly, multiple clinically relevant cell types, such as keratinocytes, epidermal stem cells, fibroblasts, melanocytes, and resident immune cells are present in the skin. Many severe monogenic skin disorders originate in keratinocytes or fibroblasts and may be amenable to durable correction through genome editing of skin-resident stem cells^265^. Genome editing is also being explored to modulate inflammation, enhance wound healing, and treat pre-malignant or malignant lesions^266^,^267^. However, efficient *in vivo* delivery remains the primary limitation. The stratum corneum and epidermal architecture severely restrict penetration of large macromolecules such as Cas nucleases, RNPs, and plasmid DNA^268^. Additional challenges include stem cell targeting, immune responses to delivery systems, and layer-specific heterogeneity, all of which shape current dermatologic genome-editing strategies.

*In vivo* CRISPR delivery into the skin employs diverse methods including viral vectors, LNPs and ligand-conjugated proteins, or hybrid strategies such as delivery of RNPs with electroporation. AAV is widely used for *in vivo* gene delivery because of its high transduction efficiency and good safety profile in many organs, but it has limited cargo capacity which poses challenges for delivery of full-size SpCas9, as well as variable tropism for keratinocytes versus fibroblasts. Smaller Cas orthologs (such as SaCas9) or split-Cas approaches are therefore used to accommodate AAV packaging constraints. AAV-based strategies are well suited for local intradermal injection targeting dermal fibroblasts, although efficient delivery to basal epidermal stem cells remains difficult^269^. Lentivirus and other integrating vectors are often used for *ex vivo* modification because they enable stable transgene expression; however, they are generally avoided for *in vivo* skin editing due to the risk of insertional mutagenesis. Herpes simplex virus (HSV) is attractive because of its large cargo capacity and natural skin tropism and has therefore been explored preclinically, although safety and control issues persist^268^,^270^. LNPs are currently at the forefront of topical and intradermal *in vivo* editing strategies. Adapted from mRNA therapeutics, LNPs can deliver Cas effectors mRNA, sgRNA, or even RNPs through transient hit-and-run approaches^268^.

Recent reviews propose topical “gene-creams” (LNP formulations) as feasible for localized genodermatoses when combined with barrier disruption or microneedle-assisted delivery^271^. Encouraging preclinical studies have demonstrated LNP-mediated delivery to the viable epidermis and dermis under assisted penetration conditions. Polymeric nanoparticles, including PLGA and PEI derivatives, offer tunable release properties and have been explored for localized delivery in wound models^272^. Inorganic nanoparticles, particularly gold nanoparticles, represent highly stable nanocarriers and have been used for photo-triggered release and enhanced penetration in several preclinical studies^273^. Among physical delivery strategies, microneedles (solid, coated, dissolvable, or hollow) are considered especially promising for localized skin editing. These systems create controlled microchannels that enable delivery of RNPs, LNPs, or mRNA into the epidermis and dermis with minimal pain and good patient acceptability. Several recent reviews and preclinical reports have highlighted the potential of microneedle-assisted cargo delivery for skin and wounds applications^274^. Similarly, laser microporation using fractional ablative lasers generates transient channels that facilitate topical nanoparticle penetration and enhanced local uptake in preclinical models^269^. Electroporation and sonoporation can further improve plasmid or RNP uptake, although their relatively invasive and painful nature has largely restricted their use to experimental *in vitro* models and large animal models^269^. Much recent progress has been made through hybrid delivery strategies that combine chemical carriers with physical disruption methods, such as LNPs with microneedles or laser microporation. These strategies enable high local dosing with transient exposure, thereby improving the safety profile while supporting efficient editing. Consequently, hybrid delivery systems are increasingly viewed as the main path toward clinically translatable topical gene-editing platforms, including gene creams applied after microneedle array or fractional laser treatment^271^. Depending on the delivery approach, editing efficiencies of *in vivo* CRISPR genome skin editing have been reported to range from 2% to 85%. Proof-of-concept studies include excision of exon 80 in COL7A1 to restore collagen VII and improve dermal-epidermal adhesion^275^, correction of congenital ichthyosis mutations^268^, and gene knockout for tumor suppression in skin and oral epithelium.

### Urinary System

To date, only a few reports have described direct *in vivo* delivery of CRISPR-based editing systems to the urinary tract. This is not due to the lack of need of *in vivo* editing in this system, but rather reflects the considerable challenges associated with targeting renal and uroepithelial tissues, including anatomical complexity, cellular heterogeneity and physiological barriers such as glomerular filtration and tubular clearance. Two important programs are driving the *in vivo* genome editing for urinary tract related disorder. Jiang *et al*. demonstrated an efficient LNP-delivered CRISPR/Cas9 *in vivo* editing strategy for the treatment of primary hyperoxaluria type 1 (PH1) by targeting the *HAO1* gene in hepatocytes. Using LNPs to deliver Cas9 mRNA and guide RNA, the authors achieved robust disruption of glycolate oxidase (GO), a key upstream enzyme in oxalate biosynthesis. In PH1 mouse models, this resulted in marked and durable reductions in urinary oxalate levels, prevention of calcium oxalate crystal deposition, and improvement of renal pathology, while maintaining a favorable safety profile with minimal off-target editing. The study provided strong preclinical proof-of-concept that permanent hepatic *HAO1* knockout can serve as an effective therapeutic strategy and laid the translational foundation for the clinical development of YOLT-203^276^. ABO-101 is on the other hand an LNP-delivered *in vivo* CRISPR gene-editing therapy currently in Phase 1/2 clinical development for primary hyperoxaluria type 1 (PH1). The therapy employs a novel Cas12i2 nuclease to permanently disrupt hepatic *HAO1*, thereby reducing oxalate production through metabolic pathway reprogramming.

Most genome editing efforts have therefore been done on models such as iPSCs, organoids and relevant cell lines. Several studies have demonstrated successful correction of pathogenic variants in patient derived iPSCs, providing important proof-of-concept for therapeutic genome editing. For example, Lim et.al. corrected mutations associated with Gitelman syndrome in patient-derived iPSCs using ssODN mediated HDR with CRISPR/Cas9. The correction resulted in restoration of levels of key biomarkers like NCCT in derived organoids^277^. Similarly, Wang and colleagues used a dual AAV strategy to deliver a split-ABE system in order to edit organoids derived from hiPSCs of a Autosomal Dominant Polycystic Kidney Disease (ADPKD) patient. They achieved a 9.48% reversion of the mutated allele back to wild-type^278^. Since ADPKD is caused by mutations in *PKD1* and *PKD2*, with over 500 identified mutations, miR-17~92 has also been looked at as a potential candidate in treating ADPKD^279^.

Importantly, advances in renal-targeted delivery systems suggest that *in vivo* genome editing of the urinary tract may be feasible. Antibody-functionalized liposomal nanoparticles targeting vascular cell adhesion molecule 1 (VCAM-1) or E-selectin have been shown to selectively accumulate in inflamed renal endothelium and glomerular cells in mouse models, successfully delivering siRNA and reducing kidney inflammatio^280^. Although these studies do not involve CRISPR-based editors, they demonstrate that cell type specific delivery to renal tissues can be achieved *in vivo*. Together, these findings highlight a clear gap between robust *ex vivo* genome editing platforms and renal-targeted delivery technologies. This positions the urinary tract as a promising yet largely unexplored target for future *in vivo* CRISPR therapeutic development.

### Respiratory System

The respiratory system has developed a multi-layered defense mechanism to prevent foreign particulates and pathogens from reaching the alveolar space^281^. Alveolar macrophages (AMs) are the predominant resident immune cell population in the alveolar space and form a potent barrier to inhaled vectors^282^. To overcome anatomical, mucus, epithelial, and immune barriers, both viral and non-viral vectors have been engineered for lung tropism, enabling direct *in vivo* CRISPR gene editing. AAV vectors are the most widely used *in vivo* delivery platform. Capsid engineering of AAV6 produced AAV6.2, which enhances capsid stability and airway tropism^283^,^284^. AAVs were used to deliver split-Cas9 and a donor template to correct surfactant protein B (SP-B) deficiency in mice. This approach achieved precise HDR-mediated insertion of Sftpb into the *Sftpc* locus in approximately 25% of Angiotensin TII cells, restoring surfactant production and significantly prolonging survival^285^. Adenoviral vectors have been used to introduce chromosomal rearrangements characteristic of human lung cancer: intratracheal administration of CRISPR-Cas9 adenoviruses induced specific inversions generating the Eml4-Alk fusion oncogene and faithfully recapitulated non-small cell lung cancer pathology in wild-type mice^286^. In preclinical Cystic fibrosis models, F/HN-LVs delivering CRISPR-Cas9 components have achieved efficient gene disruption in airway epithelium without the toxicity associated with chemical tight junction disruption, and are being advanced towards first-in-human evaluation.

LNPs are the leading non-viral platform for nucleic acid delivery but naturally exhibit strong hepatic tropism. Lung-directed CRISPR delivery therefore requires rational engineering to retarget biodistribution and withstand the physical stresses of pulmonary administration^38^,^287^. SORT LNPs achieve this by incorporating supplemental cationic lipids such as DOTAP at defined molar fractions (typically 40-50%), which remodel the protein corona in circulation. Enrichment of vitronectin on the particle surface promotes αvβ3 integrin-mediated uptake into pulmonary endothelial and epithelial cells, redirecting LNPs from the liver to the lung^38^,^72^. SORT LNPs encapsulating Cas9 RNPs and single-stranded DNA templates have achieved approximately 35% editing efficiency in murine lungs, correcting a pathogenic *CFTR* mutation and restoring chloride channel function to therapeutic levels in Cystic fibrosis mouse models. RNP delivery shortens the duration of nuclease activity compared with mRNA or plasmid DNA, thereby reducing the risk of off-target effects while maintaining robust on-target correction. Building on this, lung-targeted SORT LNPs have been used to deliver ABE mRNA to lung stem cells, correcting CFTR mutations and sustaining functional rescue for up to 22 months in mice, demonstrating the potential of “one-and-done” base editing strategies^288^,^289^.

Nebulization is particularly attractive for targeting the airway epithelium but imposes substantial shear forces that destabilize conventional LNPs, leading to aggregation and cargo leakage. Charge-Assisted Stabilization (CAS) LNPs address this by incorporating peptide-lipid conjugates that confer strong surface charge and electrostatic repulsion, preserving particle integrity during aerosolization. In large animal models, inhaled CAS-LNPs mediated robust transfection of pulmonary dendritic and epithelial cells without significant airway inflammation^287^. Effective inhaled delivery also requires traversal of the airway mucus barrier. Dense PEG2000 surface modification (>5 mol%) renders LNPs muco-inert and prevents adhesion to mucin fibers, but high PEG density can impair cellular uptake and endosomal escape. Acid-degradable PEG-lipids resolve this trade-off by stabilizing LNPs during mucus transit and then cleaving in the acidic endosomal environment, exposing a fusogenic lipid core that promotes endosomal escape and cytosolic release of CRISPR cargo^290^. Additionally, polymeric vectors offer a non-viral alternative for intrapulmonary delivery. Poly(β-amino ester) (PBAE) polymers administered intratracheally have efficiently delivered Cas9 mRNA and sgRNAs to lung tumours, achieving approximately 69% indel formation at the Kras-G12S locus and resulting in significant tumour growth inhibition and apoptosis *in situ*^291^. With continued progress in the development of lung-targeted LNPs, this strategy may hold considerable promise for future direct *in vivo* genome editing applications in the respiratory system.

### Visual System

The visual system offers a uniquely favorable yet technically demanding setting for *in vivo* genome editing. Anatomically, the eye is divided into an anterior segment, comprising the cornea, iris, ciliary body, lens, and aqueous humor, and a posterior segment that includes the vitreous humor, retina, choroid, and optic nerve. Light traverses the anterior segment to reach the retina, where photoreceptors convert photons into electrical signals relayed to the brain. From a delivery perspective, the cornea forms a tight epithelial barrier that limits penetration into the anterior segment, whereas the posterior segment is protected by the blood-retinal barrier, composed of retinal capillary endothelial cells and the retinal pigment epithelium (RPE). This barrier preserves retinal homeostasis but severely restricts systemic access of macromolecules, including CRISPR-based editing systems. As a result, direct local administration is required, most commonly through subretinal injection, which deposits cargo between the RPE and photoreceptor outer segments, or intravitreal injection, which is less invasive but generally less efficient for photoreceptor targeting^16^,^17^,^292^. Inherited retinal diseases represent a major target for ocular genome editing, affecting approximately one in 2,000 individuals worldwide and encompassing both dominant and recessive disorders. Over the past decade, CRISPR-Cas technologies have been extensively explored to disrupt dominant toxic alleles, correct pathogenic variants, or modulate gene expression in retinal cells. Both viral and non-viral delivery strategies have been evaluated, with adeno-associated virus vectors emerging as the most widely used platform due to their high transduction efficiency, relative immune privilege of the eye, and favorable safety profile. The limited packaging capacity of AAV necessitates the use of compact Cas9 orthologs or split systems, but it remains sufficient to accommodate smaller nucleases such as SaCas9 together with guide RNAs in single-vector formats^293^. The first CRISPR-based therapy to reach the clinic for an inherited retinal disorder was EDIT-101, designed to excise the pathogenic IVS26 splice variant in the CEP290 gene responsible for Leber congenital amaurosis type 10. This strategy uses AAV5 to deliver SaCas9 and two guide RNAs via subretinal injection. In the Phase 1/2 BRILLIANCE trial, treatment was generally well tolerated and approximately 43 % of participants experienced meaningful improvements in vision-related quality-of-life measures, although overall visual rescue was modest and the program has since been paused. These results nevertheless provided proof of concept for *in vivo* genome editing in the human retina. In parallel, all-in-one AAV approaches have been developed to inactivate genes such as *Nrl* in mouse models of retinitis pigmentosa, leading to preservation of photoreceptors and cone function, and compact transcriptional activator systems based on dCasMINI have enabled single-AAV gene reactivation strategies that improve retinal function following intravitreal delivery^137^,^294^,^295^.

To further address AAV cargo constraints, several compact or alternative Cas platforms have been introduced. A Cas9 ortholog derived from an uncultivated Collinsella species, CoCas9, is sufficiently small for all-in-one AAV delivery and has been applied to target the P23H mutation in rhodopsin associated with autosomal dominant retinitis pigmentosa, achieving measurable editing *in vivo*. Dual-AAV strategies have also been used to deliver larger nucleases or more complex payloads, including approaches that either selectively disrupt dominant rhodopsin alleles or combine mutation-independent gene ablation with replacement of wild-type cDNA to overcome allelic heterogeneity. While effective in animal models, these strategies require high rates of biallelic editing, which remains challenging to achieve *in vivo*^296^,^297^. Base editing and prime editing have expanded the therapeutic scope for retinal diseases by enabling precise correction of point mutations without inducing DSBs. Dual-AAV delivery systems exploiting intein-mediated protein splicing have been used to deliver adenine base editors to photoreceptors and RPE cells in non-human primates and mouse models, achieving consistent on-target editing of *ABCA4* or *RHO* variants with limited bystander effects. Prime editing has similarly been adapted to the retina using split AAV systems or compact editors, enabling targeted modification of genes such as *DNMT1*, *PDE6B*, and *RPE65* and resulting in improved retinal structure and function in relevant disease models^298^,^299^. Despite their success, AAV-based strategies raise safety considerations related to persistent episomal DNA in post-mitotic retinal cells, prolonged nuclease expression, and the potential for AAV integration at CRISPR-induced DNA breaks. These concerns have motivated the exploration of transient delivery platforms. VLPs offer a DNA-free alternative by delivering Cas9 or base editor RNPs directly, and programmable VLPs have been shown to reduce choroidal neovascularization in mouse models of retinal vascular disease following subretinal administration^300^,^301^. Non-viral nanotechnology-based approaches have also gained prominence as a means to achieve transient editing and improved safety. LNPs, lipoplexes, nanocapsules, electroporation, and direct RNPs delivery have all been evaluated for ocular applications. Direct subretinal delivery of Cas9 or base editor ribonucleoproteins has achieved efficient editing of photoreceptors and RPE cells without viral vectors. Dynamically covalent LNPs delivering Cas9 mRNA and guide RNAs targeting *VEGFA* have produced marked reductions in choroidal neovascularization after intravitreal injection. However, while RPE cells are readily transfected by conventional LNPs, photoreceptors remain more difficult to target, and certain anionic formulations have induced retinal toxicity, underscoring the need for careful optimization^302^-^304^. Further refinements using biodegradable polymeric nanocapsules and inorganic nanoparticles have enabled targeted delivery to the posterior segment. Nanocapsules decorated with all-trans-retinoic acid have selectively targeted RPE cells and achieved localized gene editing, while silica nanoparticles functionalized with glutathione or pH-sensitive chemistries have supported efficient RNP delivery with preservation of retinal function. These platforms have also been adapted to deliver base editors for rare pediatric blinding disorders such as Leber congenital amaurosis type 16, achieving therapeutically meaningful editing in RPE cells with minimal off-target effects^305^,^306^. Taken together, the eye represents one of the most advanced arenas for *in vivo* genome editing, with both viral and non-viral platforms already demonstrating therapeutic benefit in preclinical and early clinical studies. Continued optimization of delivery vehicles, editing modalities, and cell-specific targeting is expected to further enhance efficacy while mitigating safety concerns. These advances position ocular gene editing as a leading model for the broader translation of CRISPR-based therapeutics *in vivo*.

## Importance of *In Vivo* Studies and Regulatory Requirements for CRISPR-based Gene Editing

*In vivo* studies remain indispensable for the preclinical evaluation of CRISPR-based gene editing therapies, as they provide integrated information on safety, biodistribution, toxicity, immunogenicity, persistence, and potential off-target effects within a complex biological context that cannot be fully recapitulated *in vitro*. Such studies are essential for assessing the biological behavior of gene editing components and delivery systems at the organismal level, identifying dose-response relationships, and evaluating both short-term and long-term risks that are critical for regulatory decision-making and clinical translation.

The conduct of animal studies is governed by strict ethical and legal frameworks designed to ensure scientific validity and animal welfare. In the European Union, Directive 2010/63/EU on the protection of animals used for scientific purposes provides the legal foundation for animal experimentation. This directive requires that *in vivo* studies be scientifically justified, ethically reviewed, and authorized prior to initiation, and that procedures be designed to minimize pain, suffering, and distress. Central to this framework is the implementation of the principles of Replacement, Reduction, and Refinement (3R), which promote the use of alternative methods whenever possible, the minimization of animal numbers, and the refinement of experimental procedures to improve animal welfare^28^. Ethical oversight is further reinforced by recommendations from organizations such as the Federation of European Laboratory Animal Science Associations, which advocate for rigorous application of the principles of Replacement, Reduction, and Refinement and emphasize the use of alternative experimental models, including advanced *in vitro* systems, organoids, and computational approaches, whenever feasible.

From a regulatory perspective, the European Medicines Agency (EMA) has issued specific guidance for the non-clinical development of gene therapy medicinal products. The Guideline on the quality, non-clinical and clinical aspects of gene therapy medicinal products (EMA/CAT/80183/2014) emphasizes the need for comprehensive toxicological assessment, including dose-ranging, acute, repeat-dose, and long-term studies, to identify potential adverse effects, immune responses, and risks associated with genome editing components and their delivery vehicles. In parallel, the EMA guideline on non-clinical biodistribution studies (EMA/CHMP/ICH/318372/2021), often referred to as the S12 guideline, outlines expectations for characterizing the *in vivo* distribution, persistence, and clearance of gene therapy vectors using validated and sensitive analytical methods such as quantitative polymerase chain reaction, *in situ* hybridization, and next-generation sequencing. These studies are critical for understanding tissue exposure, potential off-target accumulation, and long-term safety, and for informing clinical dosing strategies. Effective from July 2025 EMA additionally provided Guideline on quality, non-clinical and clinical requirements for investigational advanced therapy medicinal products in clinical trials (EMA/CAT/22473/2025) for the development of Advanced therapy medicinal products (ATMPs). Similarly, the United States Food and Drug Administration (FDA) has issued guidance on non-clinical biodistribution considerations for gene therapy products (S12 Nonclinical Biodistribution Guidance). This document underscores the importance of well-designed biodistribution and toxicology studies conducted across relevant species and dose ranges, using robust analytical methodologies to assess vector persistence, tissue distribution, and potential unintended exposure. Such data are considered essential for evaluating long-term safety risks and supporting the transition from preclinical development to first-in-human studies.

## Clinical Progress and Therapeutic Translation of *In Vivo* Genome Editing

*In vivo* genome editing has moved from first-in-human feasibility to a broader (and increasingly diversified) clinical pipeline, even though no *in vivo* CRISPR genome-editing medicine had received regulatory approval as of late 2025. In contrast, the first approvals in the CRISPR space have been *ex vivo* products (for example, exagamglogene autotemcel, Casgevy) that edit hematopoietic stem cells outside the body and then reinfuse them^307^. A significant and utmost important milestone in precision genome editing as personalized medicine was recently reached with the compassionate-use treatment of a six-month-old infant with severe life-threatening CPS1 (carbamoyl phosphate synthetase 1) deficiency, a urea cycle disorder causing toxic hyperammonemia. A custom-designed, single-patient base-editing therapy, delivered via LNPs into the liver, improved nitrogen metabolism and reduced reliance on ammonia-scavenger medications, and led to normal growth progression, illustrating the feasibility of individualized *in vivo* genome editing for ultrarare diseases^85^. The therapy was developed within 6 months and administered in three intravenous doses beginning in February 2025 by investigators at CHOP and Penn Medicine.

Key ongoing clinical programs, delivery modalities, target organs, and development stages are summarized in **Table 4.** Clinically, the liver has remained the dominant launchpad for *in vivo* editing, because systemically dosed LNPs naturally accumulate in hepatocytes and enable transient expression of editors (e.g. Cas9, base editors, and related systems). This “pharmacology-like” delivery profile supports scalable dosing, avoids vector integration, and limits nuclease exposure time relative to long-lived expression systems^194^. The clearest clinical precedent is transthyretin (*TTR*) amyloidosis, where Intellia's Therapeutics NTLA-2001 (nexiguran ziclumeran; nex-z) established first-in-human systemic CRISPR editing with reports of large, durable reductions in circulating TTR after a single dose in early clinical follow-up. Ongoing NTLA-2001 studies and extensions can be tracked on ClinicalTrials.gov (including NCT04601051 and related protocols). NTLA-2001, targeting TTR, achieved up to 87% serum TTR reduction in patients with ATTR with only mild adverse events, reported in 2021 in a published study of 6 patients^186^. A follow up study of 36-treated patients, published in September 2025, revealed rapid, profound, and durable suppression of circulating transthyretin, with mean serum TTR reductions of approximately 90% by day 28, sustained through 24 months (-92%). Importantly, these robust pharmacodynamic effects were accompanied by encouraging clinical stabilization, with the majority of patients showing stable or improved polyneuropathy stage, disability scores, and biomarker profiles, including reductions in serum neurofilament light chain and improvement in modified Neuropathy Impairment Score+7 (mNIS+7). The treatment was generally well tolerated, with the most common adverse events, being transient infusion-related reactions (21 patients), mild decreases in thyroxine levels without clinical hypothyroidism (8 patients), and headache (4 patients). Although serious adverse events occurred in 11 patients, including one death attributed to underlying cardiac amyloidosis, the overall findings provided the first compelling evidence that one-time *in vivo* CRISPR-mediated gene disruption can achieve durable disease-modifying effects in a systemic protein-misfolding disorder, supporting further clinical development of this approach^308^. Late in October 2025, the FDA put a clinical hold on the clinical trial MGNITUDE as one individual enrolled on that trial developed severe grade 4 liver transaminases and increased total bilirubin. As of March 2 2026, Intellia Therapeutics announced that the clinical hold had been lifted following alignment of the trial protocol with FDA requests for enhanced liver monitoring, short-term steroid guidance for elevated liver transaminases after dosing, and exclusion of patients with certain liver abnormalities. A second major LNP-led axis is cardiometabolic prevention via hepatic lipid targets, where programs aim for durable lowering of atherogenic lipoproteins through one-time editing. This includes approaches targeting *ANGPTL3* (including the phase 1 experience reported for CTX310) and *PCSK9* (including base-editing strategies in clinical development). These trials collectively illustrate both the opportunity and the heightened safety bar for preventive indications, where acceptable risk is substantially lower than in severe, otherwise untreatable disease^74^,^161^. Base editing has also entered the clinic with VERVE-101, the first in-human base-editing therapeutic targeting *PCSK9* to lower LDL-C. Early data showed robust LDL-C reduction but raised safety concerns, including hepatic and cardiac toxicity and one fatal event^309^. Reduced LDLR-dependent LNP uptake in heterozygous familial hypercholesterolemia (HeFH) patients may have contributed to these outcomes, emphasizing the need for tailored delivery strategies in populations with impaired receptor function. In the Phase 1b Heart-1 trial (NCT05398029), enrollment of VERVE-101, was paused (announced by Verve Therapeutics) after the sixth participant developed asymptomatic Grade 3 transient ALT elevation and thrombocytopenia within four days of dosing. Both abnormalities resolved completely within days without clinical symptoms. Following DSMB (Data Safety and Monitoring board) review, Verve Therapeutics shifted development toward VERVE-102, a next-generation candidate using an optimized GalNAc-LNP delivery system, underscoring the importance of delivery platform design for the safety of *in vivo* gene-editing therapies. For hereditary angioedema (HAE), NTLA-2002 advanced the concept of *in vivo* editing toward diseases where a durable reduction in attack frequency could replace chronic prophylaxis. Trial identifiers and current recruitment status are maintained at ClinicalTrials.gov (including NCT05120830 and associated studies)^310^. NTLA-2002, targeting *KLKB1*, achieved durable kallikrein suppression and substantially reduced attack frequency in hereditary angioedema^311^. In parallel, AAV-enabled *in vivo* editing has concentrated in anatomically accessible or immune-privileged compartments, most notably the eye. EDIT-101 (AAV5 delivering SaCas9 and guides) was the first *in vivo* CRISPR program dosed in humans for CEP290-associated LCA10, with clinical trial (NCT03872479) details anchored in ClinicalTrials.gov and outcomes reported in the peer-reviewed literature^138^. In the phase 1/2 BRILLIANCE trial, subretinal delivery demonstrated a generally favorable safety profile and evidence of improved visual function in a subset of patients, providing an important clinical proof-of-concept for ocular *in vivo* genome editing. Despite these encouraging findings, Editas Medicine discontinued internal investment in the program in 2023 as part of a strategic portfolio reprioritization and has since sought external partners for further development. Systemic AAV delivery for infectious disease has also entered the clinic, exemplified by EBT-101 (AAV-based excision strategy for HIV). Trial registration and protocol details are available via ClinicalTrials.gov (NCT05144386), while interim translational discussions and limitations (including viral rebound dynamics) have been reported in the peer-reviewed and conference literature^312^. A distinct modality class is emerging for CRISPR-armed bacteriophages (for example, Cas3-based “genome shredding”) aimed at pathogen-selective antimicrobials. Early-phase programs such as LBP-EC01 and related SNIPR Biome pipelines demonstrate how genome editing is expanding beyond human somatic editing into *in situ* microbiological control, with trial identifiers and updates tracked in registries and infectious-disease outlets^313^. Gene-editing approaches are also being explored for chronic viral infections. In the ELIMINATE-B trial, the ARCUS nuclease therapy (PBGENE-HBV) achieved up to 69% reduction in HBsAg in patients with chronic HBV, demonstrating the potential of directly targeting viral cccDNA to achieve a functional cure^314^. More recently, TUNE-401 was developed as a dCas9-based epigenetic silencer that couples a DNA methyltransferase with an additional repressive effector domain to exploit a key vulnerability in HBV's epigenetic control. Delivered via LNPs it targets a highly conserved regulatory sequence shared by both integrated HBV DNA and cccDNA, and by inducing DNA methylation and heterochromatin formation at this locus, TUNE-401 establishes a durable repressed state capable of inactivating all viral forms. Across these modalities, the most consequential “integrative” constraint is increasingly CMC and comparability: controlling particle composition, potency, encapsulation efficiency, impurity profiles, and batch-to-batch reproducibility at scale. This is especially acute for LNPs (where microfluidic mixing, lipid raw-material control, and analytical release assays are central) and for AAV (where full-to-empty ratios, capsid heterogeneity, and residual host-cell impurities can materially affect both efficacy and safety)^36^,^315^. Regulatory expectations have likewise matured into more explicit, genome-editing-specific frameworks. In the US, FDA's final guidance for human gene therapy products incorporating genome editing emphasizes product characterization, biodistribution and shedding, durability, immunogenicity, and risk assessment for unintended editing outcomes^316^. In the EU, EMA guidance for investigational ATMPs and gene therapy products outlines quality, non-clinical, and clinical expectations that directly map onto *in vivo* editors (including potency strategy, comparability, and risk-based non-clinical packages)^317^. Taken together, the current clinical landscape (**Table 4**) is best described as modality-partitioned translation: LNPs dominate liver-directed programs (TTR, HAE, cardiometabolic targets), AAV remains most mature in ocular and select niche indications, and phage-based CRISPR is opening an antimicrobial frontier. The unifying trajectory is clear: as delivery becomes more tissue-selective and manufacturing becomes more standardized, clinical programs are increasingly positioned to move from “first feasibility” toward repeatable benefit-risk profiles appropriate for common diseases, not only rare, high-severity indications.

## Conclusions and Perspectives

The past decade has transformed CRISPR-based genome and epigenome editing from a molecular biology tool into a credible therapeutic modality. Advances in nuclease engineering, base and prime editing, and programmable transcriptional control have dramatically expanded the scope of possible genetic interventions. However, as this review emphasizes, the dominant bottleneck for clinical translation is no longer editing chemistry itself, but rather the safe, efficient, and tissue-selective delivery of editing cargo *in vivo*. Across organ systems, delivery constraints emerge as highly context dependent. Immune cells demand transient, non-integrating platforms capable of targeting mobile and rapidly renewing populations, favoring receptor-directed LNPs and EVs. The nervous system and retina impose formidable anatomical barriers, where viral vectors remain most mature but are increasingly complemented by non-viral nanotechnologies and transient RNP delivery. Cardiovascular applications have progressed most rapidly through liver-directed editing, leveraging hepatotropic LNP pharmacology to achieve durable cardiometabolic risk reduction, while direct myocardial and vascular targeting remains an open challenge. In the GI tract, the complexity of mucosal barriers and microbiome interactions has driven parallel innovation in host-directed nanoparticles and microbe-specific phage or conjugative systems, underscoring that genome editing is expanding beyond human somatic cells into ecological and microbiological domains. Clinically, *in vivo* genome editing has moved decisively from first-in-human feasibility toward modality-partitioned translation. LNPs dominate systemic liver-directed programs, AAVs retain a strong position in ocular and anatomically accessible tissues, and CRISPR-armed bacteriophages represent an emerging antimicrobial frontier. At the same time, regulatory expectations have matured, placing increasing emphasis on delivery characterization, biodistribution, immunogenicity, durability, and manufacturing comparability. These considerations are now inseparable from editor design and therapeutic intent, particularly as indications expand from rare, severe diseases toward more common preventive or chronic conditions with lower acceptable risk thresholds. Looking forward, several unifying priorities will shape the next phase of *in vivo* genome editing. First, delivery platforms must achieve greater cell-type specificity within complex tissues while minimizing systemic exposure and immune activation. Second, transient and programmable editor expression will be essential to balance efficacy with long-term safety, especially in proliferative or immune-reactive environments. Third, scalable and reproducible manufacturing, supported by robust analytical frameworks, will increasingly determine which platforms can advance beyond proof-of-concept into widespread clinical use. Finally, as genome editing extends to the microbiome and other non-human targets, careful consideration of ecological and evolutionary consequences will be required alongside traditional toxicological assessment. In sum, *in vivo* genome editing is transitioning from a technology-driven field to a delivery-governed discipline. Continued convergence of molecular engineering, materials science, immunology, and regulatory science will be essential to translate the remarkable precision of CRISPR-based tools into durable, safe, and broadly applicable therapies. The coming years are likely to see not only new editors, but more importantly, new delivery paradigms that ultimately define the clinical impact of genome editing in human disease.

Despite rapid advances in CRISPR-based editing modalities, delivery remains the central bottleneck for successful *in vivo* genome editing. The choice of delivery system dictates not only editing efficiency, but also tissue specificity, duration of activity, safety profile, and the feasibility of repeat administration. Future progress in the field will therefore depend less on expanding the diversity of editing tools and more on developing delivery platforms capable of precise, controllable, and scalable genome modification in clinically relevant settings. Ultimately, the clinical success of CRISPR technologies will be defined by the ability to engineer delivery systems that translate molecular precision into predictable and safe therapeutic outcomes.

## Figures and Tables

**Table 1:** Comparison of CRISPR-based genome and epigenome editing platforms

Category	Conventional CRISPR/Cas9	Base Editing (BE)	Prime Editing (PE)	Epigenome Editing
Core components	Cas nuclease (e.g. SpCas9), single guide RNA (sgRNA); optional donor DNA for HDR	Cas9 nickase or dCas9 fused to cytidine or adenosine deaminase; sgRNA	Cas9 nickase fused to reverse transcriptase; prime editing guide RNA (pegRNA)	Catalytically inactive Cas9 (dCas9) fused to epigenetic effector domains; sgRNA
DNA cleavage type	Double-strand breaks (DSBs)	Single-strand nick on target or non-target strand	Single-strand nick on non-target strand	No DNA cleavage
Primary repair pathway(s)	NHEJ, MMEJ (dominant); HDR (minor, cell-cycle restricted)	Base excision repair and mismatch repair	Flap resolution and DNA repair synthesis	No DNA repair required
Editing outcomes	Gene disruption; indels; large insertions or deletions with donor templates	Single-nucleotide transitions (C↔T, A↔G) within editing window	All 12 base substitutions; small insertions and deletions	Reversible gene activation or repression without sequence alteration
Precision	Moderate; indel heterogeneity common	High within editing window; bystander edits possible	High; edit-defined outcomes	High at targeted loci; chromatin context dependent
Off-target risks	DSB-induced indels; chromosomal rearrangements	DNA and RNA off-target deamination	Lower indel burden; efficiency-dependent off-target risk	Off-target chromatin modulation
Editing efficiency	High for knockouts; low for HDR	High for compatible transitions	Moderate to low; context dependent	Variable; locus and effector dependent
Payload size constraints	Moderate; Cas9 alone fits AAV	Large; often exceeds AAV capacity	Large; exceeds AAV capacity	Moderate; depends on effector
Strengths (+)	Robust cleavage; versatile gene knockout; compatible with many delivery platforms	Precise SNV correction; no DSBs; high efficiency	Broad sequence versatility; no DSBs or donor DNA	Reversible, programmable regulation; no permanent DNA changes
Limitations (-)	Genotoxicity risk; unpredictable repair outcomes	Limited to transitions; bystander edits; delivery challenges	Lower efficiency; limited insertion size; complex pegRNA design	Transient effects; delivery and specificity challenges
Clinical translation status	Multiple *in vivo* trials (LNP, AAV)	*In vivo* trials emerging (liver, eye)	Early preclinical to translational	Preclinical to early translational

**Table 2:** ** Platform-resolved comparison of viral and non-viral CRISPR delivery systems for in vivo applications.** The table highlights fundamental trade-offs between viral platforms, which prioritize efficiency and tissue specificity, and non-viral systems, which favor transient expression, safety, and dosing flexibility. Delivery systems are compared across key translational parameters, including cargo capacity, expression profile, tissue targeting, redosing potential, and clinical readiness.

Delivery platform	Cargo capacity	Preferred CRISPR format	Expression profile	Tissue targeting	Redosing	Why redosing is limited or feasible	Immunogenicity	Key advantages	Key limitations	Clinical/translational status
AAV	~4.7 kb (limited; dual vectors needed)	DNA	Long-term	Strong, serotype-dependent (CNS, retina, liver)	Limited	Neutralizing antibodies against capsid prevent re-transduction	Moderate	High efficiency, defined tropism, clinical maturity	Payload constraint, AAV integration at DSBs	Leading platform; multiple clinical trials
Adenovirus (AdV)	High (up to ~36 kb)	DNA	Transient-moderate	Broad, liver-biased (modifiable)	Limited	Strong innate and adaptive immune responses clear vector and block re-administration	High (reduced in HCAdV)	Large cargo capacity, strong transduction	Immunogenicity, toxicity, pre-existing immunity	Limited CRISPR clinical use
Lentivirus (LV / IDLV)	~8 kb	DNA (or transient mRNA in IDLV)	Long-term (LV), transient (IDLV)	Broad (incl. non-dividing cells)	Limited	Anti-vector immunity and stable integration reduce need/feasibility of repeat dosing	Moderate	Efficient delivery, well-established platform	Integration risk (LV), prolonged expression	Mostly *ex vivo*; limited *in vivo*
Virus-like particles (VLPs)	Moderate	RNP, base/prime editors	Transient	Broad (envelope-dependent)	Potential	Lack of viral genome reduces adaptive immune memory; repeat dosing may be feasible depending on envelope	Low-moderate	DNA-free delivery, transient exposure	Targeting specificity, manufacturing complexity	Emerging (preclinical)
Lipid nanoparticles (LNPs)	High	mRNA, RNP	Transient (“hit-and-run”)	Liver-dominant; tunable (e.g., SORT)	Yes	No anti-vector neutralizing immunity; clearance allows repeated administration	Low-moderate	Clinically advanced, scalable, safe	Limited extrahepatic targeting, endosomal escape	Most advanced non-viral; clinical trials
Polymeric nanoparticles (PEI, PBAE, etc.)	High	DNA, mRNA, RNP	Transient	Tunable but variable	Yes	Minimal adaptive immunity enables repeat dosing; limited mainly by toxicity	Variable	Design flexibility, large cargo	Toxicity, lower efficiency, variability	Preclinical
Inorganic nanoparticles (e.g. AuNPs, silica, BPs, IONs)	High	RNP, DNA	Transient	Tunable; often RES accumulation	Yes	No neutralizing immunity; repeat dosing possible but limited by accumulation/toxicity	Variable	Precise engineering, multifunctionality	Accumulation, long-term safety concerns	Preclinical
Extracellular vesicles (EVs)	Moderate	RNP, mRNA, DNA	Transient	Potentially broad; can cross BBB	Yes	Endogenous origin reduces immune recognition, enabling repeated administration	Low	High biocompatibility, natural delivery	Heterogeneity, scalability issues	Emerging
Cell-penetrating peptides (CPPs)	Low-moderate	RNP	Transient	Limited intrinsic specificity	Yes	Minimal immunogenicity allows repeat dosing; efficiency remains limiting	Low	Simple, low toxicity	Low efficiency, endosomal escape	Early-stage

**Table 3:** Comparative Design Matrix of *In vivo* Delivery Modalities

Delivery Platform	Cargo Format	Expression Duration	Redosing Feasible	Selectivity Mechanisms	Key Safety Risks	Key CMC Challenges
AAV	DNA (full-length or split Cas systems)	Persistent	Limited (neutralizing antibodies)	Intrinsic tropism: Yes (serotypes)Ligand targeting: Emerging	Immunogenicity: Moderate-highIntegration risk: Low (DSB-associated)Possible off target effects and chromosomal aberrations due to persistent exposure	Empty/full capsid ratioEncapsulation efficiencyBatch-to-batch consistencyScalability - moderate limitation
Adenovirus	DNA	Transient	Limited (strong immunity)	Intrinsic tropism: BroadLigand targeting: Possible	Immunogenicity: HighIntegration risk: None	Impurity controlBatch consistencyImmunogenic contaminants (critical risk)Scalability - favorable
Lentivirus	DNA (Cas9 + sgRNA expression cassettes)	Persistent (integrating)	Limited	Intrinsic tropism: BroadLigand targeting: Possible (pseudotyping)	Immunogenicity: ModerateIntegration risk: High (insertional mutagenesis)Possible off target effects and chromosomal aberrations due to persistent exposure	Vector integration competencyBatch consistencyReplication-competent virus risk (critical risk)Scalability - moderate limitation
LNP	mRNA / RNP	Transient	Yes	Intrinsic tropism: Liver-biased, modifiable (GalNAc, SORT)Ligand targeting: Possible (conjugation, ASSET)	Immunogenicity: ModerateIntegration risk: None	Encapsulation efficiencyParticle size distributionBatch-to-batch consistencyScalability - favorable
VLP	mRNA / RNP	Transient	Potentially	Intrinsic tropism: Capsid-dependentLigand targeting: Possible	Immunogenicity: ModerateIntegration risk: None	Cargo loading efficiencyParticle heterogeneityBatch consistencyScalability - limiting factor
Extracellular vesicles (EVs)	mRNA / RNP	Transient	Yes (low immunogenicity)	Intrinsic tropism: LimitedLigand targeting: Emerging	Immunogenicity: LowIntegration risk: None	Cargo loading efficiencyParticle heterogeneityBatch consistencyScalability - limiting factor
Polymeric nanoparticles	DNA / mRNA / RNP	Transient	Yes	Intrinsic tropism: LimitedLigand targeting: Yes	Immunogenicity: VariableIntegration risk: None (unless DNA)	Encapsulation efficiencyParticle size distributionBatch consistencyScalability - moderate limitation
Physical methods ( e.g. Electroporation, etc.)	DNA / mRNA / RNP	Transient	Repeatable (localized)	Intrinsic tropism: NoLigand targeting: No	Immunogenicity: LowIntegration risk: Possible (DNA-based delivery)	Device-dependent variabilityReproducibilityTissue damage risk (critical risk)

**Table 4:** Clinical landscape of *in vivo* genome-editing therapeutics (*status through late 2025*)

Program	Developer	Editing modality	Delivery platform	Target tissue	Indication	Clinical status
NTLA-2001	Intellia Therapeutics	Cas9 nuclease knockout *TTR*	LNP (systemic) Cas9 mRNA + sgRNA	Liver (hepatocytes)	Transthyretin amyloidosis	NCT04601051 Phase 1
						NCT06128629 Phase 2
						NCT06672237 Phase 3
NTLA-2002	Intellia Therapeutics	Cas9 nuclease knockout *KLKB1*	LNP (systemic) Cas9 mRNA + sgRNA	Liver	Hereditary angioedema	NCT05120830 Phase 1/2
						NCT06634420 Phase 3
CTX310	CRISPR Therapeutics	Cas9 nuclease knockout *ANGPTL3*	LNP (systemic) Cas9 mRNA + sgRNA	Liver	*ANGPTL3*-driven dyslipidemia	ACTRN12623000809639 Phase 1
CTX320	CRISPR Therapeutics	Cas9 nuclease knockout *LPA*	LNP (systemic) Cas9 mRNA + sgRNA	Liver	*Lpa*-driven dyslipidemia	ACTRN12623001095651 Phase 1
VERVE-101	Verve Therapeutics	*PCSK9* Adenine base editor	LNP (systemic) ABE mRNA	Liver	Hypercholesterolemia	NCT05398029 Phase 1b
VERVE-101	Verve Therapeutics	*PCSK9* Adenine base editor	LNP (systemic) ABE mRNA optimized	Liver	Hypercholesterolemia, ASCVD risk	NCT06164730 Phase 1
EDIT-101	Editas Medicine	SaCas9 nuclease Knockout *CEP290*	AAV5 (subretinal)	Retina	LCA10 (CEP290)	NCT03872479 Phase 1/2
EBT-101	Excision BioTherapeutics	Cas9 excision (dual guide) proviral DNA	AAV9 (intravenous)	Lymphoid tissues	HIV-1 infection	NCT05144386 Phase 1/2
LBP-EC01 / SNIPR programs	Locus Biosciences	CRISPR-Cas3	Bacteriophage	Bacterial pathogens	Antimicrobial infections of urinary tract (drug resistant *E.coli*)	NCT05488340 Phase 1/2
YOLT-203	YolTech Therapeutics	Cas12 nuclease knockout *HAO1*	LNP (systemic) Cas12 mRNA + crRNA	Kidneys	Type 1 primary hyperoxaluria (PH1)	NCT06511349 Phase 1
ABO-101	Arbor Biotechnologies	Cas9 nuclease knockout *HAO1*	LNP (systemic) Cas9 mRNA + sgRNA	Kidneys	Type 1 primary hyperoxaluria (PH1)	NCT06839235 Phase 1/2
HG-302	HuidaGene Therapeutics	Cas12MAX Exon reframing of *DMD*	AAV	Muscles	Duchenne muscular dystrophy	NCT06594094 Early Phase I

## References

[1] Jinek M, Chylinski K, Fonfara I, Hauer M, Doudna JA, Charpentier E (2012). A programmable dual-RNA-guided DNA endonuclease in adaptive bacterial immunity. Science.

[2] Barrangou R, Fremaux C, Deveau H, Richards M, Boyaval P, Moineau S (2007). CRISPR provides acquired resistance against viruses in prokaryotes. Science (New York, NY).

[3] Hsu PD, Scott DA, Weinstein JA, Ran FA, Konermann S, Agarwala V (2013). DNA targeting specificity of RNA-guided Cas9 nucleases. Nature biotechnology.

[4] Jinek M, Jiang F, Taylor DW, Sternberg SH, Kaya E, Ma E (2014). Structures of Cas9 endonucleases reveal RNA-mediated conformational activation. Science.

[5] Kleinstiver BP, Prew MS, Tsai SQ, Topkar VV, Nguyen NT, Zheng Z (2015). Engineered CRISPR-Cas9 nucleases with altered PAM specificities. Nature.

[6] Zhang Y, Rajan R, Seifert HS, Mondragón A, Sontheimer EJ (2015). DNase H Activity of Neisseria meningitidis Cas9. Molecular cell.

[7] Kim E, Koo T, Park SW, Kim D, Kim K, Cho HY (2017). *In vivo* genome editing with a small Cas9 orthologue derived from Campylobacter jejuni. Nature communications.

[8] Hu JH, Miller SM, Geurts MH, Tang W, Chen L, Sun N (2018). Evolved Cas9 variants with broad PAM compatibility and high DNA specificity. Nature.

[9] Ren Q, Sretenovic S, Liu S, Tang X, Huang L, He Y (2021). PAM-less plant genome editing using a CRISPR-SpRY toolbox. Nature plants.

[10] Komor AC, Kim YB, Packer MS, Zuris JA, Liu DR (2016). Programmable editing of a target base in genomic DNA without double-stranded DNA cleavage. Nature.

[11] Grünewald J, Zhou R, Iyer S, Lareau CA, Garcia SP, Aryee MJ (2019). CRISPR DNA base editors with reduced RNA off-target and self-editing activities. Nature biotechnology.

[12] Gehrke JM, Cervantes O, Clement MK, Wu Y, Zeng J, Bauer DE (2018). An APOBEC3A-Cas9 base editor with minimized bystander and off-target activities. Nature biotechnology.

[13] Koblan LW, Doman JL, Wilson C, Levy JM, Tay T, Newby GA (2018). Improving cytidine and adenine base editors by expression optimization and ancestral reconstruction. Nature biotechnology.

[14] Hu J, Guo M, Gao Q, Jia H, He M, Wang Z (2026). QBEmax is a sequence-permuted and internally protected base editor. Nat Biotechnol.

[15] Yu W, Wang Y, Li S, Dai Y, Li Y, Zhang X (2025). Optimized dual-AAV base editor delivery system with enhanced editing efficiency and virion production titer. Synthetic and Systems Biotechnology.

[16] Anzalone AV, Randolph PB, Davis JR, Sousa AA, Koblan LW, Levy JM (2019). Search-and-replace genome editing without double-strand breaks or donor DNA. Nature.

[17] Chen PJ, Hussmann JA, Yan J, Knipping F, Ravisankar P, Chen PF (2021). Enhanced prime editing systems by manipulating cellular determinants of editing outcomes. Cell.

[18] Doman JL, Pandey S, Neugebauer ME, An M, Davis JR, Randolph PB (2023). Phage-assisted evolution and protein engineering yield compact, efficient prime editors. Cell.

[19] Yan J, Oyler-Castrillo P, Ravisankar P, Ward CC, Levesque S, Jing Y (2024). Improving prime editing with an endogenous small RNA-binding protein. Nature.

[20] Hilton IB, D'Ippolito AM, Vockley CM, Thakore PI, Crawford GE, Reddy TE (2015). Epigenome editing by a CRISPR-Cas9-based acetyltransferase activates genes from promoters and enhancers. Nature biotechnology.

[21] Liu XS, Wu H, Ji X, Stelzer Y, Wu X, Czauderna S (2016). Editing DNA Methylation in the Mammalian Genome. Cell.

[22] Morita S, Noguchi H, Horii T, Nakabayashi K, Kimura M, Okamura K (2016). Targeted DNA demethylation *in vivo* using dCas9-peptide repeat and scFv-TET1 catalytic domain fusions. Nature biotechnology.

[23] Sgro A, Blancafort P (2020). Epigenome engineering: new technologies for precision medicine. Nucleic acids research.

[24] Konermann S, Brigham MD, Trevino AE, Joung J, Abudayyeh OO, Barcena C (2015). Genome-scale transcriptional activation by an engineered CRISPR-Cas9 complex. Nature.

[25] Park H, Shin J, Kim Y, Saito T, Saido TC, Kim J (2022). CRISPR/dCas9-Dnmt3a-mediated targeted DNA methylation of APP rescues brain pathology in a mouse model of Alzheimer's disease. Translational Neurodegeneration 2022 11:1.

[26] Gemberling MP, Siklenka K, Rodriguez E, Tonn-Eisinger KR, Barrera A, Liu F (2021). Transgenic mice for *in vivo* epigenome editing with CRISPR-based systems. Nature Methods 2021 18:8.

[27] O'geen H, Tomkova M, Combs JA, Tilley EK, Segal DJ (2022). Determinants of heritable gene silencing for KRAB-dCas9 + DNMT3 and Ezh2-dCas9 + DNMT3 hit-and-run epigenome editing. Nucleic Acids Research.

[29] Seijas A, Cora D, Novo M, Al-Soufi W, Sánchez L, Arana ÁJ (2025). CRISPR/Cas9 Delivery Systems to Enhance Gene Editing Efficiency. International Journal of Molecular Sciences 2025, Vol 26.

[30] Honrath S, Burger M, Leroux JC (2025). Hurdles to healing: Overcoming cellular barriers for viral and nonviral gene therapy. International Journal of Pharmaceutics.

[31] Dong W, Kantor B, Dong W, Kantor B (2021). Lentiviral Vectors for Delivery of Gene-Editing Systems Based on CRISPR/Cas: Current State and Perspectives. Viruses 2021, Vol 13.

[32] Kantor B, O'Donovan B, Chiba-Falek O (2025). Trends and challenges of AAV-delivered gene editing therapeutics for CNS disorders: Implications for neurodegenerative disease. Molecular Therapy Nucleic Acids.

[33] Eshghi S, Mousakhan Bakhtiari M, Behfar M, Izadi E, Naji P, Jafari L (2025). Viral-based gene therapy clinical trials for immune deficiencies and blood disorders from 2013 until 2023 - an overview. Regenerative Therapy.

[34] Foley RA, Sims RA, Duggan EC, Olmedo JK, Ma R, Jonas SJ (2022). Delivering the CRISPR/Cas9 system for engineering gene therapies: Recent cargo and delivery approaches for clinical translation. Frontiers in Bioengineering and Biotechnology.

[35] Taghdiri M, Mussolino C, Taghdiri M, Mussolino C (2024). Viral and Non-Viral Systems to Deliver Gene Therapeutics to Clinical Targets. International Journal of Molecular Sciences 2024, Vol 25.

[36] Rauf MA, Rao A, Sivasoorian SS, Iyer AK (2025). Nanotechnology-Based Delivery of CRISPR/Cas9 for Cancer Treatment: A Comprehensive Review. Cells.

[37] Ge H, Shi Z, Liu C, Lu J, Yao Y, Cheng B (2025). Viral and non-viral vectors for gene therapy in the treatment of bone-related disorders: molecular insights and clinical perspectives. Molecular Aspects of Medicine.

[38] Cheng Q, Wei T, Farbiak L, Johnson LT, Dilliard SA, Siegwart DJ (2020). Selective organ targeting (SORT) nanoparticles for tissue-specific mRNA delivery and CRISPR-Cas gene editing. Nature Nanotechnology 2020 15:4.

[39] Hosseini-Kharat M, Bremmell KE, Prestidge CA (2025). Why do lipid nanoparticles target the liver? Understanding of biodistribution and liver-specific tropism. Molecular Therapy Methods and Clinical Development. 2025 Mar 13;33(1). doi:10.1016/j. omtm.

[40] Palakurthi SS, Shah B, Kapre S, Charbe N, Immanuel S, Pasham S (2024). A comprehensive review of challenges and advances in exosome-based drug delivery systems. Nanoscale Advances.

[41] Guzmán-Sastoque P, Rodríguez CF, Monsalve MC, Castellanos S, Manrique-Moreno A, Reyes LH (2025). Nanotheranostics Revolutionizing Gene Therapy: Emerging Applications in Gene Delivery Enhancement. Journal of Nanotheranostics 2025, Vol 6.

[42] Raguram A, Banskota S, Liu DR (2022). Therapeutic *in vivo* delivery of gene editing agents. Cell.

[43] Honrath S, Burger M, Leroux JC (2025). Hurdles to healing: Overcoming cellular barriers for viral and nonviral gene therapy. International Journal of Pharmaceutics.

[44] Sinclair F, Begum AA, Dai CC, Toth I, Moyle PM (2023). Recent advances in the delivery and applications of nonviral CRISPR/Cas9 gene editing. Drug Delivery and Translational Research 2023 13:5.

[45] Tsuchida CA, Wasko KM, Hamilton JR, Doudna JA, Seidman CE (2024). Targeted nonviral delivery of genome editors *in vivo*. Proceedings of the National Academy of Sciences of the United States of America.

[46] Wu F, Li N, Xiao Y, Palanki R, Yamagata H, Mitchell MJ (2025). Lipid Nanoparticles for Delivery of CRISPR Gene Editing Components. Small Methods.

[47] Mendes BB, Conniot J, Avital A, Yao D, Jiang X, Zhou X (2022). Nanodelivery of nucleic acids. Nat Rev Methods Primers.

[48] Liu F, Song YK, Liu D (1999). Hydrodynamics-based transfection in animals by systemic administration of plasmid DNA. Gene therapy.

[49] Niola F, Dagnæs-Hansen F, Frödin M (2019). *In vivo* Editing of the Adult Mouse Liver Using CRISPR/Cas9 and Hydrodynamic Tail Vein Injection. Methods in molecular biology (Clifton, NJ).

[50] Xue W, Chen S, Yin H, Tammela T, Papagiannakopoulos T, Joshi NS (2014). CRISPR-mediated direct mutation of cancer genes in the mouse liver. Nature.

[51] Yin H, Xue W, Chen S, Bogorad RL, Benedetti E, Grompe M (2014). Genome editing with Cas9 in adult mice corrects a disease mutation and phenotype. Nature biotechnology.

[52] Suda T, Liu D (2007). Hydrodynamic gene delivery: Its principles and applications. Molecular Therapy.

[53] Niu Y, Shen B, Cui Y, Chen Y, Wang J, Wang L (2014). Generation of gene-modified cynomolgus monkey via Cas9/RNA-mediated gene targeting in one-cell embryos. Cell.

[54] Zhao H, Tu Z, Xu H, Yan S, Yan H, Zheng Y (2017). Altered neurogenesis and disrupted expression of synaptic proteins in prefrontal cortex of SHANK3-deficient non-human primate. Cell research.

[55] Chang N, Sun C, Gao L, Zhu D, Xu X, Zhu X (2013). Genome editing with RNA-guided Cas9 nuclease in zebrafish embryos. Cell research.

[56] Qin W, Dion SL, Kutny PM, Zhang Y, Cheng AW, Jillette NL (2015). Efficient CRISPR/cas9-mediated genome editing in mice by zygote electroporation of nuclease. Genetics.

[57] Cavazza A, Molina-Estévez FJ, Reyes ÁP, Ronco V, Naseem A, Malenšek Š (2025). Advanced delivery systems for gene editing: A comprehensive review from the GenE-HumDi COST Action Working Group. Molecular Therapy Nucleic Acids. 2025 Mar 11;36(1). doi:10.1016/j. omtn.

[58] Kim GB, Rincon Fernandez Pacheco D, Saxon D, Yang A, Sabet S, Dutra-Clarke M (2019). Rapid Generation of Somatic Mouse Mosaics with Locus-Specific, Stably Integrated Transgenic Elements. Cell.

[59] Latella MC, Di Salvo MT, Cocchiarella F, Benati D, Grisendi G, Comitato A (2016). *In vivo* Editing of the Human Mutant Rhodopsin Gene by Electroporation of Plasmid-based CRISPR/Cas9 in the Mouse Retina. Mol Ther Nucleic Acids.

[60] Cwetsch AW, Gil-Sanz C (2025). Electroporation Techniques to Target Subependymal Zone Neurogenic Niche. Methods Mol Biol.

[61] Xu L, Park KH, Zhao L, Xu J, El Refaey M, Gao Y (2016). CRISPR-mediated Genome Editing Restores Dystrophin Expression and Function in mdx Mice. Mol Ther.

[62] de Morais CCP de L, Correia EM, Bonamino MH, de Vasconcelos ZFM (2024). Cell-Penetrating Peptides and CRISPR-Cas9: A Combined Strategy for Human Genetic Disease Therapy. Hum Gene Ther.

[63] Patel SG, Sayers EJ, He L, Narayan R, Williams TL, Mills EM (2019). Cell-penetrating peptide sequence and modification dependent uptake and subcellular distribution of green florescent protein in different cell lines. Sci Rep.

[65] Foss DV, Muldoon JJ, Nguyen DN, Carr D, Sahu SU, Hunsinger JM (2023). Peptide-mediated delivery of CRISPR enzymes for the efficient editing of primary human lymphocytes. Nat Biomed Eng.

[66] Zuris JA, Thompson DB, Shu Y, Guilinger JP, Bessen JL, Hu JH (2015). Cationic lipid-mediated delivery of proteins enables efficient protein-based genome editing in vitro and *in vivo*. Nat Biotechnol.

[67] Dos Santos Rodrigues B, Kanekiyo T, Singh J (2020). In vitro and *in vivo* characterization of CPP and transferrin modified liposomes encapsulating pDNA. Nanomedicine: Nanotechnology, Biology and Medicine.

[68] Bolhassani A, Jafarzade BS, Mardani G (2017). In vitro and *in vivo* delivery of therapeutic proteins using cell penetrating peptides. Peptides.

[69] Mohammadian Farsani A, Mokhtari N, Nooraei S, Bahrulolum H, Akbari A, Farsani ZM (2024). Lipid nanoparticles: The game-changer in CRISPR-Cas9 genome editing. Heliyon.

[70] Behr M, Zhou J, Xu B, Zhang H (2021). *In vivo* delivery of CRISPR-Cas9 therapeutics: Progress and challenges. Acta Pharmaceutica Sinica B.

[71] Huang K, Zapata D, Tang Y, Teng Y, Li Y (2022). *In vivo* delivery of CRISPR-Cas9 genome editing components for therapeutic applications. Biomaterials. 2022 Dec 1;291. doi:10.1016/j. biomaterials.

[72] Wang X, Liu S, Sun Y, Yu X, Lee SM, Cheng Q (2023). Preparation of selective organ-targeting (SORT) lipid nanoparticles (LNPs) using multiple technical methods for tissue-specific mRNA delivery. Nature Protocols.

[73] Lee RG, Mazzola AM, Braun MC, Platt C, Vafai SB, Kathiresan S (2023). Efficacy and Safety of an Investigational Single-Course CRISPR Base-Editing Therapy Targeting PCSK9 in Nonhuman Primate and Mouse Models. Circulation.

[74] Laffin LJ, Nicholls SJ, Scott RS, Clifton PM, Baker J, Sarraju A (2025). Phase 1 Trial of CRISPR-Cas9 Gene Editing Targeting ANGPTL3. The New England journal of medicine.

[75] Haley RM, Padilla MS, El-Mayta RD, Joseph RA, Weber JA, Figueroa-Espada CG (2025). Lipid Nanoparticles for *In vivo* Lung Delivery of CRISPR-Cas9 Ribonucleoproteins Allow Gene Editing of Clinical Targets. ACS Nano.

[76] Yuan Z, Luozhong S, Li R, Gu W, Chen Y, Bhashyam D (2025). Transient Macrophage Depletion Circumvents Scavenging and Redirects Biodistribution of mRNA-Lipid Nanoparticles. ACS nano.

[77] Bai X, Chen Q, Li F, Teng Y, Tang M, Huang J (2024). Optimized inhaled LNP formulation for enhanced treatment of idiopathic pulmonary fibrosis via mRNA-mediated antibody therapy. Nat Commun.

[78] Vasudevan A, Jozić A, Curtis AG, Bodi E, Ryals RC, Sahay G (2025). Lipid nanoparticle-mediated intracameral mRNA delivery facilitates gene expression and editing in the anterior chamber of the eye. Journal of Controlled Release.

[79] Mrksich K, Padilla MS, Mitchell MJ (2024). Breaking the final barrier: Evolution of cationic and ionizable lipid structure in lipid nanoparticles to escape the endosome. Advanced Drug Delivery Reviews. 2024 Nov 1;214. doi:10.1016/j. addr.

[80] Sarkar S, Moitra P, Duan W, Bhattacharya S (2024). A Multifunctional Aptamer Decorated Lipid Nanoparticles for the Delivery of EpCAM-targeted CRISPR/Cas9 Plasmid for Efficacious *In vivo* Tumor Regression. Advanced healthcare materials.

[81] Zhao X, Chen J, Qiu M, Li Y, Glass Z, Xu Q (2020). Imidazole-Based Synthetic Lipidoids for *In vivo* mRNA Delivery into Primary T Lymphocytes. Angewandte Chemie (International ed in English).

[82] Xue L, Gong N, Shepherd SJ, Xiong X, Liao X, Han X (2022). Rational Design of Bisphosphonate Lipid-like Materials for mRNA Delivery to the Bone Microenvironment. Journal of the American Chemical Society.

[83] Chen K, Han H, Zhao S, Xu B, Yin B, Lawanprasert A (2025). Lung and liver editing by lipid nanoparticle delivery of a stable CRISPR-Cas9 ribonucleoprotein. Nat Biotechnol.

[84] Jiang Y, Chen S, Hsiao S, Zhang H, Xie D, Wang ZJ (2025). Efficient and safe *in vivo* treatment of primary hyperoxaluria type 1 via LNP-CRISPR-Cas9-mediated glycolate oxidase disruption. Mol Ther.

[85] Musunuru K, Grandinette SA, Wang X, Hudson TR, Briseno K, Berry AM (2025). Patient-Specific *In vivo* Gene Editing to Treat a Rare Genetic Disease. The New England journal of medicine.

[86] He X, Yan T, Song Z, Xiang L, Xiang J, Yang Y (2025). Correcting a patient-specific Rhodopsin mutation with adenine base editor in a mouse model. Molecular Therapy.

[87] Lu X, Zhu Y, Wei C, Cheng L, Goodier KD, Kong J (2025). A multistep platform identifies spleen-tropic lipid nanoparticles for *in vivo* T cell-targeted delivery of gene-editing proteins. Science Advances.

[88] Gonzalez JC, Park KW, Evans DB, Sharma R, Sahaym O, Gopalakrishnan S (2025). Nano Approaches to Nucleic Acid Delivery: Barriers, Solutions, and Current Landscape. WIREs Nanomed Nanobiotechnol.

[89] Luo J, Cui Y, Xu L, Zhang J, Chen J, Li X (2025). Layered double hydroxides for regenerative nanomedicine and tissue engineering: recent advances and future perspectives. J Nanobiotechnology.

[90] Liu X, Gao M, Bao J (2025). Precisely Targeted Nanoparticles for CRISPR-Cas9 Delivery in Clinical Applications. Nanomaterials.

[91] Lee H, Rho WY, Kim YH, Chang H, Jun BH (2025). CRISPR-Cas9 Gene Therapy: Non-Viral Delivery and Stimuli-Responsive Nanoformulations. Molecules.

[92] Azeez SS, Hamad RS, Hamad BK, Shekha MS, Bergsten P (2024). Advances in CRISPR-Cas technology and its applications: revolutionising precision medicine. Front Genome Ed. 2024 Dec 12;6. doi:10.3389/fgeed.

[93] Rouatbi N, McGlynn T, Al-Jamal KT (2022). Pre-clinical non-viral vectors exploited for *in vivo* CRISPR/Cas9 gene editing: an overview. Biomater Sci.

[94] Liang C, Li F, Wang L, Zhang ZK, Wang C, He B (2017). Tumor cell-targeted delivery of CRISPR/Cas9 by aptamer-functionalized lipopolymer for therapeutic genome editing of VEGFA in osteosarcoma. Biomaterials.

[95] Blanchard EL, Vanover D, Bawage SS, Tiwari PM, Rotolo L, Beyersdorf J (2021). Treatment of influenza and SARS-CoV-2 infections via mRNA-encoded Cas13a in rodents. Nat Biotechnol.

[96] Zhu D, Shen H, Tan S, Hu Z, Wang L, Yu L (2018). Nanoparticles Based on Poly (β-Amino Ester) and HPV16-Targeting CRISPR/shRNA as Potential Drugs for HPV16-Related Cervical Malignancy. Mol Ther.

[97] Zhang X, Xu C, Gao S, Li P, Kong Y, Li T (2019). CRISPR/Cas9 Delivery Mediated with Hydroxyl-Rich Nanosystems for Gene Editing in Aorta. Advanced Science.

[98] Palmerston Mendes L, Pan J, Torchilin VP (2017). Dendrimers as Nanocarriers for Nucleic Acid and Drug Delivery in Cancer Therapy. Molecules.

[99] Zhou W, Cui H, Ying L, Yu XF (2018). Enhanced Cytosolic Delivery and Release of CRISPR/Cas9 by Black Phosphorus Nanosheets for Genome Editing. Angew Chem Int Ed Engl.

[100] Lee K, Conboy M, Park HM, Jiang F, Kim HJ, Dewitt MA (2017). Nanoparticle delivery of Cas9 ribonucleoprotein and donor DNA *in vivo* induces homology-directed DNA repair. Nat Biomed Eng.

[101] Lee B, Lee K, Panda S, Gonzales-Rojas R, Chong A, Bugay V (2018). Nanoparticle delivery of CRISPR into the brain rescues a mouse model of fragile X syndrome from exaggerated repetitive behaviours. Nature biomedical engineering.

[102] Tsuchida CA, Wasko KM, Hamilton JR, Doudna JA (2024). Targeted nonviral delivery of genome editors *in vivo*. Proceedings of the National Academy of Sciences.

[103] Noureddine A, Maestas-Olguin A, Saada EA, LaBauve AE, Agola JO, Baty KE (2020). Engineering of monosized lipid-coated mesoporous silica nanoparticles for CRISPR delivery. Acta Biomaterialia.

[104] Kim HS, Kweon J, Kim Y (2024). Recent advances in CRISPR-based functional genomics for the study of disease-associated genetic variants. Exp Mol Med.

[105] Wang Y, Wang X, Xie R, Burger JC, Tong Y, Gong S (2023). Overcoming the Blood-Brain Barrier for Gene Therapy via Systemic Administration of GSH-Responsive Silica Nanocapsules. Advanced materials (Deerfield Beach, Fla).

[106] Tng DJH, Low JGH (2023). Current status of silica-based nanoparticles as therapeutics and its potential as therapies against viruses. Antiviral Res.

[107] Wang Z, Wei L, Chen Y (2024). Magnetic particles-integrated CRISPR/Cas systems for biosensing. TrAC Trends in Analytical Chemistry.

[108] Wang M, Li D, Zhu J, Liu J, Yin Y, Su Y (2024). Recent advances on two-dimensional material-based nanosystems for gene delivery. APL Materials.

[109] Bian Y, Cai X, Lv Z, Xu Y, Wang H, Tan C (2023). Layered Double Hydroxides: A Novel Promising 2D Nanomaterial for Bone Diseases Treatment. Adv. Sci.

[110] Chen T, Chen D, Su W, Liang J, Liu X, Cai M (2025). Extracellular vesicles as vital players in drug delivery: a focus on clinical disease treatment. Frontiers in Bioengineering and Biotechnology.

[111] Kumar MA, Baba SK, Sadida HQ, Marzooqi SA, Jerobin J, Altemani FH (2024). Extracellular vesicles as tools and targets in therapy for diseases. Signal Transduction and Targeted Therapy 2024 9:1.

[112] Huang X, Li A, Xu P, Yu Y, Li S, Hu L (2023). Current and prospective strategies for advancing the targeted delivery of CRISPR/Cas system via extracellular vesicles. Journal of Nanobiotechnology 2023 21:1.

[113] Lu Y, Godbout K, Lamothe G, Tremblay JP (2023). CRISPR-Cas9 delivery strategies with engineered extracellular vesicles. Molecular Therapy Nucleic Acids.

[114] Pan X, Li Y, Huang P, Staecker H, He M (2024). Extracellular vesicles for developing targeted hearing loss therapy. Journal of Controlled Release.

[115] Dubey S, Chen Z, Jiang YJ, Talis A, Molotkov A, Ali A (2024). Small extracellular vesicles (sEVs)-based gene delivery platform for cell-specific CRISPR/Cas9 genome editing. Theranostics.

[116] Ilahibaks NF, Kluiver TA, de Jong OG, de Jager SCA, Schiffelers RM, Vader P (2024). Extracellular vesicle-mediated delivery of CRISPR/Cas9 ribonucleoprotein complex targeting proprotein convertase subtilisin-kexin type 9 (Pcsk9) in primary mouse hepatocytes. Journal of Extracellular Vesicles.

[117] Gee P, Lung MSY, Okuzaki Y, Sasakawa N, Iguchi T, Makita Y (2020). Extracellular nanovesicles for packaging of CRISPR-Cas9 protein and sgRNA to induce therapeutic exon skipping. Nature Communications 2020 11:1.

[118] Mehdizadeh S, Mamaghani M, Hassanikia S, Pilehvar Y, Ertas YN (2025). Exosome-powered neuropharmaceutics: unlocking the blood-brain barrier for next-gen therapies. Journal of Nanobiotechnology 2025 23:1.

[119] van Haasteren J, Li J, Scheideler OJ, Murthy N, Schaffer DV (2020). The delivery challenge: fulfilling the promise of therapeutic genome editing. Nat Biotechnol.

[120] Bulcha JT, Wang Y, Ma H, Tai PWL, Gao G (2021). Viral vector platforms within the gene therapy landscape. Signal transduction and targeted therapy.

[121] Iwakuma T, Cui Y, Chang LJ (1999). Self-inactivating lentiviral vectors with U3 and U5 modifications. Virology.

[122] Engelman A, Englund G, Orenstein JM, Martin MA, Craigie R (1995). Multiple effects of mutations in human immunodeficiency virus type 1 integrase on viral replication. J Virol.

[123] Challis RC, Ravindra Kumar S, Chen X, Goertsen D, Coughlin GM, Hori AM (2022). Adeno-Associated Virus Toolkit to Target Diverse Brain Cells. Annu Rev Neurosci.

[124] Wang JH, Gessler DJ, Zhan W, Gallagher TL, Gao G (2024). Adeno-associated virus as a delivery vector for gene therapy of human diseases. Signal transduction and targeted therapy.

[125] Suzuki K, Matsuda T, Sasaki S, Klinman DM, Ishii N, Tozuka M (2006). Toll-Like Receptor Adaptor Molecules Enhance DNA-Raised Adaptive Immune Responses against Influenza and Tumors through Activation of Innate Immunity. Journal of Virology.

[126] McCarty DM, Fu H, Monahan PE, Toulson CE, Naik P, Samulski RJ (2003). Adeno-associated virus terminal repeat (TR) mutant generates self-complementary vectors to overcome the rate-limiting step to transduction *in vivo*. Gene Ther.

[127] Lee D, Liu J, Junn HJ, Lee EJ, Jeong KS, Seol DW (2019). No more helper adenovirus: production of gutless adenovirus (GLAd) free of adenovirus and replication-competent adenovirus (RCA) contaminants. Experimental & molecular medicine.

[128] Boucher P, Cui X, Curiel DT (2020). Adenoviral vectors for *in vivo* delivery of CRISPR-Cas gene editors. Journal of Controlled Release.

[129] Fisher KJ, Choi H, Burda J, Chen SJ, Wilson JM (1996). Recombinant Adenovirus Deleted of All Viral Genes for Gene Therapy of Cystic Fibrosis. Virology.

[130] Wang D, Zhang F, Gao G (2020). CRISPR-Based Therapeutic Genome Editing: Strategies and *In vivo* Delivery by AAV Vectors. Cell.

[131] Ehrke-Schulz E, Schiwon M, Leitner T (2017). CRISPR/Cas9 delivery with one single adenoviral vector devoid of all viral genes. Sci Rep.

[132] Waddington SN, McVey JH, Bhella D, Parker AL, Barker K, Atoda H (2008). Adenovirus Serotype 5 Hexon Mediates Liver Gene Transfer. Cell.

[133] Toietta G, Mane VP, Norona WS, Finegold MJ, Ng P, McDonagh AF (2005). Lifelong elimination of hyperbilirubinemia in the Gunn rat with a single injection of helper-dependent adenoviral vector. Proceedings of the National Academy of Sciences.

[134] Lee CS, Bishop ES, Zhang R, Yu X, Farina EM, Yan S (2017). Adenovirus-mediated gene delivery: Potential applications for gene and cell-based therapies in the new era of personalized medicine. Genes & Diseases.

[135] Lu ZH, Dmitriev IP, Brough DE, Kashentseva EA, Li J, Curiel DT (2020). A New Gorilla Adenoviral Vector with Natural Lung Tropism Avoids Liver Toxicity and Is Amenable to Capsid Engineering and Vector Retargeting. Journal of Virology.

[136] Bruder JT, Semenova E, Chen P, Limbach K, Patterson NB, Stefaniak ME (2012). Modification of Ad5 Hexon Hypervariable Regions Circumvents Pre-Existing Ad5 Neutralizing Antibodies and Induces Protective Immune Responses. PLOS ONE.

[137] Maeder ML, Stefanidakis M, Wilson CJ, Baral R, Barrera LA, Bounoutas GS (2019). Development of a gene-editing approach to restore vision loss in Leber congenital amaurosis type 10. Nat Med.

[138] Pierce EA, Aleman TS, Jayasundera KT, Ashimatey BS, Kim K, Rashid A (2024). Gene Editing for CEP290-Associated Retinal Degeneration. The New England journal of medicine.

[139] Sin TN, Tng N, Dragoli J, Ramesh Kumar S, Villafuerte-Trisolini C, Chung SH (2025). Safety and efficacy of CRISPR-mediated genome ablation of VEGFA as a treatment for choroidal neovascularization in nonhuman primate eyes. Molecular Therapy.

[140] Hanlon KS, Kleinstiver BP, Garcia SP, Zaborowski MP, Volak A, Spirig SE (2019). High levels of AAV vector integration into CRISPR-induced DNA breaks. Nat Commun.

[141] Singh K, Fronza R, Evens H, Chuah MK, VandenDriessche T (2024). Comprehensive analysis of off-target and on-target effects resulting from liver-directed CRISPR-Cas9-mediated gene targeting with AAV vectors. Mol Ther Methods Clin Dev.

[142] Bazick HO, Mao H, Niehaus JK, Wolter JM, Zylka MJ (2024). AAV vector-derived elements integrate into Cas9-generated double-strand breaks and disrupt gene transcription. Mol Ther.

[143] Simpson BP, Yrigollen CM, Izda A, Davidson BL (2023). Targeted long-read sequencing captures CRISPR editing and AAV integration outcomes in brain. Molecular Therapy.

[144] Muller A, Sullivan J, Schwarzer W, Wang M, Park-Windhol C, Hasler PW (2025). High-efficiency base editing in the retina in primates and human tissues. Nature Medicine 2025 31:2.

[145] Davis JR, Banskota S, Levy JM, Newby GA, Wang X, Anzalone AV (2024). Efficient prime editing in mouse brain, liver and heart with dual AAVs. Nature biotechnology.

[146] Burdo TH, Chen C, Kaminski R, Sariyer IK, Mancuso P, Donadoni M (2024). Correction: Preclinical safety and biodistribution of CRISPR targeting SIV in non-human primates. Gene Ther.

[147] Rauch BJ, DeLoughery A, Sper R, Chen S, Yunanda S, Masnaghetti M (2025). Single-AAV CRISPR editing of skeletal muscle in non-human primates with NanoCas, an ultracompact nuclease. bioRxiv. 2025 Jan 30.

[148] Booth C, Masiuk K, Vazouras K, Fernandes A, Xu-Bayford J, Campo Fernandez B (2025). Long-Term Safety and Efficacy of Gene Therapy for Adenosine Deaminase Deficiency. The New England journal of medicine.

[149] Jamil A, Qureshi Z, Siddique R, Altaf F, Selene I, Wali N (2024). Long-Term Outcomes and Adverse Events of CAR T-19 Cell Therapy in Relapsed or Refractory B-Cell Acute Lymphoblastic Leukemia - a Systematic Review and Meta-Analysis. Blood.

[150] Cong L, Ran FA, Cox D, Lin S, Barretto R, Habib N (2013). Multiplex genome engineering using CRISPR/Cas systems. Science (New York, NY).

[151] Shalem O, Sanjana NE, Hartenian E, Shi X, Scott DA, Mikkelsen TS (2014). Genome-scale CRISPR-Cas9 knockout screening in human cells. Science (New York, NY).

[152] Ling S, Yang S, Hu X, Yin D, Dai Y, Qian X (2021). Lentiviral delivery of co-packaged Cas9 mRNA and a Vegfa-targeting guide RNA prevents wet age-related macular degeneration in mice. Nature biomedical engineering.

[153] Ortinski PI, O'Donovan B, Dong X, Kantor B (2017). Integrase-Deficient Lentiviral Vector as an All-in-One Platform for Highly Efficient CRISPR/Cas9-Mediated Gene Editing. Molecular Therapy Methods and Clinical Development.

[154] Crudele JM, Chamberlain JS (2018). Cas9 immunity creates challenges for CRISPR gene editing therapies. Nature communications.

[155] Uchida N, Drysdale CM, Nassehi T, Gamer J, Yapundich M, DiNicola J (2021). Cas9 protein delivery non-integrating lentiviral vectors for gene correction in sickle cell disease. Molecular Therapy Methods and Clinical Development.

[156] Milani M, Fabiano A, Perez-Rodriguez M, Hernandez RJ, Zecchillo A, Zonari E (2025). *In vivo* haemopoietic stem cell gene therapy enabled by postnatal trafficking. Nature.

[157] An M, Raguram A, Du SW, Banskota S, Davis JR, Newby GA (2024). Engineered virus-like particles for transient delivery of prime editor ribonucleoprotein complexes *in vivo*. Nature biotechnology.

[158] Mangeot PE, Risson V, Fusil F, Marnef A, Laurent E, Blin J (2019). Genome editing in primary cells and *in vivo* using viral-derived Nanoblades loaded with Cas9-sgRNA ribonucleoproteins. Nature communications.

[159] Pankowicz FP, Barzi M, Legras X, Hubert L, Mi T, Tomolonis JA (2016). Reprogramming metabolic pathways *in vivo* with CRISPR/Cas9 genome editing to treat hereditary tyrosinaemia. Nature communications.

[160] Banskota S, Raguram A, Suh S, Du SW, Davis JR, Choi EH (2022). Engineered virus-like particles for efficient *in vivo* delivery of therapeutic proteins. Cell.

[161] Musunuru K, Chadwick AC, Mizoguchi T, Garcia SP, DeNizio JE, Reiss CW (2021). *In vivo* CRISPR base editing of PCSK9 durably lowers cholesterol in primates. Nature.

[162] Raguram A, An M, Chen PZ, Liu DR (2025). Directed evolution of engineered virus-like particles with improved production and transduction efficiencies. Nature biotechnology.

[163] Dipalo LL, Mikkelsen JG, Gijsbers R, Carlon MS (2025). Trojan Horse-Like Vehicles for CRISPR-Cas Delivery: Engineering Extracellular Vesicles and Virus-Like Particles for Precision Gene Editing in Cystic Fibrosis. Human Gene Therapy.

[164] Chen WJ, Cheng X, Fu Y, Zhao M, McGinley J, Westenberger A (2020). Rethinking monogenic neurological diseases. The BMJ.

[165] Devinsky O, Coller J, Ahrens-Nicklas R, Liu XS, Ahituv N, Davidson BL (2025). Gene therapies for neurogenetic disorders. Trends in Molecular Medicine.

[166] Pardridge WM (2016). CSF, blood-brain barrier, and brain drug delivery. Expert opinion on drug delivery.

[167] Engelhardt B, Sorokin L (2009). The blood-brain and the blood-cerebrospinal fluid barriers: function and dysfunction. Seminars in immunopathology.

[168] Pollina EA, Gilliam DT, Landau AT, Lin C, Pajarillo N, Davis CP (2023). A NPAS4-NuA4 complex couples synaptic activity to DNA repair. Nature.

[169] Dileep V, Boix CA, Mathys H, Marco A, Welch GM, Meharena HS (2023). Neuronal DNA double-strand breaks lead to genome structural variations and 3D genome disruption in neurodegeneration. Cell.

[170] Hana S, Peterson M, McLaughlin H, Marshall E, Fabian AJ, McKissick O (2021). Highly efficient neuronal gene knockout *in vivo* by CRISPR-Cas9 via neonatal intracerebroventricular injection of AAV in mice. Gene Ther.

[171] Swiech L, Heidenreich M, Banerjee A, Habib N, Li Y, Trombetta J (2015). *In vivo* interrogation of gene function in the mammalian brain using CRISPR-Cas9. Nat Biotechnol.

[172] Torregrosa T, Lehman S, Hana S, Marsh G, Xu S, Koszka K (2021). Use of CRISPR/Cas9-mediated disruption of CNS cell type genes to profile transduction of AAV by neonatal intracerebroventricular delivery in mice. Gene Therapy.

[173] Levy JM, Yeh WH, Pendse N, Davis JR, Hennessey E, Butcher R (2020). Cytosine and adenine base editing of the brain, liver, retina, heart and skeletal muscle of mice via adeno-associated viruses. Nature biomedical engineering.

[174] Alves CRR, Ha LL, Yaworski R, Lazzarotto CR, Christie KA, Reilly A (2023). Base editing as a genetic treatment for spinal muscular atrophy. bioRxiv : the preprint server for biology.

[175] Hatanaka F, Suzuki K, Shojima K, Yu J, Takahashi Y, Sakamoto A (2024). Therapeutic strategy for spinal muscular atrophy by combining gene supplementation and genome editing. Nature communications.

[176] Colasante G, Qiu Y, Massimino L, Di Berardino C, Cornford JH, Snowball A (2020). *In vivo* CRISPRa decreases seizures and rescues cognitive deficits in a rodent model of epilepsy. Brain : a journal of neurology.

[177] Zou Y, Sun X, Yang Q, Zheng M, Shimoni O, Ruan W (2022). Blood-brain barrier-penetrating single CRISPR-Cas9 nanocapsules for effective and safe glioblastoma gene therapy. Science advances.

[178] Metzger JM, Wang Y, Neuman SS, Snow KJ, Murray SA, Lutz CM (2023). Efficient *in vivo* neuronal genome editing in the mouse brain using nanocapsules containing CRISPR-Cas9 ribonucleoproteins. Biomaterials.

[179] Abbasi S, Uchida S, Toh K, Tockary TA, Dirisala A, Hayashi K (2021). Co-encapsulation of Cas9 mRNA and guide RNA in polyplex micelles enables genome editing in mouse brain. Journal of Controlled Release.

[180] Chen K, Stahl EC, Kang MH, Xu B, Allen R, Trinidad M (2024). Engineering self-deliverable ribonucleoproteins for genome editing in the brain. Nat Commun.

[181] Gao K, Han H, Cranick MG, Zhao S, Xu S, Yin B (2024). Widespread Gene Editing in the Brain via In Utero Delivery of mRNA Using Acid-Degradable Lipid Nanoparticles. ACS nano.

[182] Foust KD, Nurre E, Montgomery CL, Hernandez A, Chan CM, Kaspar BK (2009). Intravascular AAV9 preferentially targets neonatal neurons and adult astrocytes. Nat Biotechnol.

[183] Powell JE, Lim CKW, Krishnan R, McCallister TX, Saporito-Magriña C, Zeballos MA (2022). Targeted gene silencing in the nervous system with CRISPR-Cas13. Science advances.

[184] Salomonsson SE, Clelland CD (2024). Building CRISPR Gene Therapies for the Central Nervous System: A Review. JAMA neurology.

[185] Rohn TT, Radin D, Brandmeyer T, Linder BJ, Andriambeloson E, Wagner S (2023). Genetic modulation of the HTR2A gene reduces anxiety-related behavior in mice. PNAS nexus.

[186] Lao YH, Ji R, Zhou JK, Snow KJ, Kwon N, Saville E (2023). Focused ultrasound-mediated brain genome editing. Proceedings of the National Academy of Sciences of the United States of America.

[187] Wu SH, Li X, Qin DD, Zhang LH, Cheng TL, Chen ZF (2021). Induction of core symptoms of autism spectrum disorder by *in vivo* CRISPR/Cas9-based gene editing in the brain of adolescent rhesus monkeys. Science Bulletin.

[188] Bonowicz K, Jerka D, Piekarska K, Olagbaju J, Stapleton L, Shobowale M (2025). CRISPR-Cas9 in Cardiovascular Medicine: Unlocking New Potential for Treatment. Cells.

[189] Friedland AE, Baral R, Singhal P, Loveluck K, Shen S, Sanchez M (2015). Characterization of Staphylococcus aureus Cas9: a smaller Cas9 for all-in-one adeno-associated virus delivery and paired nickase applications. Genome Biology 2015 16:1.

[190] Zetsche B, Gootenberg JS, Abudayyeh OO, Slaymaker IM, Makarova KS, Essletzbichler P (2015). Cpf1 Is a Single RNA-Guided Endonuclease of a Class 2 CRISPR-Cas System. Cell.

[191] Lou Y, Yang P, Wang Y, Liu X, Guo Z, Geng Z (2024). Long-term therapeutic efficacy and safety profiles of hpCas13d RNA editing in treating early-onset hypertrophic cardiomyopathy. Life Sci.

[192] Mendell JR, Sahenk Z, Lehman K, Nease C, Lowes LP, Miller NF (2020). Assessment of Systemic Delivery of rAAVrh74.MHCK7.micro-dystrophin in Children With Duchenne Muscular Dystrophy: A Nonrandomized Controlled Trial. JAMA Neurol.

[193] Schmalkuche K, Rother T, Burgmann JM, Voß H, Höffler K, Dogan G (2024). Heart immunoengineering by lentiviral vector-mediated genetic modification during normothermic ex vivo perfusion. Frontiers in immunology. 2024;15. doi:10.3389/FIMMU.

[194] Gillmore JD, Gane E, Taubel J, Kao J, Fontana M, Maitland ML (2021). CRISPR-Cas9 *In vivo* Gene Editing for Transthyretin Amyloidosis. The New England journal of medicine.

[195] Brault J, Liu T, Liu S, Lawson A, Choi U, Kozhushko N (2022). CRISPR-Cas9-AAV versus lentivector transduction for genome modification of X-linked severe combined immunodeficiency hematopoietic stem cells. Frontiers in immunology. 2023 Jan 4;13. doi:10.3389/FIMMU.

[196] Tombácz I, Laczkó D, Shahnawaz H, Muramatsu H, Natesan A, Yadegari A (2021). Highly efficient CD4+ T cell targeting and genetic recombination using engineered CD4+ cell-homing mRNA-LNPs. Molecular Therapy.

[197] Breda L, Papp TE, Triebwasser MP, Yadegari A, Fedorky MT, Tanaka N (2023). *In vivo* hematopoietic stem cell modification by mRNA delivery. Science (New York, NY).

[198] Palanki R, Riley JS, Bose SK, Luks V, Dave A, Kus N (2024). In utero delivery of targeted ionizable lipid nanoparticles facilitates *in vivo* gene editing of hematopoietic stem cells. Proceedings of the National Academy of Sciences of the United States of America.

[199] Zhao JJ, Tian SN, Peng ZY, Ren JX, Zhang YY, Li GH (2025). Biomembrane-inspired lipid nanoparticles enhance CRISPR-Cas9 editing for hemophilia A. Journal of Controlled Release. 2025 Oct 10;386. doi:10.1016/j. jconrel.

[200] Qu J, Wang Y, Xiong C, Wang M, He X, Jia W (2024). *In vivo* gene editing of T-cells in lymph nodes for enhanced cancer immunotherapy. Nat Commun.

[201] Nyberg WA, Bernard PL, Ngo W, Wang CH, Ark J, Rothrock A (2026). *In vivo* site-specific engineering to reprogram T cells. Nature.

[202] Tessera Therapeutics [Internet] 2025 [cited 2026 Jan 27]. Tessera Therapeutics Features New Preclinical Data Demonstrating…. Available from: https://www.tesseratherapeutics.com/news/tessera-therapeutics-features-new-preclinical-data-demonstrating-progress-across-its-in-vivo-gene-writing-programs-and-delivery-platform-at-the-american-society-of-gene-and-cell-therapy-28th-annual-meeting.

[203] Chen MZ, Yuen D, McLeod VM, Yong KW, Smyth CH, Herling BR (2025). A versatile antibody capture system drives specific *in vivo* delivery of mRNA-loaded lipid nanoparticles. Nature nanotechnology.

[204] Mao K, Tan H, Cong X, Liu J, Xin Y, Wang J (2025). Optimized lipid nanoparticles enable effective CRISPR/Cas9-mediated gene editing in dendritic cells for enhanced immunotherapy. Acta Pharmaceutica Sinica B.

[205] Tian SC, Song XH, Feng KK, Li CL, Tu YF, Hu YS (2025). Self-oxygenating nanoplatform integrating CRISPR/Cas9 gene editing and immune activation for highly efficient photodynamic therapy. Journal of Colloid and Interface Science. 2025 Sep 1;693. doi:10.1016/j. jcis.

[206] O'Neill MJ, Bourre L, Melgar S, O'Driscoll CM (2011). Intestinal delivery of non-viral gene therapeutics: physiological barriers and preclinical models. Drug Discov Today.

[207] Zhang Y, Wang Y, Lu Y, Quan H, Wang Y, Song S (2025). Advanced oral drug delivery systems for gastrointestinal targeted delivery: the design principles and foundations. Journal of Nanobiotechnology 2025 23:1.

[208] Sheng H, Wu S, Xue Y, Zhao W, Caplan AB, Hovde CJ (2023). Engineering conjugative CRISPR-Cas9 systems for the targeted control of enteric pathogens and antibiotic resistance. PLOS ONE.

[209] Lam KN, Spanogiannopoulos P, Soto-Perez P, Alexander M, Nalley MJ, Bisanz JE (2021). Phage-delivered CRISPR-Cas9 for strain-specific depletion and genomic deletions in the gut microbiome. Cell Reports.

[210] Ghani MW, Iqbal A, Ghani H, Bibi S, Wang Z, Pei R (2023). Recent advances in nanocomposite-based delivery systems for targeted CRISPR/Cas delivery and therapeutic genetic manipulation. Journal of Materials Chemistry B.

[211] Skoufou-Papoutsaki N, Adler S, D'Santos P, Mannion L, Mehmed S, Kemp R (2023). Efficient genetic editing of human intestinal organoids using ribonucleoprotein-based CRISPR. DMM Disease Models and Mechanisms.

[212] Jefremow A, Neurath MF, Waldner MJ (2021). CRISPR/Cas9 in Gastrointestinal Malignancies. Frontiers in Cell and Developmental Biology.

[213] Kalter N, Fuster-García C, Silva A, Ronco-Díaz V, Roncelli S, Turchiano G (2025). Off-target effects in CRISPR-Cas genome editing for human therapeutics: Progress and challenges. Molecular Therapy Nucleic Acids.

[214] Ewaisha R, Anderson KS (2023). Immunogenicity of CRISPR therapeutics-Critical considerations for clinical translation. Frontiers in Bioengineering and Biotechnology.

[215] Amen RA, Hassan YM, Essmat RA, Ahmed RH, Azab MM, Shehata NR (2025). Harnessing the Microbiome: CRISPR-Based Gene Editing and Antimicrobial Peptides in Combating Antibiotic Resistance and Cancer. Probiotics Antimicrob Proteins.

[216] Asp P, Malachowska B, Guha C, Roy-Chowdhury J (2025). Liver-Directed Molecular Therapies: Current and Upcoming Strategies. Clin Liver Dis.

[217] Barzel A, Paulk NK, Shi Y, Huang Y, Chu K, Zhang F (2015). Promoterless gene targeting without nucleases ameliorates haemophilia B in mice. Nature.

[218] Borel F, Tang Q, Gernoux G, Greer C, Wang Z, Barzel A (2017). Survival Advantage of Both Human Hepatocyte Xenografts and Genome-Edited Hepatocytes for Treatment of α-1 Antitrypsin Deficiency. Mol Ther.

[219] Chandler RJ, Venturoni LE, Liao J, Hubbard BT, Schneller JL, Hoffmann V (2021). Promoterless, Nuclease-Free Genome Editing Confers a Growth Advantage for Corrected Hepatocytes in Mice With Methylmalonic Acidemia. Hepatology.

[220] De Giorgi M, Li A, Hurley A, Barzi M, Doerfler AM, Cherayil NA (2021). Targeting the Apoa1 locus for liver-directed gene therapy. Mol Ther Methods Clin Dev.

[221] Laoharawee K, DeKelver RC, Podetz-Pedersen KM, Rohde M, Sproul S, Nguyen HO (2018). Dose-Dependent Prevention of Metabolic and Neurologic Disease in Murine MPS II by ZFN-Mediated *In vivo* Genome Editing. Mol Ther.

[222] Pagant S, Huston MW, Moreira L, Gan L, St Martin S, Sproul S (2021). ZFN-mediated *in vivo* gene editing in hepatocytes leads to supraphysiologic α-Gal A activity and effective substrate reduction in Fabry mice. Mol Ther.

[223] A mutation-independent CRISPR-Cas9-mediated gene targeting approach to treat a murine model of ornithine transcarbamylase deficiency | Science Advances [Internet] [cited 2026 Feb 7]. Available from: https://www.science.org/doi/10.1126/sciadv.aax5701.

[224] Self-inactivating all-in-one AAV vectors for precision Cas9 genome editing via homology-directed repair in vivo | Nature Communications [Internet] [cited 2026 Feb 7]. Available from: https://www.nature.com/articles/s41467-021-26518-y.

[225] Wang Q, Zhong X, Li Q, Su J, Liu Y, Mo L (2020). CRISPR-Cas9-Mediated *In vivo* Gene Integration at the Albumin Locus Recovers Hemostasis in Neonatal and Adult Hemophilia B Mice. Mol Ther Methods Clin Dev.

[226] Song CQ, Wang D, Jiang T, O'Connor K, Tang Q, Cai L (2018). *In vivo* Genome Editing Partially Restores Alpha1-Antitrypsin in a Murine Model of AAT Deficiency. Hum Gene Ther.

[227] In vivo AAV-CRISPR/Cas9-Mediated Gene Editing Ameliorates Atherosclerosis in Familial Hypercholesterolemia | Circulation [Internet] [cited 2026 Feb 7]. Available from: https://www.ahajournals.org/doi/10.1161/CIRCULATIONAHA.119.042476.

[228] Yang Y, Wang L, Bell P, McMenamin D, He Z, White J (2016). A dual AAV system enables the Cas9-mediated correction of a metabolic liver disease in newborn mice. Nat Biotechnol.

[229] Arnson B, Ilich E, Beck T von, Li S, Brooks ED, Gheorghiu D (2025). Efficacious genome editing in infant mice with glycogen storage disease type Ia. JCI Insight.

[230] Richards DY, Winn SR, Dudley S, Nygaard S, Mighell TL, Grompe M (2020). AAV-Mediated CRISPR/Cas9 Gene Editing in Murine Phenylketonuria. Mol Ther Methods Clin Dev.

[231] Krooss SA, Dai Z, Schmidt F, Rovai A, Fakhiri J, Dhingra A (2020). Ex Vivo/*In vivo* Gene Editing in Hepatocytes Using “All-in-One” CRISPR-Adeno-Associated Virus Vectors with a Self-Linearizing Repair Template. iScience.

[232] Breton C, Furmanak T, Avitto AN, Smith MK, Latshaw C, Yan H (2021). Increasing the Specificity of AAV-Based Gene Editing through Self-Targeting and Short-Promoter Strategies. Mol Ther.

[233] CRISPR/Cas9-mediated glycolate oxidase disruption is an efficacious and safe treatment for primary hyperoxaluria type I | Nature Communications [Internet] [cited 2026 Feb 7]. Available from: https://www.nature.com/articles/s41467-018-07827-1.

[234] Martinez-Turrillas R, Martin-Mallo A, Rodriguez-Diaz S, Zapata-Linares N, Rodriguez-Marquez P, San Martin-Uriz P (2022). *In vivo* CRISPR-Cas9 inhibition of hepatic LDH as treatment of primary hyperoxaluria. Mol Ther Methods Clin Dev.

[235] Levy JM, Yeh WH, Pendse N, Davis JR, Hennessey E, Butcher R (2020). Cytosine and adenine base editing of the brain, liver, retina, heart and skeletal muscle of mice via adeno-associated viruses. Nat Biomed Eng.

[236] Packer MS, Chowdhary V, Lung G, Cheng LI, Aratyn-Schaus Y, Leboeuf D (2022). Evaluation of cytosine base editing and adenine base editing as a potential treatment for alpha-1 antitrypsin deficiency. Mol Ther.

[237] Villiger L, Rothgangl T, Witzigmann D, Oka R, Lin PJC, Qi W (2021). *In vivo* cytidine base editing of hepatocytes without detectable off-target mutations in RNA and DNA. Nat Biomed Eng.

[238] Wang Q, Liu J, Janssen JM, Tasca F, Mei H, Gonçalves MAFV (2021). Broadening the reach and investigating the potential of prime editors through fully viral gene-deleted adenoviral vector delivery. Nucleic Acids Res.

[239] Brooks DL, Whittaker MN, Said H, Dwivedi G, Qu P, Musunuru K (2024). A base editing strategy using mRNA-LNPs for *in vivo* correction of the most frequent phenylketonuria variant. HGG Adv.

[240] Chen Z, Kelly K, Cheng H, Dong X, Hedger AK, Li L (2023). *In vivo* Prime Editing by Lipid Nanoparticle Co-delivery of Chemically Modified pegRNA and Prime Editor mRNA. GEN Biotechnol.

[241] Deletion and replacement of long genomic sequences using prime editing | Nature Biotechnology [Internet] [cited 2026 Feb 7]. Available from: https://www.nature.com/articles/s41587-021-01026-y.

[242] Liu P, Liang SQ, Zheng C, Mintzer E, Zhao YG, Ponnienselvan K (2021). Improved prime editors enable pathogenic allele correction and cancer modelling in adult mice. Nat Commun.

[243] Böck D, Rothgangl T, Villiger L, Schmidheini L, Matsushita M, Mathis N (2022). *In vivo* prime editing of a metabolic liver disease in mice. Sci Transl Med.

[244] Rothgangl T, Tálas A, Ioannidi EI, Weber Y, Böck D, Matsushita M (2025). Treatment of a metabolic liver disease in mice with a transient prime editing approach. Nat Biomed Eng.

[245] Cappelluti MA, Mollica Poeta V, Valsoni S, Quarato P, Merlin S, Merelli I (2024). Durable and efficient gene silencing *in vivo* by hit-and-run epigenome editing. Nature.

[246] Maresch R, Mueller S, Veltkamp C, Öllinger R, Friedrich M, Heid I (2016). Multiplexed pancreatic genome engineering and cancer induction by transfection-based CRISPR/Cas9 delivery in mice. Nat Commun.

[247] Bevacqua RJ, Dai X, Lam JY, Gu X, Friedlander MSH, Tellez K (2021). CRISPR-based genome editing in primary human pancreatic islet cells. Nat Commun.

[248] Martinez S, Wu S, Geuenich M, Malik A, Weber R, Woo T (2024). *In vivo* CRISPR screens reveal SCAF1 and USP15 as drivers of pancreatic cancer. Nat Commun.

[249] Ionizable lipid nanoparticles deliver mRNA to pancreatic β cells via macrophage-mediated gene transfer [Internet] [cited 2026 Feb 7]. Available from: https://www.science.org/doi/10.1126/sciadv.ade1444 doi:10.1126/sciadv.ade1444.

[250] Liao H, Wu J, VanDusen NJ, Li Y, Zheng Y (2024). CRISPR-Cas9-mediated homology-directed repair for precise gene editing. Mol Ther Nucleic Acids.

[251] Đorđević M, Stepper P, Feuerstein-Akgoz C, Gerhauser C, Paunović V, Tolić A (2023). EpiCRISPR targeted methylation of Arx gene initiates transient switch of mouse pancreatic alpha to insulin-producing cells. Front Endocrinol. 2023 Mar 16;14. doi:10.3389/fendo.

[252] Alejandra GC, Lucia C, Ho HS, Luis G, Juan RP, Federico PB (2020). Activation of pancreatic β-cell genes by multiplex epigenetic CRISPR-editing [Internet]. bioRxiv; 2020 [cited 2026 Feb 7]. p.

[253] Wang Y, Xiang L, Su Z (2026). CRISPR/Cas9 gene editing in muscle-related genetic disorders: Restoring function and exercise capacity. Tissue and Cell.

[254] Ginjaar IB, Kneppers AL, v d Meulen JD, Anderson LV, Bremmer-Bout M, van Deutekom JC (2000). Dystrophin nonsense mutation induces different levels of exon 29 skipping and leads to variable phenotypes within one BMD family. Eur J Hum Genet.

[255] Schneider AFE, Tanganyika-de Winter CL, Jirka SMG, Tan X, Thompson EG, Ha K (2025). Using muscle homing peptide CyPep10 to deliver phosphorodiamidate morpholino oligomers in the mdx mouse. Molecular Therapy Nucleic Acids.

[256] Lim KRQ, Yoon C, Yokota T (2018). Applications of CRISPR/Cas9 for the Treatment of Duchenne Muscular Dystrophy. J Pers Med.

[257] Tabebordbar M, Lagerborg KA, Stanton A, King EM, Ye S, Tellez L (2021). Directed evolution of a family of AAV capsid variants enabling potent muscle-directed gene delivery across species. Cell.

[258] Duchêne BL, Cherif K, Iyombe-Engembe JP, Guyon A, Rousseau J, Ouellet DL (2018). CRISPR-Induced Deletion with SaCas9 Restores Dystrophin Expression in Dystrophic Models In Vitro and *In vivo*. Mol Ther.

[259] Kenjo E, Hozumi H, Makita Y, Iwabuchi KA, Fujimoto N, Matsumoto S (2021). Low immunogenicity of LNP allows repeated administrations of CRISPR-Cas9 mRNA into skeletal muscle in mice. Nat Commun.

[260] Moretti A, Fonteyne L, Giesert F, Hoppmann P, Meier AB, Bozoglu T (2020). Somatic gene editing ameliorates skeletal and cardiac muscle failure in pig and human models of Duchenne muscular dystrophy. Nat Med.

[261] Jin M, Lin J, Li H, Li Z, Yang D, Wang Y (2024). Correction of human nonsense mutation via adenine base editing for Duchenne muscular dystrophy treatment in mouse. Mol Ther Nucleic Acids.

[262] Adenine base editing in mouse embryos and an adult mouse model of Duchenne muscular dystrophy | Nature Biotechnology [Internet] [cited 2026 Feb 7]. Available from: https://www.nature.com/articles/nbt.4148.

[263] Yuan S, Larsson SC (2023). Epidemiology of sarcopenia: Prevalence, risk factors, and consequences. Metabolism.

[264] Baker P, Huang C, Radi R, Moll SB, Jules E, Arbiser JL (2023). Skin Barrier Function: The Interplay of Physical, Chemical, and Immunologic Properties. Cells.

[265] Baker C, Hayden MS (2020). Gene editing in dermatology: Harnessing CRISPR for the treatment of cutaneous disease. F1000Res.

[266] Yang X, Zhou S, Zeng J, Zhang S, Li M, Yue F (2024). A biodegradable lipid nanoparticle delivers a Cas9 ribonucleoprotein for efficient and safe in situ genome editing in melanoma. Acta Biomaterialia.

[267] Cho E, Yun S, Lee S, Kim M, Choi J, Choi SE (2025). Polymer- and Lipid-Based Nanostructures for Wound Healing with Barrier-Resolved Design. Pharmaceutics.

[268] Bolsoni J, Liu D, Mohabatpour F, Ebner R, Sadhnani G, Tafech B (2023). Lipid Nanoparticle-Mediated Hit-and-Run Approaches Yield Efficient and Safe In Situ Gene Editing in Human Skin. ACS Nano.

[269] Lotfi M, Morshedi Rad D, Mashhadi SS, Ashouri A, Mojarrad M, Mozaffari-Jovin S (2023). Recent Advances in CRISPR/Cas9 Delivery Approaches for Therapeutic Gene Editing of Stem Cells. Stem Cell Rev and Rep.

[270] Molaei Z, Jabbarpour Z, Omidkhoda A (2024). Exploring non-viral methods for the delivery of CRISPR-Cas ribonucleoprotein to hematopoietic stem cells. Stem Cell Res Ther.

[271] Guri-Lamce I, AlRokh Y, Kim Y, Maeshima R, Graham C, Hart SL (2024). Topical gene editing therapeutics using lipid nanoparticles: “gene creams” for genetic skin diseases?. Br J Dermatol.

[272] Ismail A, Chou SF (2025). Polyethylenimine Carriers for Drug and Gene Delivery. Polymers.

[273] Chen Y, Feng X (2022). Gold nanoparticles for skin drug delivery. Int J Pharm.

[274] Zheng B, Li Q, Fang L, Cai X, Liu Y, Duo Y (2024). Microorganism microneedle micro-engine depth drug delivery. Nat Commun.

[275] García M, Bonafont J, Martínez-Palacios J, Xu R, Turchiano G, Svensson S (2022). Preclinical model for phenotypic correction of dystrophic epidermolysis bullosa by *in vivo* CRISPR-Cas9 delivery using adenoviral vectors. Mol Ther Methods Clin Dev.

[276] Jiang Y, Chen S, Hsiao S, Zhang H, Xie D, Wang ZJ (2025). Efficient and safe *in vivo* treatment of primary hyperoxaluria type 1 via LNP-CRISPR-Cas9-mediated glycolate oxidase disruption. Molecular Therapy.

[277] CRISPR-Cas9-Mediated Correction of SLC12A3 Gene Mutation Rescues the Gitelman's Disease Phenotype in a Patient-Derived Kidney Organoid System [Internet] [cited 2026 Feb 7]. Available from: https://www.mdpi.com/1422-0067/24/3/3019.

[278] Wang J, Qiu Y, Zhang L, Zhou X, Hu S, Liu Q (2024). Adenine base editor corrected ADPKD point mutations in hiPSCs and kidney organoids. Adv Biotechnol (Singap).

[279] Hajarnis S, Lakhia R, Yheskel M, Williams D, Sorourian M, Liu X (2017). microRNA-17 family promotes polycystic kidney disease progression through modulation of mitochondrial metabolism. Nat Commun.

[280] Galichon P, Lannoy M, Li L, Serre J, Vandermeersch S, Legouis D (2024). Energy depletion by cell proliferation sensitizes the kidney epithelial cells to injury. Am J Physiol Renal Physiol.

[281] Hickey AJ, Pena ES, Maloney Norcross SE (2025). Aerosol particle design to promote targeting in the respiratory tract. European Journal of Pharmaceutical Sciences.

[282] Malainou C, Abdin SM, Lachmann N, Matt U, Herold S (2023). Alveolar macrophages in tissue homeostasis, inflammation, and infection: evolving concepts of therapeutic targeting. J Clin Invest.

[283] Du J, Wu Q, Liu C, Wang N, Gong C (2025). CRISPR delivery systems for organ-specific targeting: Advances and challenges. Precision Medicine and Engineering.

[284] Limberis MP, Vandenberghe LH, Zhang L, Pickles RJ, Wilson JM (2009). Transduction efficiencies of novel AAV vectors in mouse airway epithelium *in vivo* and human ciliated airway epithelium in vitro. Mol Ther.

[285] Thomas SP, Domm JM, van Vloten JP, Xu L, Vadivel A, Yates JGE (2023). A promoterless AAV6.2FF-based lung gene editing platform for the correction of surfactant protein B deficiency. Mol Ther.

[286] Qin Z, Yue M, Tang S, Wu F, Sun H, Li Y (2024). EML4-ALK fusions drive lung adeno-to-squamous transition through JAK-STAT activation. J Exp Med.

[287] Liu S, Wen Y, Shan X, Ma X, Yang C, Cheng X (2024). Charge-assisted stabilization of lipid nanoparticles enables inhaled mRNA delivery for mucosal vaccination. Nat Commun.

[288] In vivo editing of lung stem cells for durable gene correction in mice | Science [Internet] [cited 2026 Feb 7]. Available from: https://www.science.org/doi/10.1126/science.adk9428.

[289] Tian Z, Wang X, Chatterjee S (2026). Tripod-like' lung-targeting (LuT) lipids for highly efficient and selective LNPs for gene delivery and editing. Nat. Biomed. Eng.

[290] Sun H, Zhang Y, Wang J, Su J, Zhou D, Yu X (2023). Application of Lung-Targeted Lipid Nanoparticle-delivered mRNA of soluble PD-L1 via SORT Technology in Acute Respiratory Distress Syndrome. Theranostics.

[291] Nonviral CRISPR/Cas9 mutagenesis for streamlined generation of mouse lung cancer models [Internet] [cited 2026 Feb 7]. Available from: https://www.pnas.org/doi/10.1073/pnas.2322917121 doi: 10.1073/pnas.2322917121.

[292] Pinaud M, Zamborlini A (2025). Electroporation-Based CRISPR-Cas9-Mediated Gene Knockout in THP-1 Cells and Single-Cell Clone Isolation. Journal of Visualized Experiments.

[293] Mustafi D, Hisama FM, Huey J, Chao JR (2022). The Current State of Genetic Testing Platforms for Inherited Retinal Diseases. Ophthalmology Retina.

[294] Liu Z, Chen S, Lo CH, Wang Q, Sun Y (2024). All-in-one AAV-mediated Nrl gene inactivation rescues retinal degeneration in Pde6a mice. JCI Insight.

[295] Kellish PC, Marsic D, Crosson SM, Choudhury S, Scalabrino ML, Strang CE (2023). Intravitreal injection of a rationally designed AAV capsid library in non-human primate identifies variants with enhanced retinal transduction and neutralizing antibody evasion. Molecular Therapy.

[296] Patrizi C, Llado M, Benati D, Iodice C, Marrocco E, Guarascio R (2021). Allele-specific editing ameliorates dominant retinitis pigmentosa in a transgenic mouse model. American Journal of Human Genetics.

[297] Tsai YT, Wu WH, Lee TT, Wu WP, Xu CL, Park KS (2018). Clustered Regularly Interspaced Short Palindromic Repeats-Based Genome Surgery for the Treatment of Autosomal Dominant Retinitis Pigmentosa. Ophthalmology.

[298] Delplace V, Payne S, Shoichet M (2015). Delivery strategies for treatment of age-related ocular diseases: From a biological understanding to biomaterial solutions. Journal of Controlled Release.

[299] O'Leary F, Campbell M (2023). The blood-retina barrier in health and disease. The FEBS journal.

[300] Yiu G, Chung SH, Mollhoff IN, Nguyen UT, Thomasy SM, Yoo J (2020). Suprachoroidal and Subretinal Injections of AAV Using Transscleral Microneedles for Retinal Gene Delivery in Nonhuman Primates. Molecular Therapy Methods and Clinical Development.

[301] Bennett J (2017). Taking Stock of Retinal Gene Therapy: Looking Back and Moving Forward. Molecular Therapy.

[302] Du SW, Newby GA, Salom D, Gao F, Menezes CR, Suh S (2024). *In vivo* photoreceptor base editing ameliorates rhodopsin-E150K autosomal-recessive retinitis pigmentosa in mice. Proceedings of the National Academy of Sciences of the United States of America.

[303] Qin H, Zhang W, Zhang S, Feng Y, Xu W, Qi J (2023). Vision rescue via unconstrained *in vivo* prime editing in degenerating neural retinas. J Exp Med.

[304] Gautam M, Jozic A, Su GLN, Herrera-Barrera M, Curtis A, Arrizabalaga S (2023). Lipid nanoparticles with PEG-variant surface modifications mediate genome editing in the mouse retina. Nature communications.

[305] Wang Y, Shahi PK, Xie R, Zhang H, Abdeen AA, Yodsanit N (2020). A pH-responsive silica-metal-organic framework hybrid nanoparticle for the delivery of hydrophilic drugs, nucleic acids, and CRISPR-Cas9 genome-editing machineries. J Control Release.

[306] Kabra M, Shahi PK, Wang Y, Sinha D, Spillane A, Newby GA (2023). Nonviral base editing of KCNJ13 mutation preserves vision in a model of inherited retinal channelopathy. The Journal of clinical investigation.

[307] Frangoul H, Locatelli F, Sharma A, Bhatia M, Mapara M, Molinari L (2024). Exagamglogene Autotemcel for Severe Sickle Cell Disease. The New England journal of medicine.

[308] Gillmore JD, Gane E, Täubel J, Pilebro B, Echaniz-Laguna A, Kao J (2025). Nexiguran Ziclumeran Gene Editing in Hereditary ATTR with Polyneuropathy. N Engl J Med.

[309] Gu B, Li M, Li D, Huang K (2025). CRISPR-Cas9 Targeting PCSK9: A Promising Therapeutic Approach for Atherosclerosis. J Cardiovasc Transl Res.

[310] Uminski K, Goodyear D, Betschel S (2025). Therapeutic Advances in Hereditary Angioedema: A Focus on Present and Future Options. Adv Ther.

[311] Longhurst HJ, Lindsay K, Petersen RS, Fijen LM, Gurugama P, Maag D (2024). CRISPR-Cas9 *In vivo* Gene Editing of KLKB1 for Hereditary Angioedema. N Engl J Med.

[312] Letchumanan P, Theva Das K (2025). The role of genetic diversity, epigenetic regulation, and sex-based differences in HIV cure research: a comprehensive review. Epigenetics Chromatin.

[313] Kim P, Sanchez AM, Penke TJR, Tuson HH, Kime JC, McKee RW (2024). Safety, pharmacokinetics, and pharmacodynamics of LBP-EC01, a CRISPR-Cas3-enhanced bacteriophage cocktail, in uncomplicated urinary tract infections due to Escherichia coli (ELIMINATE): the randomised, open-label, first part of a two-part phase 2 trial. Lancet Infect Dis.

[315] Jin M, Ang Y, Metzloff AE, Thatte AS, Mitchell MJ (2026). Lipid nanoparticles for engineering next generation CAR T cell immunotherapy. Nanoscale Horizons.

[316] Research C for BE and (2026). Human Gene Therapy Products Incorporating Human Genome Editing [Internet]. FDA; 2024 [cited.

[317] Guideline on quality, non-clinical, clinical requirements for investigational advanced therapy medicinal products in clinical trials - Scientific guideline | European Medicines Agency (EMA) [Internet] 2019 [cited 2026 Jan 27]. Available from: https://www.ema.europa.eu/en/guideline-quality-non-clinical-clinical-requirements-investigational-advanced-therapy-medicinal-products-clinical-trials-scientific-guideline.

